# Supplementary material for: Cancer symptom experience and help-seeking behaviour during the COVID-19 pandemic in the UK: a cross-sectional population survey
Source: BMJ Open. 2021 Sep 16;11(9):e053095. doi: 10.1136/bmjopen-2021-053095 (PMC8449845; doi:10.1136/bmjopen-2021-053095)
Supplement: Supplementary data [file bmjopen-2021-053095supp001.pdf]

Supplementary File

Table S1: Barriers to consulting with a medical professional

Data are n (%) and unweighted unless otherwise stated

Barriers[1]	Pooled sample N=7,543	Pooled sample Weighted[2] N=7,543	At least one symptom experienced N=3,025	No symptoms experienced[3] n=4,428
I worried about wasting the healthcare professional's time	1,158 (15.4)	930 (12.3)	653 (21.6)	505 (11.4)
I worried about putting extra strain on the NHS	954 (12.6)	790 (10.5)	578 (19.1)	376 (8.5)
I didn't want to be seen as someone who makes a fuss	907 (12.0)	856 (11.3)	540 (17.9)	367 (8.3)
I found it difficult to get an appointment with a particular healthcare professional	774 (10.3)	627 (8.3)	448 (14.8)	326 (7.4)
I worried about catching coronavirus	721 (9.6)	632 (8.4)	415 (13.7)	306 (6.9)
I found it difficult to get an appointment at a convenient time	643 (8.5)	659 (8.7)	321 (10.6)	322 (7.3)
I worried they wouldn't take my symptom(s) seriously	601 (8.0)	574 (7.6)	380 (12.6)	221 (5.0)
I didn't want to talk to a receptionist/administrative person about my symptom(s)	518 (6.9)	458 (6.1)	304 (10.0)	214 (4.8)
I worried about what they might find wrong with me	421 (5.6)	452 (6.0)	231 (7.6)	190 (4.3)
I had too many other things to worry about	401 (5.3)	434 (5.8)	271 (9.0)	130 (2.9)
It would have been difficult for me to discuss my health problem remotely (by phone, email or video call)	361 (4.8)	319 (4.2)	231 (7.6)	130 (2.9)
I found it embarrassing talking about my symptoms	354 (4.7)	384 (5.1)	216 (7.1)	138 (3.1)
I was too busy to make time to seek medical attention	329 (4.4)	354 (4.7)	195 (6.4)	134 (30)
I worried about the possibility of having treatment	304 (4.0)	318 (4.2)	196 (6.5)	108 (2.4)
I didn't feel confident talking about my symptom(s)	272 (3.6)	309 (4.1)	160 (5.3)	112 (2.5)
I worried about the impact on my employment from taking time off	227 (3.0)	252 (3.3)	144 (4.8)	83 (1.9)
I had symptoms that might have been related to coronavirus	143 (1.9)	153 (2.0)	105 (3.5)	38 (0.9)
Nothing put me off/delayed me in seeking medical attention	3,039 (40.3)	2,845 (37.7)	859 (28.4)	2,180 (49.2)
Prefer not to say	114 (1.5)	130 (1.7)	31 (1.0)	83 (1.9)
**Number of barriers to help-seeking reported (0 to 17)**				
Median (25th to 75th centiles); Range	1.0 (1.0 to 2.0); (0 to 14)			

Quinn-Scoggins HD, *et al. BMJ Open* 2022; 11:e053095. doi: 10.1136/bmjopen-2021-053095

[1] Participants were asked: *"Thinking about the last time you considered seeing or speaking to a medical professional about your health, did any of the following put you off, or make you delay doing so? (This may have been an appointment with a medical professional (e.g. a doctor, nurse or pharmacist) in person, online or over the phone). Please select all that apply".* More than one barrier could be selected. Numbers do not amount to the denominator and percentages do not amount to 100%. [2] All data are weighted to match the adult population in the UK on age, gender, ethnicity, and region. [3] Includes those who preferred not to say.

Table S2. Help-seeking prompts to consulting with a medical professional *Data are n (%) and unweighted unless otherwise stated*

	COVID-CAM N=5,667	COVID-CAM Weighted[1] N=5,667
I had a symptom that I thought might be a sign of cancer	282 (5.0)	286 (5.0)
I had a symptom that was unusual for me	706 (12.5)	709 (12.5)
I had a symptom that was painful	811 (14.3)	815 (14.4)
I knew someone who had a similar symptom, and it turned out to be serious	122 (2.1)	119 (2.1)
I had a symptom that didn't go away	957 (16.9)	970 (17.1)
My friends or family encouraged me to go	461 (8.1)	454 (8.0)
I had a symptom, but I didn't know what was causing it	683 (12.1)	686 (12.1)
I had a symptom that was 'bothersome'	1,008 (17.8)	1,005 (17.7)
I had a feeling that something wasn't right	721 (12.7)	735 (13.0)
I had seen information about this symptom in the media	137 (2.4)	139 (2.5)
I could have a remote consultation (for example, by phone, email or video call)	448 (7.9)	447 (7.9)
I needed an appointment for a pre-existing problem/condition[2]	687 (12.1)	680 (12.0)
I needed help for a specific symptom or injury[2]	67 (1.2)	70 (1.2)
I needed a women's health appointment[2]	36 (0.6)	39 (0.7)
I had a symptom that was getting worse[2]	7 (0.1)	8 (0.1)
I needed to have a lab test or get a test result[2]	8 (0.1)	9 (0.2)
Other[3]	144 (2.5)	142 (2.58)
I have never sought medical attention	307 (5.4)	291 (5.1)
I don't remember	798 (14.1)	811 (14.3)
Prefer not to say	150 (2.6)	149 (2.6)

More than one **prompt** could be selected. Numbers do not amount to the denominator and percentages do not amount to 100%.

[1] All data are weighted to match the adult population in the UK on age, gender, ethnicity, and region. [2] Recoded from the 'other' free text option. [3] Reasons that could not be categorised into cohesive themes.

S3 - Interview Topic Guide

Participants will have access to study Information sheets and will have already provided informed consent. Prior to interview commencement the interviewer will re-confirm verbal consent.

The interview will be recorded, anonymised, transcribed confidentially and analysed by members of the research team.

The aim of the interview is to gain further understanding of how participants perceive symptoms, help seeking and behaviour regarding potential cancer symptoms during the COVID-19 lockdown from March 23rd 2020.

It is estimated the interview will be 45 minutes in length. Following the interview participants will be sent a £20 voucher to thank them for their time.

The following is a semi-structured topic guide. This is not a script - please feel free to amend wording/order and probe further on interesting points/topic where appropriate. Please also skip redundant questions based on previous answers where appropriate.

Topic Guide

General Health/Introduction

- How are you feeling today and generally?
- Has the pandemic and the lockdown affected your beliefs or feelings or concerns about your own health?
- Have you changed anything in your usual habits (eating, exercising, smoking) due to the coronavirus pandemic and lockdown?

Symptoms and Help seeking

- Since the start of the pandemic and UK lockdown in March this year, have you noticed any changes in your health/body?
    - Discuss decision to see/not see GP if appropriate
    - Discuss what their changes in their health/body were and what they did if appropriate
    - Discuss whether they were concerned about the changes if appropriate
    - Discuss whether they spoke to anyone about the changes
- How important do you think it is to talk to a doctor or healthcare professional if you (were to) notice a change in your health at the moment?
    - Discuss whether the pandemic has affected/changed this at all
- Did you have any concerns about attending a GP appointment in person during the pandemic?
- What do you think would encourage people to contact their GP to check on change to their health or body?
- If you did speak to your GP or a healthcare professioanl during the pandemic, how did you talk to them?
    - Discuss how people felt about face-to-face appointments or virtual/remote consultations at the moment, whether it worked for them and whether they felt satisfied
- What are your thoughts about phone or video (remote) consulting?
- How did getting help or advice from your GP or a healthcare professional compare to what you experienced before?

- If your GP or healthcare professional referred you to the hospital, how would you feel about attending during the pandemic?
    - Discuss what risks they would consider when thinking about attending and what would give them more confidence to visit if appropriate
- Do you have any concerns about possible delays in getting treatment for other illness due to pandemic and lockdown?

Screening

- Do you take part in any regular screening programmes? Which ones?
- Should you have been invited for any screening since the start of lockdown in March? Was your screening put on hold or delayed at all?
    - Discuss what happened with their screening, how they feel about postponing/delays and whether this makes them think any differently about screening at all if appropriate
- What do you think would have been the best way to go about screening during the pandemic and lockdown?
    - Discuss whether they believe it should have gone ahead as usual, whether they would have attended as usual/for the first time, whether they would have had any concerns/felt safe and would they expect a delay in their results if appropriate
- Has the pandemic and lockdown changed the way you think about screening in any way?
- What do you think would encourage people to consider taking part in screening again?
- If we invite people who have been waiting or had their screening delayed during the pandemic and lockdown how do you think it should be prioritised?

Health behaviours and Prevention

[Confirm smoking status]

- Has your smoking changed at all during the pandemic and lockdown?
    - Discuss what changed, how, why and when if appropriate
- What do you think about the links which have been shown between heavier body weight and the coronavirus infection?
- What do you think about the links between heavier body weight and other serious illnesses?

Health messaging

- During the pandemic and lockdown where did you get most of your health information from and why?
- How useful and believable did you find these information sources?
    - Discuss which information sources gave them the most confidence in knowing what to do during the pandemic and lockdown regarding health/safety if appropriate
- How useful was social media to you? Did you use them to find or access information at all?
- How did your views of health information change (if at all) during the pandemic and lockdown?

    - Discuss whether they felt that viewed anything unreliable about health/healthcare or COVID-19 and whether they were suspicious of 'fake news' at anytime and how they acted upon these if appropriate
- Did you discuss the health messages you have seen with anyone in person or online?

Closing

- What was the most important thing to consider for your own health (and your family/friends if applicable) throughout the pandemic and lockdown? Did anything influence your thoughts about this?

  [Thank the participant for their time and check if there is anything they would like to expand on or anything they would like to mention that we have not discussed]

Table S4: Help-seeking interval by symptom

Data are n (%) and unweighted unless otherwise stated

Symptom	Had symptom[1]	Contacted the GP	Within 1 week[2]	Within 2 weeks[2]	Within 1 month[2]	Within 6 weeks[2]	Within 3 months[2]	Within 6 months[2]	Contacted within recommended interval[a,b,3]
	n / 7,543 (%)	n / S (%)	n / S (%)	n / S (%)	n / S (%)	n / S (%)	n / S (%)	n / S (%)	n / S (%)
**Non-specific symptom**									
A persistent change in bowel habits[a]	541 (7.2)	254 (47.0)	55 (10.2)	51 (9.4)	42 (7.8)	30 (5.5)	24 (4.4)	52 (9.6)	148 (27.4)
A persistent change in bladder habits[a]	450 (6.0)	227 (50.4)	65 (14.4)	43 (9.6)	32 (7.1)	23 (5.1)	29 (6.4)	35 (7.8)	140 (31.1)
Tired all the time[a]	1,603 (21.3)	540 (33.7)	92 (5.7)	79 (4.9)	95 (5.9)	58 (3.6)	81 (5.1)	135 (8.4)	266 (16.6)
Persistent unexplained pain[a]	662 (8.8)	361 (54.5)	74 (11.2)	68 (10.3)	59 (8.9)	39 (5.9)	52 (7.9)	69 (10.4)	201 (30.4)
**Non-specific/Red flag symptom**									
Unexplained weight loss[a]	395 (5.2)	179 (45.3)	48 (12.2)	45 (11.4)	43 (10.9)	19 (4.8)	10 (2.5)	14 (3.5)	136 (34.4)
**Red flag symptom**									
A change in the appearance of a mole[b]	391 (5.2)	157 (40.2)	37 (9.5)	34 (8.7)	27 (6.9)	18 (4.6)	20 (5.1)	21 (5.4)	71 (18.2)
An unexplained lump or swelling[b]	422 (5.6)	239 (56.6)	81 (19.2)	55 (13.0)	42 (10.0)	11 (2.6)	19 (4.5)	31 (7.3)	136 (32.2)
Unexplained bleeding[b]	267 (3.5)	143 (53.6)	55 (20.6)	22 (8.2)	22 (8.2)	22 (8.2)	8 (3.0)	14 (5.2)	77 (28.8)
A persistent difficulty swallowing[b]	237 (3.1)	128 (54.0)	26 (11.0)	26 (11.0)	31 (13.1)	12 (5.1)	11 (4.6)	22 (9.3)	52 (21.9)
A sore that does not heal[b]	291 (3.9)	128 (44.0)	34 (11.7)	27 (9.3)	22 (7.6)	12 (4.1)	13 (4.5)	20 (6.9)	61 (21.0)

**Red flag/Lung-specific symptom**									
Coughing up blood[b]	114 (1.5)	67 (58.8)	32 (28.1)	11 (9.6)	10 (8.8)	5 (4.4)	5 (4.4)	4 (3.5)	43 (37.7)
**Lung-specific symptom**									
Shortness of breath[a]	1,052 (13.9)	484 (46.0)	123 (11.7)	69 (6.6)	100 (9.5)	50 (4.8)	49 (4.7)	93 (8.8)	292 (27.8)
Persistent hoarseness[a]	200 (2.7)	96 (48.0)	25 (12.5)	18 (9.0)	19 (9.5)	13 (6.5)	8 (4.0)	13 (6.5)	62 (31.0)
A persistent cough[a]	444 (5.9)	230 (51.8)	52 (11.7)	40 (9.0)	46 (10.4)	21 (4.7)	29 (6.5)	42 (9.5)	138 (31.1)
A change in an existing cough[a]	196 (2.6)	100 (51.0)	31 (15.8)	24 (12.2)	19 (9.7)	17 (8.7)	5 (2.6)	4 (2.0)	74 (37.8)

n=number, n/S = number of participants representing each symptom presentation behaviour/number of participants who had this symptom.

[a] Recommended interval <1 month, [b] Recommended interval <2 weeks based on previous studies of cancer symptom presentation behaviour (e.g. Herbert et al., 2018 https://www·thelancet·com/journals/lanchi/article/PIIS2352-4642(18)30004-X/fulltext) and aligned with cancer awareness campaigns (e.g. Be Clear on Cancer https://www·cancerresearchuk·org/health-professional/awareness-and-prevention/be-clear-on-cancer). [1] Denominator includes those who did not have a symptom and those who preferred not to say (around 1% of the sample). [2] Interval contacted GP from noticing the symptom. [3] Subset of participants who 'Contacted the GP in the last 6 months'.

Table S5: Definitions of codes identified during qualitative interviews with participants and mapping of these themes of symptom experiences, fear of help-seeking and experiences of help-seeking

Themes	Codes	Code definitions
Symptom Experiences	The impact of the pandemic and/or lockdown on general health	Any discussion or reference relating to if and how the pandemic and/or lockdown has impacted the participants general health. Includes changes on beliefs, feelings, views and concerns – relating to both physical and mental health
Symptom Experiences	Symptom experience and recognising changes in health or body	Any discussion or reference to the participant recognising changes to their health or body during the pandemic.
Experiences of help-seeking	Actions taken, or not taken, due to changes in health or body	Any discussion or reference made by the participant to actions taken due to changes in their health or body. Includes healthcare and non-healthcare events and information such as who did they see or talk to about their concern. Why, how and when the participant decided to act, or not act, on any changes
Symptom Experiences & Experiences of help-seeking	Importance of help-seeking	Any discussion or reference made on the relevant importance of help-seeking, especially in reference to the pandemic. Whether the pandemic has affected and/or changed how they think about the importance of help-seeking or rationalise it. Includes both medical and non-medical help-seeking
Fear of help-seeking & Experiences of help-seeking	Concerns about help-seeking	Any discussion or reference to the participant having concerns about help-seeking due to the pandemic. This includes healthcare appointments (primary and secondary care) and virtual and remote help-seeking avenues
Experiences of help-seeking	Experience of face-to-face and virtual/remote consultations	Any discussion or reference to participants experience of a face-to-face or virtual/remote healthcare consultation during the pandemic. Includes both primary and secondary healthcare consultations and views on the differences in help/advice/care compared to pre-pandemic
Experiences of help-seeking	Support and/or encouraging help-seeking and attending healthcare	Any discussion or reference to views/suggestions/ideas provided by participants to support and/or encourage help-seeking and attending healthcare (i.e. possible future facilitators)

Quinn-Scoggins HD, *et al. BMJ Open* 2022; 11:e053095. doi: 10.1136/bmjopen-2021-053095
