# Supplementary material for: Cancer symptom experience and help-seeking behaviour during the COVID-19 pandemic in the UK: a cross-sectional population survey
Source: BMJ Open. 2021 Sep 16;11(9):e053095. doi: 10.1136/bmjopen-2021-053095 (PMC8449845; doi:10.1136/bmjopen-2021-053095)
Supplement: Author's manuscript [file bmjopen-2021-053095.draft_revisions.pdf]

BMJ Open is committed to open peer review. As part of this commitment we make the peer review history of every article we publish publicly available.

When an article is published we post the peer reviewers' comments and the authors' responses online. We also post the versions of the paper that were used during peer review. These are the versions that the peer review comments apply to.

The versions of the paper that follow are the versions that were submitted during the peer review process. They are not the versions of record or the final published versions. They should not be cited or distributed as the published version of this manuscript.

BMJ Open is an open access journal and the full, final, typeset and author-corrected version of record of the manuscript is available on our site with no access controls, subscription charges or pay-per-view fees (http://bmjopen.bmj.com).

If you have any questions on BMJ Open's open peer review process please email info.bmjopen@bmj.com

BMJ Open

Cancer symptom experience and help-seeking behaviour during the COVID-19 pandemic in the United Kingdom: a cross-sectional population survey

Journal:	*BMJ Open*
Manuscript ID	bmjopen-2021-053095
Article Type:	Original research
Date Submitted by the Author:	05-May-2021
Complete List of Authors:	Quinn-Scoggins, H; Cardiff University, PRIME Centre Wales, Division of Population Medicine, School of Medicine<br>Cannings-John, Rebecca; Cardiff University Centre for Trials Research<br>Moriarty, Yvonne; Cardiff University, Centre for Trials Research<br>Whitelock, Victoria; Cancer Research UK, Cancer Intelligence<br>Whitaker, Katriina; University of Surrey, School of Health Sciences<br>Grozeva, Detelina; Cardiff University, Centre for Trials Research<br>Hughes, Jacqueline; Cardiff University, Centre for Trials Research<br>Townson, Julia; Cardiff University, Centre for Trials Research<br>Osborne, Kirstie; Cancer Research UK, Cancer Intelligence<br>Goddard, Mark; Cardiff University, Centre for Trials Research<br>McCutchan, GM; Cardiff University, Division of Population Medicine, School of Medicine<br>Waller, Jo; King's College London, School of Cancer and Pharmaceutical Sciences<br>Robling, Michael; Cardiff University, Centre for Trials Research; Cardiff University, DECIPHer (Centre for Development, Evaluation, Complexity and Implementation in Public Health Improvement), School of Social Sciences<br>Hepburn, Julie; Health and Care Research Wales Support Centre, Public Involvement Community<br>Moore, Graham; Cardiff University, DECIPHer (Centre for Development, Evaluation, Complexity and Implementation in Public Health Improvement), School of Social Sciences<br>Gjini, Ardiana; Public Health Wales; Cardiff University<br>Brain, Kate; Cardiff University, PRIME Centre Wales, Division of Population Medicine, School of Medicine
Keywords:	COVID-19, PREVENTIVE MEDICINE, PUBLIC HEALTH, STATISTICS & RESEARCH METHODS, ONCOLOGY, PRIMARY CARE

SCHOLARONE™
Manuscripts

I, the Submitting Author has the right to grant and does grant on behalf of all authors of the Work (as defined in the below author licence), an exclusive licence and/or a non-exclusive licence for contributions from authors who are: i) UK Crown employees; ii) where BMJ has agreed a CC-BY licence shall apply, and/or iii) in accordance with the terms applicable for US Federal Government officers or employees acting as part of their official duties; on a worldwide, perpetual, irrevocable, royalty-free basis to BMJ Publishing Group Ltd ("BMJ") its licensees and where the relevant Journal is co-owned by BMJ to the co-owners of the Journal, to publish the Work in this journal and any other BMJ products and to exploit all rights, as set out in our licence.

The Submitting Author accepts and understands that any supply made under these terms is made by BMJ to the Submitting Author unless you are acting as an employee on behalf of your employer or a postgraduate student of an affiliated institution which is paying any applicable article publishing charge ("APC") for Open Access articles. Where the Submitting Author wishes to make the Work available on an Open Access basis (and intends to pay the relevant APC), the terms of reuse of such Open Access shall be governed by a Creative Commons licence – details of these licences and which Creative Commons licence will apply to this Work are set out in our licence referred to above.

Other than as permitted in any relevant BMJ Author's Self Archiving Policies, I confirm this Work has not been accepted for publication elsewhere, is not being considered for publication elsewhere and does not duplicate material already published. I confirm all authors consent to publication of this Work and authorise the granting of this licence.

For peer review only

Cancer symptom experience and help-seeking behaviour during the COVID-19 pandemic in the United Kingdom: a cross-sectional population survey

Harriet Quinn-Scoggins*[1], Rebecca Cannings-John*[2], Yvonne Moriarty[2], Victoria Whitelock[3], Katriina L Whitaker[4], Detelina Grozeva[2], Jacqueline Hughes[2], Julia Townson[2], Kirstie Osborne[3], Mark Goddard[2], Grace McCutchan[5], Jo Waller[6], Michael Robling[2,7], Julie Hepburn[8], Graham Moore[7], Ardiana Gjini[9,10], Kate Brain[1]

*Dr Harriet Quinn-Scoggins and Dr Rebecca Cannings-John share equally first authorship of this manuscript

Corresponding author: Professor Kate Brain; Neuadd Meirionnydd, Heath Park, Cardiff, CF14 4YS, UK; BrainKE@Cardiff.ac.uk; ORCiD ID: 0000-0001-9296-9748

Author affiliations:

[1] PRIME Centre Wales, Division of Population Medicine, School of Medicine, Cardiff University, Neuadd Meirionnydd, Heath Park, Cardiff CF14 4YS, UK

[2] Centre for Trials Research, Cardiff University, Neuadd Meirionnydd, Heath Park, Cardiff CF14 4YS, UK

[3] Cancer Intelligence, Cancer Research UK, 2 Redman Place, London, E20 1JQ, UK

[4] School of Health Sciences, University of Surrey, Guildford, UK

[5] Wales Cancer Research Centre, Division of Population Medicine, School of Medicine, Cardiff University, Neuadd Meirionnydd, Heath Park, Cardiff CF14 4YS, UK

[6] Cancer Prevention Group, School of Cancer and Pharmaceutical Sciences, King's College London, Great Maze Pond, London SE1 9RT, UK

[7] DECIPHer (Centre for Development, Evaluation, Complexity and Implementation in Public Health Improvement), School of Social Sciences, Cardiff University, 1-3 Museum Place, Cardiff UK

[8] Public Involvement Community, Health and Care Research Wales Support Centre, Castlebridge 4, 15-19, Cowbridge Road East, Cardiff CF11 9AB, UK

[9] Public Health Wales, 2 Capital Quarter, Tyndall Street, Cardiff CF10 4BZ, UK

[10] Cardiff University, Neuadd Meirionnydd, Heath Park, Cardiff CF14 4YS, UK

Key words: COVID-19; Preventive medicine; Public Health; Statistics & Research Methods; Oncology; Primary Care

Word count: (3998/4000 words)

For peer review only - http://bmjopen.bmj.com/site/about/guidelines.xhtml

ABSTRACT (272/300)

Objectives: To understand self-reported potential cancer symptom help-seeking behaviours and attitudes during the first 6 months (March – August 2020) of the UK COVID-19 pandemic.

Design: UK population-based survey conducted during August and September 2020. Correlates of help-seeking behaviour were modelled using logistic regression in participants reporting potential cancer symptoms during the previous six months. Qualitative telephone interviews with a purposeful subsample of participants, analysed thematically.

Setting: Online UK wide survey.

Participants: 7,543 adults recruited via Cancer Research UK online panel provider (Dynata) and HealthWise Wales (a national register of 'research ready' participants) supplemented with social media (Facebook and Twitter) recruitment. 30 participants were also interviewed.

Main outcome measures: Survey measures included experiences of 15 potential cancer symptoms, help-seeking behaviour, barriers and enablers to help-seeking.

Results: Of 3,025 (40.1%) participants who experienced a potential cancer symptom, 44.8% (1,355/3,025) had not contacted their GP. Odds of help-seeking were higher among participants with disability (odds ratio (aOR)=1.38, 95% CI 1.11-1.71 adjusted) and who experienced more symptoms (aOR=1.68, 95% CI 1.56-1.82) and lower among those who perceived COVID-19 as the cause of symptom(s) (aOR=0.36, 95% CI 0.25-0.52). Barriers included worries about wasting the doctor's time (15.4%), putting strain on healthcare services (12.6%) and not wanting to make a fuss (12.0%). Interviewees reported reluctance to contact the GP due to concerns about COVID-19 and fear of attending hospitals, and described putting their health concerns on hold.

Conclusions: Many people avoided healthcare services despite experiencing potential cancer symptoms during the COVID-19 pandemic. Alongside current help-seeking campaigns, well-timed and appropriate nationally co-ordinated campaigns should signal that services are open safely for those with unusual or persistent symptoms.

Registration: ISRCTN17782018

For peer review only

ARTICLE SUMMARY:

Strengths and Limitations of this study:

- To our knowledge this is the first UK population survey of the impact of COVID-19 on help-seeking for potential cancer symptoms.
- A large sample was recruited across two online surveys and data pooled where applicable, providing a larger dataset for analysis which was broadly representative of the UK population.
- Data collection occurs between August and September 2020 and reflects on the first lockdown period in the UK.
- We assessed self-report of actual symptoms experienced during the first 6 months of the pandemic, reducing the known biases associated with retrospective recall of symptoms in patient samples or anticipated responses to hypothetical symptoms in community samples.
- Survey data were supplemented with in-depth qualitative interviews, providing rich insight and context regarding symptom help-seeking behaviour during the pandemic.

For peer review only

For peer review only - http://bmjopen.bmj.com/site/about/guidelines.xhtml

BACKGROUND:

Cancer is the leading cause of mortality in the United Kingdom (UK)[1] and globally.[2] In countries with a 'gatekeeper' healthcare system such as the UK, most cancers are diagnosed symptomatically through primary care.[3] Diagnosing symptomatic cancer earlier can enable more timely treatment with better clinical outcomes across a range of cancers.[4,5] However, this route to cancer early diagnosis has been severely disrupted during the COVID-19 pandemic. Around 350,000 fewer people were on an urgent General Practitioner (GP) suspected cancer referral route in March-November 2020 in England alone, a reduction of 19% compared to the same time during the previous year.[6] Early evidence from the MAINROUTE study[7] of primary care consultations during the COVID-19 pandemic suggests that people were not coming forward with potential cancer symptoms (personal communication, Nicholson B. 2021). This has led to concerns that members of the public may not be seeking help from their GP due to factors including fear of coronavirus infection and concerns about placing additional burden on the National Health Service (NHS).[8]

During the first UK lockdown from March 2020, the UK government message to *"stay home, protect the NHS, save lives"* was intended to control the spread of COVID-19, but potentially sent a strong signal to the public that cancer can wait.[9] Consequently, the pandemic is likely to have affected key stages across the cancer diagnostic pathway[10] including the patient interval.[11] As set out in the Model of Pathways to Treatment,[12] the patient interval combines the time between a person noticing a bodily change or symptom to perceiving a reason to seek medical help (the appraisal interval), and the time between perceiving a reason to seek medical help to first contact with a medical professional (the help-seeking interval). In UK studies conducted before the COVID-19 pandemic, rates of self-reported symptom help-seeking in adults aged over 50 years ranged from 26.5% seeking help from their GP for at least one potential cancer symptom over a one-month period,[13] to 60% over twelve months[13] and 67% over three months.[14] Adverse impact of the pandemic on people's willingness to seek help for potential cancer symptoms seems likely, especially for non-specific or respiratory symptoms that are similar to COVID-19 symptoms such as a persistent or changing cough, fatigue and breathlessness. Evidence from pre-COVID studies suggests that non-specific symptoms such as those previously mentioned may be overlooked or dismissed,[15] in part due to worry about wasting the doctor's time.[16] In adults with existing lung and cardiac comorbid conditions, potential cancer symptoms may be misattributed and not acted on.[17] Fear of COVID-19 infection may deter attendance in healthcare settings, especially among high risk and shielding groups.[18] Changes to healthcare service delivery during the pandemic, including remote GP consultations, may create additional barriers to accessing services.[19]

For peer review only - http://bmjopen.bmj.com/site/about/guidelines.xhtml

Evidence is needed regarding public perceptions of potential cancer symptoms and symptom help-seeking behaviour, and potential inequalities in help-seeking, to understand the factors driving reduced GP consultations in the UK during COVID-19. We conducted a large-scale population survey informed by relevant theory[12,20] to understand self-reported symptom help-seeking attitudes and behaviour during the pandemic. Anticipating that the UK adult cohort would be more reluctant to seek help for symptoms than before the pandemic, we compared the overall proportion seeking help during the first pandemic wave with UK pre-pandemic data recently reported in the USEFUL study.[13]

METHODS:

study design

A prospective, mixed-methods observational cohort study in the UK population during the COVID-19 pandemic. The study protocol and analysis plans were pre-registered on Open Science Framework.[21] Findings are reported in accordance with the STROBE guidelines for surveys and observational studies.[22,23]

survey participants and procedures

Two cross-sectional online surveys were conducted in parallel, the COVID-19 Health and Help-Seeking Behaviour Study (CABS) and the Cancer Research UK (CRUK) COVID-19 Cancer Awareness Measure (COVID-CAM). COVID-CAM was based on CRUK's Cancer Awareness Measure 2019.[24,25] Key measures were aligned across the two surveys and data pooled where appropriate. Eligible participants were age 18 years or over (due to collecting additional survey data on attitudes and behaviours relating to cancer prevention and cervical screening), resident in the UK and able to speak English. Data were collected between 6th August and 18th September 2020, after the first UK lockdown which started on 23rd March 2020. Study information was available online prior to participants providing electronic informed consent online.

Participants were recruited to the CABS survey via HealthWise Wales (HWW, a national register of 'research ready' participants)[26] and social media (Facebook and Twitter). Potentially under-represented groups including men, smokers, black, Asian and minority ethnic groups, and people living in socioeconomically deprived areas were targeted by HWW using personalised emails and Facebook-targeted advertising. Participants were recruited to the COVID-CAM survey via Dynata, an online panel provider (www.dynata.com). Quotas were placed on age, gender, social grade and UK region to recruit a

For peer review only

For peer review only - http://bmjopen.bmj.com/site/about/guidelines.xhtml

nationally representative sample and sample size for ethnic minority groups was increased (relative to UK population statistics) to increase representation.

survey measures

Details of survey measures included in CABS and COVID-CAM are in **Supplementary file Table S1**. Questions were asked over the previous six months. Two attention check questions were included in both surveys.[27]

qualitative interviews

Survey participants who consented to interview were purposively sampled from the CABS study cohort according to age, gender and symptom experience. Consent for interview and audio-recording was reconfirmed verbally. A semi-structured topic guide was used to explore participants' views on attending primary and secondary healthcare in light of the COVID-19 pandemic, contextual influences on help-seeking and strategies to encourage future help-seeking **(Supplementary file S2 - Interview topic guide).** Interview participants were reimbursed with a £20 voucher. Transcribed anonymised data were thematically analysed[28] using NVivo12 (QSR international), with 20% of transcriptions dual coded.

sample size

The study was powered to examine the correlates of longer help-seeking interval (primary outcome) in those who experienced one or more potential cancer symptoms.[21] Due to the urgency of early findings presented in this paper, the primary outcome analysis will be reported in a subsequent paper; the current modelling of help-seeking behaviour is therefore exploratory.

statistical analysis

Analyses were conducted using SPSS 25.0 and Stata 16.0. Data were weighted to match the UK population profile on age, gender, ethnicity and country (i.e. devolved nation) using English 2011 Census and Office for National Statistics mid-year estimates. Cases with missing data were excluded on a per-analysis basis. Descriptive analyses were used to identify sample characteristics, prevalence of potential cancer symptoms, help-seeking barriers and enablers and, among those who had experienced potential cancer symptoms, symptom perceptions and help-seeking behaviour. The method of categorising symptom help-seeking was based on the USEFUL study of symptom help-seeking behaviour.[13] Outcomes were dichotomised as 'contact with GP in last 6 months' versus 'no contact' for individual symptoms. For the composite outcome across all symptoms, the outcome was 'contact for at least one symptom in the last 6 months' versus 'no contact for any symptoms'. Sample characteristics

For peer review only - http://bmjopen.bmj.com/site/about/guidelines.xhtml

and symptom prevalence are presented unweighted and weighted. Due to similar estimates, subsequent analyses are presented as unweighted.

correlates of symptom help-seeking behaviour

Descriptive summary statistics and logistic regression models were used to estimate the prevalence and odds respectively of GP help-seeking in those who had experienced at least one symptom (compared to not seeking help for any of their symptoms). The following key factors were examined: country, gender, age group, ethnicity, marital relationship, education, smoking status, region, disability, cancer status (self, family and friends), perceived symptom causes, barriers towards medical help-seeking, enablers towards medical help-seeking (COVID-CAM only) and cancer symptom recognition. We additionally fitted multivariable regression models to explore the independent contribution of potential factors by including all factors as independent variables to account for potential confounding of crude associations by other variables. The study was designed to fit descriptive models, capturing the association between dependent and independent variables, rather than for prediction or causality. Multi-collinearity between factors was assessed using the Variance Inflation Factor (VIF) (VIF >4 warrants further investigation). Data are reported as crude and adjusted odds ratios (ORs) with 95% confidence intervals (CIs).

patient and public involvement

Patient and public involvement (PPI) was included at all stages from conceptualisation through to data interpretation. Working with CRUK's Cancer Insights Panel, the Wales Centre for Primary and Emergency Care Research PPI Group (Service Users for Primary and Emergency Care Research Group) and our study PPI co-applicant (JH), all public-facing materials including study information, consent procedures, survey and interview topic guides were reviewed and amended as appropriate. New COVID-specific survey items were tested for acceptability prior to inclusion in the survey.

RESULTS:

characteristics of participants

A total of 8,167 participants responded to the survey in August and September, of whom 7,543 (92.4%) were included **(Figure 1)**. Demographic characteristics of the pooled sample (n=7,543) and by recruitment route are shown in **Table 1**. Almost half the unweighted pooled sample was age 55 years and over (47.4%) and female (49.2%). Most were of White ethnic background (88.6%) and living in England (65.0%). Over one third had university level education or higher (38.3%). Current smokers and former smokers comprised 18.8% and 32.3% of the sample, respectively. Under a fifth (17.4%) considered themselves disabled and 8.7% had experienced cancer themselves.

For peer review only - http://bmjopen.bmj.com/site/about/guidelines.xhtml

Table 1: Sample characteristics, UK, August -September 2020

Data are n (%) and unweighted unless otherwise stated

	Pooled sample N=7,543	Pooled sample weighted[1] N=7,543	CABS N=1,876	COVID-CAM N=5,667
**Age (years)**				
18-24	543 (7.2)	665 (8.8)	12 (0.6)	531 (9.4)
25-34	945 (12.5)	1,345 (17.8)	53 (2.8)	892 (15.7)
35-44	1,149 (15.2)	1,420 (18.8)	132 (7.0)	1,017 (17.9)
45-54	1,221 (16.2)	1,420 (18.8)	202 (10.8)	1,019 (18.0)
55-64	1,282 (17.0)	1,194 (15.8)	417 (22.2)	865 (15.3)
65-74	1,795 (23.8)	816 (10.8)	738 (39.3)	1,057 (18.7)
75+	497 (6.6)	590 (7.8)	271 (14.4)	226 (4.0)
Missing/other/prefer not to say	111 (1.5)	93 (1.2)	51 (2.7)	60 (1.1)
**Gender**				
Male	3,807 (50.5)	3,681 (48.8)	1,044 (55.7)	2,763 (48.8)
Female	3,709 (49.2)	3,832 (50.8)	827 (44.1)	2,882 (50.9)
Non-binary, transgender female or other	27 (0.4)	29 (0.4)	5 (0.3)	22 (0.4)
**Ethnicity**				
White	6,685 (88.6)	6,948 (92.1)	1,821 (97.1)	4,864 (85.8)
Mixed/Multiple ethnic groups	143 (1.9)	153 (2.0)	19 (1.0)	124 (2.2)
Asian/Asian British	458 (6.1)	274 (3.6)	15 (0.8)	443 (7.8)
Black/African/Caribbean/Black British	154 (2.0)	135 (1.8)	14 (0.7)	150 (2.6)
Other ethnic group	96 (1.3)	26 (0.3)		86 (1.5)
Prefer not to say	7 (0.1)	8 (0.1)	7 (0.4)	NA
**Country/Region**				
England	4,904 (65.0)	6,311 (83.7)	76 (4.1)	4,828 (85.2)
Wales	2,045 (27.1)	376 (5.0)	1,797 (95.8)	248 (4.4)
Scotland	456 (6.0)	601 (8.0)	19 (1.0)	453 (8.0)
Northern Ireland	105 (1.4)	225 (3.0)		105 (1.9)
England:				
North East England	265 (3.5)	376 (5.0)		265 (4.7)
North West England	621 (8.2)	826 (11.0)		618 (10.9)
Yorkshire and Humberside	479 (6.4)	526 (7.0)		476 (8.4)
East Midlands	417 (5.5)	601 (8.0)		415 (7.3)
East Anglia	503 (6.7)	676 (9.0)		500 (8.8)
West Midlands	513 (6.8)	676 (9.0)		508 (9.0)
South East England	830 (11.0)	1,052 (13.9)	24 (1.3)	806 (14.2)
South West England	473 (6.3)	601 (8.0)	9 (0.5)	464 (8.2)
London	803 (10.6)	977 (12.9)	27 (1.4)	776 (13.7)
Prefer not to say	33 (0.4)	30 (0.4)	0 (0.0)	33 (0.6)
**Highest level of education**				
Degree or higher degree	2,892 (38.3)	2,713 (36.0)	897 (47.8)	1,995 (35.2)
A levels or further education	2,447 (32.4)	2,537 (33.7)	542 (28.9)	1,905 (33.6)
O levels/GCSEs	1,565 (20.7)	1,694 (22.5)	268 (14.3)	1,297 (22.9)

For peer review only

For peer review only - http://bmjopen.bmj.com/site/about/guidelines.xhtml

No formal qualifications	412 (5.5)	390 (5.2)	105 (9.6)	307 (5.4)
Still studying	81 (1.1)	87 (1.2)	9 (0.5)	72 (1.3)
Prefer not to say	74 (1.0)	65 (0.9)	26 (1.4)	48 (0.8)
Other	72 (1.0)	55 (0.7)	29 (1.6)	43 (0.8)
**Smoking status**				
Never smoked	3,586 (47.5)	3,601 (47.7)	842 (45.9)	2,744 (48.4)
Former smoker	2,435 (32.3)	2,157 (28.6)	839 (44.7)	1,596 (28.2)
Current smoker	1,417 (18.8)	1,706 (22.6)	150 (8.0)	1,267 (22.4)
Other/prefer not to say	105 (1.4)	79 (1.0)	45 (2.3)	60 (1.1)
**Do you consider yourself to have a disability?**				
No	6,079 (82.6)	6,136 (83.4)	1,445 (78·7)	4,634 (83.8)
Yes	1,284 (17.4)	1,223 (16.6)	390 (21·3)	894 (16.2)
**Have you, anyone in your family or any of your friends had cancer?**				
No	1,745 (23.1)	2,000 (26.5)	157 (8.3)	1,558 (28.0)
Yes, other (family and friends)[2]	5,141 (68.2)	5029 (66.7)	1,460 (77.8)	3,681 (65.0)
Yes, self	657 (8.7)	512 (6.8)	259 (14.9)	398 (7.0)

Abbrev: CABS = COVID-19 Health and Help-Seeking Behaviour Study cohort recruited via HealthWise Wales and social media; COVID-CAM = Cancer Research UK's COVID-19 Cancer Awareness Measure sample recruited via Dynata, an online panel provider; NA = Not available as an option.
[1] All data are weighted to match the UK adult population on age, gender, ethnicity and country.
[2] Participants stated that cancer was experienced in friends and family only and not in self.

symptom prevalence

During the past six months, 40.1% (3,025/7,543) of survey participants had experienced at least one potential cancer symptom **(Table 2)**. Of these, a median of two symptoms per participant were reported (range 1-15 symptoms), while 31.8% (961) experienced three or more symptoms. Nearly one third of all participants had experienced at least one non-specific symptom (30.3%), almost a fifth reported at least one red flag symptom (17.6%), and at least one symptom possibly indicative of lung cancer (18.4%). The prevalence of individual symptoms ranged from 21.3% ('tired all the time') to 1.5% ('coughing up blood'). Among those reporting that they were 'tired all the time', had 'a persistent cough' or 'shortness of breath', around half said the symptom pre-dated the pandemic (51.5%, 49.3% and 48.5% respectively) **(Supplementary file Table S3).**

For peer review only

For peer review only - http://bmjopen.bmj.com/site/about/guidelines.xhtml

Table 2: Participants experiencing potential cancer symptoms and associated symptom help-seeking, during March to August 2020

Data are n (%) and unweighted unless otherwise stated

Potential cancer symptom	Had symptom[1]	Had symptom - weighted[2]	Did not contact GP in the last 6 months[3]	Did not contact GP in the last 12 months - USEFUL Study[4]	Contacted GP in the last 6 months[5]	Contacted GP in the last 12 months - USEFUL Study[4]
	**n / 7,543 (%)**	**n / 7,543 (%)**	**n /S (%)**	**n (%)**	**n /S (%)**	**n (%)**
**Non-specific symptom**						
A persistent change in bowel habits	541 (7.2)	525 (7.0)	267 (49.4)	682/1,323 (51.5)	254 (47.0)	641/1,323 (48.5)
A persistent change in bladder habits	450 (6.0)	414 (5.5)	216 (48.0)	-	227 (50.4)	-
Tired all the time	1,603 (21.3)	1,614 (21.4)	1,031 (64.3)	1,778/3,078 (57.8)	540 (33.7)	1,300/3,078 (42.2)
Persistent unexplained pain	662 (8.8)	646 (8.6)	286 (43.2)	-	361 (54.5)	-
**Non-specific/Red flag symptom**						
Unexplained weight loss	395 (5.2)	433 (5.7)	205 (51.9)	152/341 (44.6)	179 (45.3)	189/341 (55.4)
**Red flag symptom**						
A change in the appearance of a mole	391 (5.2)	402 (5.3)	229 (58.6)	-	157 (40.2)	-
An unexplained lump or swelling	422 (5.6)	418 (5.5)	173 (41.0)	-	239 (56.6)	-
Unexplained bleeding	267 (3.5)	291 (3.9)	115 (43.1)	-	143 (53.6)	-
A persistent difficulty swallowing	237 (3.1)	248 (3.3)	97 (40.9)	557/884 (63.0)	128 (54.0)	327/884 (37.0)
A sore that does not heal	291 (3.9)	297 (3.9)	146 (50.2)	-	128 (44.0)	-
**Non-specific/*Lung-specific symptom***						
Coughing up blood	114 (1.5)	127 (1.7)	35 (30.7)	31/91 (34.1)	67 (58.8)	60/91 (65.9)
**Lung-specific symptom**						
Shortness of breath	1,052 (13.9)	966 (12.8)	538 (51.1)	1,228/2,647 (46.4)	484 (46.0)	1,419/2,647 (53.6)
Persistent hoarseness	200 (2.7)	206 (2.7)	95 (47.5)	941/1,319 (71.3)	96 (48.0)	378/1,319 (28.7)
A persistent cough	444 (5.9)	401 (5.3)	209 (47.1)	1,088/2,189 (49.7)	230 (51.8)	1,101/2,189 (50.3)
A change in an existing cough	196 (2.6)	219 (2.9)	84 (42.9)	153/298 (51.3)	100 (51.0)	145/298 (48.7)
All potential cancer symptoms	***3,025 (40.1)[6]***	***2,909 (38.6)***	***1,355/3,025 (44.8)[7]***	***3,974/9,810 (40.5)***	***1,636/3,025 (54.1)[8]***	***5,836/9,810 (59.5)***
Non-specific symptom	***2,284 (30.3)[6]***	***2,261 (30.0)***				
Red flag symptom	***1,327 (17.6)[6]***	***1,310 (17.4)***				
Lung-specific symptom	***1,386 (18.4)[6]***	***1,289 (17.1)***				

n=number, n/S = number of respondents representing each symptom help-seeking behaviour/number of respondents who had this symptom.

[1] Denominator includes those who did not have a symptom and those who preferred not to say (around 1% of the sample). [2] All data are weighted to match the UK adult population on age, gender, ethnicity and country. [3] Includes participants who had not contacted the GP yet, but planned to. 'Did not contact GP' and 'Contacted GP' columns are mutually exclusive. Denominator includes participants who preferred not to say. [4] Comparator data for adults aged >50 years who did and did not contact the GP in the last 12

For peer review only

For peer review only - http://bmjopen.bmj.com/site/about/guidelines.xhtml

months (Hannaford et al., 2020). [5] A further breakdown of help-seeking intervals is in **Supplementary file Table S6.** [6] At least one potential cancer symptom reported. [7] Did not contact the GP for symptoms reported in the last 6 months. 'Did not contact GP' and 'Contacted GP' columns are mutually exclusive. Denominator also includes 34 (1.1%) who preferred not to say across all their symptoms. [8] Contacted the GP for at least one symptom in the la

For peer review only

help-seeking barriers and enablers

The most frequently endorsed barriers to medical help-seeking in the overall sample were worry about wasting the healthcare professional's time (15.4%), worry about putting extra strain on the NHS (12.6%), not wanting to be seen as someone who makes a fuss (12.0%), difficulty getting an appointment with a particular healthcare professional (10.3%) and worry about catching coronavirus (9.6%). Remote consulting was one of the least frequently endorsed barriers (4.8%) **(Supplementary file Table S4).** A median of one barrier (25th to 75th centile 1 to 2 barriers, range 0 to 14) was identified per participant.

Of the overall sample (n=7,543), around two thirds reported feeling safe from COVID-19 if they needed to attend an appointment at their GP practice (5,142/7,543, 68.2%) or hospital (4,613/7,543, 61.2%). Nearly three quarters (5,452/7,543, 72.3%) were worried about delays to cancer tests and investigations due to COVID-19.

For COVID-CAM survey participants (n=5,667) the main enablers to speaking to a medical professional were having a symptom that was bothersome (17.7%), that didn't go away (17.1%), was painful (14.4%) and unusual (12.5%), and having a feeling that something wasn't right (13.0%) **(Supplementary file Table S5).**

symptom help-seeking behaviour

Among 3,025 participants who experienced at least one potential cancer symptom, 44.8% had not contacted the GP for any of their reported symptoms over a 6-month time frame, whereas 40.5% had not contacted their GP over a 12-month time frame in the USEFUL study **(Table 2)**. A small proportion preferred not to say across all symptoms (1.1%). The proportion of participants not seeking help varied by symptom. A substantial proportion of participants had not sought help for red flag symptoms including coughing up blood (30.7%), an unexplained lump or swelling (41.0%) or a change in the appearance of a mole (58.6%). Almost half of those who reported non-specific symptoms including 'a persistent change in bowel habits' (49.4%) and 'a persistent change in bladder habits' (48.0%) had not sought help from their GP, whilst a higher proportion (64.3%) reporting being 'tired all the time' had not sought help. Around half of those experiencing lung-specific symptoms such as 'a persistent cough' (47.1%) and 'shortness of breath' (51.1%) had not sought help. A further breakdown of help-seeking according to recommended intervals is provided in **Supplementary file Table S6.**

For peer review only - http://bmjopen.bmj.com/site/about/guidelines.xhtml

As shown in **Table 2**, the proportion of participants who had not contacted their GP over a 6-month time frame appeared to be higher than USEFUL study data for individual symptoms over a 12-month time frame including ‘tired all the time’ (64.3% in the current study versus 57.8% in the USEFUL study), ‘unexplained weight loss’ (51.9% versus 44.6%) and to a lesser extent ‘shortness of breath’ (51.1% versus 46.4%). Proportions not seeking help for ‘persistent change in bowel habits’ (49.4% versus 51.5%) and ‘persistent cough’ (47.1% versus 49.7%) appeared comparable. The proportion of participants who had not contacted their GP in the current study appeared to be lower than USEFUL study data for ‘persistent difficulty swallowing’ (40.9% versus 63.0%), ‘persistent hoarseness’ (47.5% versus 71.3%), ‘change in an existing cough’ (42.9% versus 51.3%) and to a lesser extent ‘coughing up blood’ (30.7% versus 34.1%). It should be noted that relatively small numbers of participants in the current study reported experiencing the latter four symptoms.

correlates of symptom help-seeking behaviour

In unadjusted analyses, seeking help from the GP for at least one symptom was associated with former or current smoking, disability, experience of cancer (self), perceiving cancer as the cause of symptom(s) experienced, and reporting a greater number of potential cancer symptoms **(Table 3).** Perceiving COVID-19 as the cause of symptom(s) was associated with lower odds of help-seeking. There were no other statistically significant unadjusted associations. After adjustment for other factors, disability, reporting more symptoms and not perceiving COVID-19 as the cause of symptom(s) experienced remained statistically significantly associated with help-seeking.

For peer review only

For peer review only - http://bmjopen.bmj.com/site/about/guidelines.xhtml

Table 3: Unadjusted and adjusted logistic regression models predicting self-reported symptom help-seeking in participants who experienced at least one potential cancer symptom, UK, August-September 2020 (n=3,025[1]). *Data are n (%) and unweighted unless otherwise stated*

	Did not contact GP[2] n=1,355	Contacted GP[2] n=1,636	Crude OR (95% CI)	Adjusted OR (95% CI) N=2,281
**Age (years) N=2,942**				
18-24	127 (46.4)	147 (53.6)	ref (1.0)	ref (1.0)
25-34	199 (49.7)	201 (50.3)	0.87 (0.64 to 1.19)	0.80 (0.52 to 1.24)
35-44	196 (45.7)	233 (54.3)	1.03 (0.76 to 1.39)	1.12 (0.73 to 1.71)
45-54	211 (47.1)	237 (52.9)	0.97 (0.72 to 1.31)	1.11 (0.72 to 1.70)
55-64	220 (45.2)	267 (54.8)	1.05 (0.78 to 1.41)	1.08 (0.70 to 1.67)
65-74	284 (41.8)	396 (58.2)	1.20 (0.91 to 1.60)	1.29 (0.83 to 2.00)
75+	97 (43.3)	127 (56.7)	1.13 (0.79 to 1.61)	1.20 (0.72 to 2.00)
p-value			0.261	0.321
**Gender N=2,978**				
Male	625 (43.7)	804 (56.3)	ref (1.0)	ref (1.0)
Female	727 (46.9)	822 (53.1)	0.88 (0.76 to 1.02)	0.99 (0.82 to 1.21)
p-value			0.080	0.951
**Ethnicity N=2,988**				
White	1,193 (45.0)	1,457 (55.0)	ref (1.0)	ref (1.0)
Ethnic minorities[3]	160 (47.3)	178 (52.7)	0.91 (0.73 to 1.14)	0.85 (0.61 to 1.18)
p-value			0.420	0.328
**Country N=2,971**				
England	854 (47.2)	955 (52.8)	ref (1.0)	ref (1.0)
Wales	405 (42.5)	549 (57.5)	1.21 (1.03 to 1.42)	1.23 (0.98 to 1.54)
Scotland	72 (43.1)	95 (56.9)	1.18 (0.86 to 1.62)	1.35 (0.90 to 2.02)
Northern Ireland	15 (36.6)	26 (63.4)	1.55 (0.82 to 2.95)	1.79 (0.62 to 5.20)
p-value			0.062	0.140
**Country/Region[4] N=2,971**				
Wales	405 (42.5)	549 (57.5)	ref (1.0)	
Scotland	72 (43.1)	95 (56.9)	0.97 (0.70 to 1.36)	
Northern Ireland	15 (36.6)	26 (63.4)	1·28 (0.67 to 2.55)	

For peer review only

	Did not contact GP[2] n=1,355	Contacted GP[2] n=1,636	Crude OR (95% CI)	Adjusted OR (95% CI) N=2,281
England:				
North East England	59 (53.6)	51 (46.4)	0.64 (0.43 to 0.95)	
North West England	109 (45.2)	132 (54.8)	0.89 (0.67 to 1.19)	
Yorkshire and Humberside	85 (47.0)	96 (53.0)	0.83 (0.61 to 1.15)	
East Midlands	72 (50.3)	71 (49.7)	0.73 (0.51 to 1.03)	
South East England	130 (45.8)	154 (54.2)	0.87 (0.67 to 1.14)	
East Anglia	72 (43.1)	95 (56.9)	0.97 (0.70 to 1.36)	
South West England	84 (48.0)	91 (52.0)	0.80 (0.58 to 1.10)	
West Midlands	96 (46.6)	110 (53.4)	0.85 (0.62 to 1.14)	
London	147 (48.7)	155 (51.3)	0.78 (0.60 to 1.01)	
p-value			0.379	
**Highest level of education N=2,934**				
Degree or higher degree	514 (47.2)	574 (52.8)	ref (1.0)	ref (1.0)
A-levels or further education	460 (46.2)	536 (53.8)	1.04 (0.88 to 1.24)	0.91 (0.73 to 1.14)
O levels/GCSEs	265 (42.3)	362 (57.7)	1.22 (1.00 to 1.49)	1.07 (0.83 to 1.39)
Still studying	16 (36.4)	28 (63.6)	1.57 (0.84 to 2.93)	1.21 (0.51 to 2.89)
No formal qualifications	73 (40.8)	106 (59.2)	1.30 (0.94 to 1.79)	0.77 (0.51 to 1.16)
p-value			0.127	0.494
**Smoking status N=2,948**				
Never smoked	595 (50.6)	580 (49.4)	ref (1.0)	ref (1.0)
Former smoker	436 (41.2)	623 (58.8)	1.47 (1.24 to 1.73)	1.16 (0.94 to 1.44)
Current smoker	302 (42.3)	412 (57.7)	1.40 (1.16 to 1.69)	1.03 (0.80 to 1.32)
p-value			<0.001	0.358
**Relationship status N=2,976**				
Not married or cohabiting	516 (46.4)	597 (53.6)	ref (1.0)	ref (1.0)
Married or cohabiting	831 (44.6)	1,032 (55.4)	1.07 (0.92 to 1.25)	0.95 (0.78 to 1.16)
p-value			0.352	0.647
**Do you consider yourself to have a disability? N=2,900**				
No	1,042 (50.7)	1,014 (49.3)	ref (1.0)	ref (1.0)
Yes	281 (33.3)	563 (66.7)	2.06 (1.74 to 2.43)	1.38 (1.11 to 1.71)

For peer review only - http://bmjopen.bmj.com/site/about/guidelines.xhtml

For peer review only

	Did not contact GP[2] n=1,355	Contacted GP[2] n=1,636	Crude OR (95% CI)	Adjusted OR (95% CI) N=2,281
p-value			<0.001	0.003
**Have you, anyone in your family or any of your friends had cancer? N=2,991**				
No	270 (50.3)	267 (49.7)	ref (1.0)	ref (1.0)
Yes, other (family and friends)[5]	974 (45.8)	1,154 (54.2)	1.20 (0.99 to 1.45)	1.09 (0.84 to 1.43)
Yes, self	111 (34.0)	215 (66.0)	1.96 (1.47 to 2.60)	1.12 (0.76 to 1.66)
p-value			<0.001	0.783
**Symptom attributed to cancer[6] N=2,990**				
Not cancer	1,342 (45.6)	1,601 (54.4)	ref (1.0)	ref (1.0)
Cancer	12 (25.5)	35 (74.5)	2.44 (1.26 to 4.73)	1.30 (0.56 to 3.04)
p-value			0.008	0.547
**Symptom attributed to COVID[6] N=2,990**				
Not COVID	1,214 (44.0)	1,547 (56.0)	ref (1.0)	ref (1.0)
COVID	140 (61.1)	89 (38.9)	0.50 (0.38 to 0.66)	0.36 (0.25 to 0.52)
p-value			<0.001	<0.001
**Number of barriers to help-seeking reported (0 to 17) N=2,991** Median (25th to 75th centiles)	1 (1 to 3)	1 (1 to 3)	1.04 (1.00 to 1.08)	0.97 (0.92 to 1.03)
p-value			0.057	0.315
**Confident that I would be safe from coronavirus if I needed to attend an appointment at a hospital N=2,645**				
Strongly agree	251 (44.7)	311 (55.3)	ref (1.0)	ref (1.0)
Somewhat agree	518 (44.3)	650 (55.7)	1.01 (0.83 to 1.24)	0.86 (0.64 to 1.15)
Somewhat disagree	268 (44.0)	341 (56.0)	1.03 (0.82 to 1.29)	0.74 (0.51 to 1.06)
Strongly disagree	149 (48.7)	157 (51.3)	0.85 (0.64 to 1.12)	0.58 (0.36 to 0.94)
p-value			0.547	0.150
**Confident that I would be safe from coronavirus if I needed to attend an appointment at my GP surgery N=2,692**				
Strongly agree	337 (47.3)	375 (52.7)	ref (1.0)	ref (1.0)

For peer review only - http://bmjopen.bmj.com/site/about/guidelines.xhtml

	Did not contact GP[2] n=1,355	Contacted GP[2] n=1,636	Crude OR (95% CI)	Adjusted OR (95% CI) N=2,281
Somewhat agree	545 (44.1)	690 (55.9)	1.14 (0.95 to 1.37)	1.21 (0.92 to 1.58)
Somewhat disagree	217 (41.1)	311 (58.9)	**1.29 (1.03 to 1.62)**	1.47 (1.02 to 2.12)
Strongly disagree	102 (47.0)	115 (53.0)	1.01 (0.75 to 1.37)	0.93 (0.55 to 1.56)
p-value			0.146	0.082
**Worried about delays to cancer tests and investigations caused by coronavirus N=2,720**				
Strongly agree	479 (43.8)	614 (56.2)	ref (1.0)	ref (1.0)
Somewhat agree	534 (46.2)	621 (53.8)	0.91 (0.77 to 1.07)	1.03 (0.83 to 1.25)
Somewhat disagree	126 (41.0)	181 (59.0)	1.12 (0.87 to 1.45)	1.18 (0.86 to 1.62)
Strongly disagree	77 (46.7)	88 (53.3)	0.89 (0.64 to 1.24)	0.97 (0.65 to 1.45)
p-value			0.340	0.762
**Cancer symptom recognition score (score 0 to 15) N=2,991** Median (25th to 75th centiles)	11 (8 to 14)	11 (8 to 14)	1.00 (0.99 to 1.02)	1.01 (0.99 to 1.04)
p-value			0.789	0.263
**Number of symptoms (maximum 15) N=2,991** Median (25th to 75th centiles)	1 (1 to 2)	2 (1 to 4)	1.62 (1.53 to 1.72)	1.68 (1.56 to 1.82)
p-value			<0.001	<0.001

OR = odds ratio; CI = confidence interval; Note: an odds ratio >1 indicates increased odds of help-seeking. [1] n=34 participants indicated that they prefer not to say across all symptoms and were excluded from the analysis. [2] Contacted the GP within six months. [3] Ethnicity groups combined for analysis due to small numbers: 'Mixed/multiple ethnic groups', 'Asian/Asian British', 'Black/African/Caribbean/Black British', 'Other ethnic group', 'Prefer not say'. [4] Not included in multivariable model due to collinearity with country.
[5] Participants stated that cancer was experienced in friends and family only and not in self. [6] Perceived causes for each of eight symptoms (unexplained lump or swelling, persistent cough, unexplained bleeding, persistent hoarseness, coughing up blood, tired all the time, change in existing cough, shortness of breath)

For peer review only - http://bmjopen.bmj.com/site/about/guidelines.xhtml

qualitative results

Thirty participants were interviewed post survey completion (September-November 2020). Just over half were male (n=17), had received a higher education qualification or degree (n=19), lived in Wales (n=25) and were from a White ethnic background (n=23). The average age was 55 years (range 26-76 years). Exemplary quotes are provided in **Table 4**. Definitions of key interview themes relating to symptom experiences, fear of help-seeking and experiences of help-seeking are in **Supplementary file Table S7.**

Symptom experiences

Many participants reported noticing a change to their health or wellbeing during the six months from the start of the first UK lockdown. This was commonly attributed to changes in existing health conditions such as asthma or diabetes or side-effects of medication. This was more notable for non-specific symptoms such as tiredness all the time. As a result, participants delayed their help-seeking, or did not seek help at all, to avoid bothering the doctor when they assumed that they already knew the cause. Even when participants reported red flag symptoms, there was discussion of delaying due to concerns about the NHS being over-stretched. Several participants described accessing other services as a way of easing pressures on their GP practice, for example by phoning 111 or contacting their pharmacist. When making decisions about help-seeking, participants weighed the risks of their clinical need against the risks of catching or exposing others to COVID-19 and burdening the NHS. Some participants conveyed the sentiment that the least they could do to help was to stay away from the NHS.

Fear of help-seeking

All participants expressed fear or nervousness about presenting to primary or secondary care. For some, levels of fear were very high. This was commonly associated with ‘the unknown’ and potentially encountering other members of the public who may not adhere to social distancing guidance. These acted as barriers to timely medical help-seeking. Changes to GP practice procedures invoked worry and hesitancy due to not knowing or understanding the new measures. Examples included the use of new online and telephone triage systems and one-way systems in medical buildings. Participants understood the need for these adaptations, though felt that more support could be provided on how to navigate these changes. Participants expressed particular concern for patients with low digital literacy and those with English as a second language or additional mobility needs.

Fear of attending secondary care was acute for many. Some participants reported being too scared to attend secondary care appointments, treatments or procedures. They made this decision knowing that it could be detrimental to their health and wellbeing. However, those who did attend face-to-face in

For peer review only - http://bmjopen.bmj.com/site/about/guidelines.xhtml

primary and/or secondary care described feeling ‘safe’ and ‘secure’ when attending. Participants expressed surprise that attending was at odds with their expectations of what it was going to be like. Participants described viewing ‘scaremongering’ media reports of hospitals being over-run with coronavirus cases exacerbating their fears. Several participants were saddened that they had been manipulated by the media into feeling scared and avoiding healthcare, with consequences for their health.

Experiences of help-seeking

When participants had contacted their GP, overall they were pleased with the quality of care received and the use of remote consultations. Some were hesitant about disclosing details of their health and medical history before a decision was made about whether they could speak to or see a doctor, feeling that this impacted on their privacy. The use of telephone consultations was praised by most who had received them. Many of these participants reported that it was easier and faster to get a GP appointment than before the pandemic, and that they would like to keep the change to remote consulting on the understanding that face-to-face appointments would be available based on clinical need.

Table 4: Exemplary participant quotes by major theme for symptom experiences, fear of help-seeking and experiences of help-seeking

**Major Theme**	**Exemplary Participant Quotes** (participant ID, gender, age (years), nation of residency) [Quotes provided in intelligent verbatim; P = Participant, I = Interviewer]
**Symptom experiences**	“P: No, apart from the return of the backache … but I think I know why that is, so I haven't done anything about it. Because I know what's going to help it, so as soon as I can go back to the gym, or decide to go back to the gym and start those classes, it will be fine.” *(64021806, Female, 64, Wales)*
	“P: I noticed I was getting increasingly tired… I had a couple of other symptoms as well, which made me think my Levothyroxine dose was now insufficient” *(63984720, Male, 62, Wales)*
	“I: Okay and has the pandemic affected or changed how you think about doctors’ visits and appointments at all?<br>P: I would certainly said I’ve been more reluctant, I would have stayed away and just dealt with it, rather than perhaps going to see a doctor at an early stage.” *(64948240, Female, 46, Wales)*
	“P: ... over the weekend I had a, second time in my life, a bad migraine, and thankfully I'm feeling better but I had thought to myself at what point am I going to go to the GP about not feeling better. And will I ... you know am I less likely to go because they're under strain? And I probably am a bit less likely to go, delay it a little bit longer” *(64078317, Female, 46, England)*

For peer review only - http://bmjopen.bmj.com/site/about/guidelines.xhtml

	"P: … it's certainly changed my mind because like I say I'm of the mindset that says if it's not sort of life threatening critical then, you know, it can wait. So yes, you know I had a certainly different mentality and part of that I think is because of the strain that was put on the health service and all those within it initially that you perhaps didn't want to disturb them" *(65205685, Female, 63, Wales)*
**Fear of help-seeking**	"P: … I haven't been there, the last time I went there, I think it was in the January when I had my annual COPD and CHD review… So, I hadn't been there since, and then I was reading all these horror stories, you know, the stuff we were seeing on the telly. You know the people were going into places, and they didn't even know they had the virus, they wasn't showing symptoms… And passing it on and I was thinking, this could happen to me in the doctor's surgery, but when I actually went to the surgery the whole layout had changed, it had all new furniture put in there, so it could be wiped down." *(65205685, Female, 63, Wales)*
	"P: Well if you're asking about hospital, I was supposed to go to hospital in lockdown see, but the thing is, I was too frightened because of Covid, I thought I'm not going to hospital. And I needed stitches in my knee, because I fell and I landed on both knees in the living room, I fell over the mat. I sliced my knee open, and I needed stitches bad, but I didn't go. My husband used butterfly stitches and done it that way. But I wouldn't go because of Covid see, because I was too frightened, because I didn't want to get Covid." *(64018114, Female, 44, Wales)*
	"P: … I mean my view to hospitals, prior to being in one myself, was that, you know there were people dying all over the place in every ward, every corridor with coronavirus. So yes, I would have been, as I say, certainly very cautious to have, to have wanted to put myself in that situation…. you know I was so impressed with how the hospital were operating when I was in there and, as I say if I'd had vision or understood what it was looking like, how it was working I probably wouldn't have had any concerns at all. I think the hospitals were the safest, safest place to be, is my view after the event, seeing how fantastically well the staff were, you know at following procedure etc… So yes if, you know, if you get that message across that, that a hospital, as I say, is probably the safest place than bloody Tesco's or the local pub or whatever. You know, you're very safe there." *(65205685, Female, 63, Wales)*
**Experiences of help-seeking**	"P: … the surgery did a triage thing, the doctor called me and asked me to go and see them and that worked okay, you know, under the restrictions of the local GP, surgery, you know… They have, they've got, quite stringent processes… Yeah, I was content there, no serious misgivings, you accept their protocols and the new way of doing things and that was fine actually, no problem." *(64026131, Male, 62, Wales)*
	"P: Like I said that assumption a lot of people make as well… They assume that because you're okay, you're seeing them in real life, you're okay talking to them over the video, like I said I, I really don't feel comfortable using those video things. I can't sort of speak normally over them. I feel very disconnected from the person I just, I find it really hard to do." *(64027453, Male, 38, Wales)*
	"P: It has changed the whole system, you can't just make an appointment to go and see somebody, you have to go online, type in briefly what your problem is and then decide whether they call you back or whether they tell you what to do or whether they say I think we should meet face to face. Usually a telephone conversation first and then decide okay perhaps you'd better come down and see me. Which I did once… I think the system works very well actually.<br>I: Do you, so how does it compare then before the pandemic? Could you just make an appointment in those?

For peer review only - http://bmjopen.bmj.com/site/about/guidelines.xhtml

	P: You could but it was always sort of three or four weeks ahead… With the new system, you seem to get some response within the next twenty-four hours which is a big improvement.” *(63986310, Male, 76, Wales)*

DISCUSSION:

We conducted the first population study of cancer symptom experience and help-seeking behaviour during the COVID-19 in the United Kingdom. Among adults surveyed who experienced one or more potential cancer symptom during the first six months of the pandemic, nearly half had not sought help for any symptom from the GP during this time, even for red flag symptoms. Reporting a disability and experiencing more symptoms were associated with higher odds of symptom help-seeking, whereas attributing a symptom(s) to COVID-19 was associated with lower odds. Qualitative data revealed reluctance to contact primary care services due to concerns about catching or transmitting coronavirus and overburdening the NHS. Interviewees described delaying medical help-seeking due to fears that were driven by and exacerbated by media reports of COVID-19 in hospitals.

The prevalence of symptoms experienced over the six month period in the current study was in line with previous studies.[13,29] Symptom help-seeking behaviour during the first six months of the pandemic appeared to be lower than help-seeking reported in the USEFUL study over a 12-month time frame, overall and for individual symptoms such as persistent tiredness and unexplained weight loss, although direct comparison was restricted by methodological differences such as variation in symptom reporting time frames. Similarly to previous research, key help-seeking barriers in the current study included worry about wasting healthcare professionals’ time, over-stretching limited healthcare resources and accessing healthcare services (personal communication).[30,31] International pre-pandemic research on barriers to help-seeking has found that UK adults are more likely to report worry about ‘bothering the doctor’ compared to those in other high-income countries[16]. Participants in our study described putting their health concerns on hold or self-managing conditions and concerns to avoid burdening the NHS, suggesting a compounding of the ‘British stiff upper lip’ phenomenon observed in pre-pandemic research.[16] Novel COVID-specific barriers and attitudes reflecting concerns about COVID-19 infection in healthcare settings and delayed cancer testing were prevalent in both the survey and interviews, but they did not contribute significantly to modelling help-seeking behaviour. Difficulty with remote healthcare consulting was not frequently endorsed; indeed, qualitative findings suggested that when participants had contacted their GP or visited hospital, they reported positive experiences that contrasted with their expectations. Retaining remote consultations alongside face-to-face consultations in future routine healthcare services was favoured.

For peer review only

For peer review only - http://bmjopen.bmj.com/site/about/guidelines.xhtml

The correlates of help-seeking behaviour in this study in part reinforce what has been observed in previous studies. The influence of disability and reporting more symptoms on help-seeking behaviour aligns with previous studies including Hannaford et al. (2020) in which people who were unable to work due to illness or disability were more likely to act on their symptoms. Mechanisms which serve to both increase and decrease timely presentation for symptoms have been previously identified and may vary by nature of comorbidity.[17,32] This relationship was observed in qualitative interviews whereby participants who experienced a new or changing symptom attributed such changes to pre-existing conditions or medications, although it did not deter help-seeking in statistical analyses. In contrast, attributing symptoms to COVID-19 was associated with not contacting the GP and may have been influenced by government messaging to stay at home if experiencing any COVID-like symptoms. The decision not to act on symptoms experienced during the first UK pandemic wave may have been motivated by a desire to protect others in the community from COVID-19 infection, and to prevent healthcare services from being overwhelmed. The finding that current and former smokers were more likely to seek help was similar to findings reported by Hannaford et al. (2020). Although the association did not remain after adjustment in the present study, the consistency of this emerging finding with Hannaford and colleagues warrants investigation in future research. It is possible, for example, that people who currently smoke or have previously smoked perceive an elevated risk status which may prompt presentation. The total number of help-seeking barriers endorsed was not associated with help-seeking behaviour, and more fine-grained analysis of differentiated emotional, practical or service-related barriers is needed.

A key strength of our study was the focus on actual symptoms experienced during the last six months. This reduced the known biases associated with retrospective recall of actual symptoms in patient samples or anticipated responses to hypothetical symptoms in community samples. Pooling data across two surveys provided a large sample that was broadly representative of the British population, noting that being able and willing to complete an online survey was a prerequisite of participation. Despite good representation of ethnic minority groups and people with lower education due to targeted recruitment, we did not observe differences in help-seeking previously identified among these groups.[25,33] This may reflect reduced statistical power to detect such effects because we restricted the analysis to actual symptom-helping among those who had experienced at least one potential cancer symptom. Further research is warranted to examine patterns of help-seeking for individual symptoms or subsets of symptoms (e.g. lung-specific) and the influence of symptom-specific awareness and attributions on help-seeking during the pandemic. We

For peer review only - http://bmjopen.bmj.com/site/about/guidelines.xhtml

acknowledge the restrictions on our ability to compare rates of symptom help-seeking with those reported in the USEFUL study, due to methodological differences including the longer symptom reporting time frame (twelve months) and older age inclusion criteria (>50 years) in the USEFUL study. However, our qualitative findings indicate that people were not coming forward to their GP with symptoms during the first six months of the pandemic. The statistical modelling also showed that attribution of potential cancer symptoms to COVID-19 was associated with lower odds of help-seeking. This pattern may have contributed to the decline in GP referrals for suspected cancer that was observed during 2020.

Evidence from this study highlights the need for continued investment in evidence-led, nationally funded and coordinated cancer awareness campaigns to legitimise seeking help for unusual or persistent symptoms. Clear, consistent information from a trusted source should encourage confidence in contacting the GP promptly, explain the changes to GP practice procedures and what to expect, and alleviate worries about health service capacity and infection control in hospital settings. Credible patient stories with an emphasis on positive outcomes could be important in counteracting possible hyperbolic COVID-19 news reporting and to appropriately recontextualise accounts and support engagement with hospital outpatient appointments, treatments or investigations. Campaigns and other supporting activity could increase uptake and access to remote consulting as it becomes embedded in primary and secondary cancer care.[34] Evaluation of campaign activity and other interventions is essential to ensure that they reach diverse audiences and do not exacerbate inequalities. As the COVID-19 pandemic continues, research must continue to monitor the influences on help-seeking for potential cancer symptoms.

For peer review only

For peer review only - http://bmjopen.bmj.com/site/about/guidelines.xhtml

ACKNOWLEDGEMENTS:

This study was facilitated by HealthWise Wales, the Health and Care Research Wales initiative, which is led by Cardiff University in collaboration with SAIL, Swansea University. HQS is funded by PRIME Centre Wales and GMc by Wales Cancer Research Centre; both Centres are funded by Welsh Government through Health and Care Research Wales. DECIPHer and The Centre for Trials Research receive funding from Health and Care Research Wales and Health and Care Research Wales and Cancer Research UK. JW is funded by a Cancer Research UK Career Development Fellowship (C7492/A17219). We are grateful to Cancer Research UK's Cancer Insights Patient Panel, Clinical Advisory Panel and GP Panel and PRIME Centre Wales SUPER Group for their helpful feedback. Cancer Research UK staff members and other researchers have contributed to the initiation and development of CAM surveys, both historically and for this project. COVID-CAM data was provided by Cancer Research UK who collected the data via Dynata's online survey panels. External Scientific Advisory Group members who advised the statistical analysis plan include: Professor Jamie Brown (University College London), Professor Yoryos Lyratzopoulos (University College London), Dr Katie Robb (University of Glasgow), Dr Christian von Wagner (University College London) and Professor Fiona Walter (University of Cambridge).

COMPETING INTERESTS:

The authors declare no conflict of interest.

FUNDING:

This work was supported by Economic and Social Research Council as part of UK Research and Innovation's Rapid Response to COVID-19 grant number ES/V00591X/1. The funders had no role in the design, conduct or analyses of this study.

AUTHOR CONTRIBUTIONS:

KB (responsible for funding acquisition), RC-J, JT, KLW, JW, HQ-S, KO, GM, MR, JH, VW, AG contributed to the study design, protocol development, study management and planning. MG built the online survey tool and managed the data for the COVID-19 Cancer Help-Seeking and Behaviour Study. VW managed the CRUK COVID-CAM. YM is the CABS study manager. DG and RC-J verified the data and carried out the statistical analysis. YM, HQ-S, JH and GMc collected and analysed the qualitative data. All authors reviewed the data analyses, contributed to data interpretation and writing of the manuscript, and approved the final version of the submitted manuscript.

ETHICAL APPROVAL AND CONSENT TO PARTICIPATE:

For peer review only - http://bmjopen.bmj.com/site/about/guidelines.xhtml

Ethical approval was granted by the School of Medicine Research Ethics Committee, Cardiff University (ref 20.68). Informed consent was provided from all participants at recruitment. This study was conducted in accordance with Good Clinical Practice and the Declaration of Helsinki.

CONSENT FOR PUBLICATION:

Not applicable

DATA SHARING:

De-identified participant data will be made available to the scientific community with as few restrictions as feasible, whilst retaining exclusive use until the publication of major outputs. Data will be available on a public archive.

For peer review only

REFERENCES:

1 Cancer Research UK. Cancer mortality for all cancers combined. CancerStats. 2019.http://www.cancerresearchuk.org/cancer-info/cancerstats/mortality/all-cancers-combined/ (accessed 21 Jan 2021).

2 Ferlay J, Colombet M, Soerjomataram I, *et al.* Estimating the global cancer incidence and mortality in 2018: GLOBOCAN sources and methods. *Int J Cancer* 2019;**144**:1941–53. doi:https://doi.org/10.1002/ijc.31937

3 Swann R, McPhail S, Witt J, *et al.* Diagnosing cancer in primary care: results from the National Cancer Diagnosis Audit. *Br J Gen Pract* 2018;**68**:e63 LP-e72. doi:10.3399/bjgp17X694169

4 Neal RD, Tharmanathan P, France B, *et al.* Is increased time to diagnosis and treatment in symptomatic cancer associated with poorer outcomes? Systematic review. *Br J Cancer* 2015;**112**:S92–107. doi:10.1038/bjc.2015.48

5 Koo MM, Swann R, McPhail S, *et al.* Presenting symptoms of cancer and stage at diagnosis: evidence from a cross-sectional, population-based study. *Lancet Oncol* 2020;**21**:73–9. doi:10.1016/S1470-2045(19)30595-9

6 NHS Cancer waiting times. https://www.england.nhs.uk/statistics/statistical-work-areas/cancer-waiting-times/ (accessed 2 Feb 2021).

7 Monitoring Attendance, INvestigation, Referral and OUTcomes in primary care: impact of and recove1 Monitoring Attendance, INvestigation, Referral and OUTcomes in primary care: impact of and recovery from COVID-19 lockdown (MAINROUTE). https://www.phc.ox.a. https://www.phc.ox.ac.uk/research/cancer-research-group/monitoring-attendance-investigations-referrals-and-outcomes-during-covid-19-mainroute (accessed 30 Apr 2021).

8 Hiom S. How coronavirus is impacting cancer services in the UK. Cancer Res. UK. 2020.https://scienceblog.cancerresearchuk.org/2020/04/21/how-coronavirus-is-impacting-cancer-services-in-the-uk/

9 Jones D, Neal RD, Duffy SRG, *et al.* Impact of the COVID-19 pandemic on the symptomatic diagnosis of cancer: the view from primary care. *Lancet Oncol* 2020;**21**:748–50. doi:10.1016/S1470-2045(20)30242-4

10 Weller D, Vedsted P, Rubin G, *et al.* The Aarhus statement: improving design and reporting of studies on early cancer diagnosis. *Br J Cancer* 2012;**106**:1262–7. doi:10.1038/bjc.2012.68

11 Walter F, Webster A, Scott S, *et al.* The Andersen Model of Total Patient Delay: a systematic review of its application in cancer diagnosis. *J Health Serv Res Policy* 2012;**17**:110–8. doi:10.1258/jhsrp.2011.010113

12 Scott SE, Walter FM, Webster A, *et al.* The Model of Pathways to Treatment:

For peer review only - http://bmjopen.bmj.com/site/about/guidelines.xhtml

Conceptualization and integration with existing theory. *Br J Health Psychol* 2013;**18**:45–65. doi:https://doi.org/10.1111/j.2044-8287.2012.02077.x

13 Hannaford PC, Thornton AJ, Murchie P, Whitaker KL, Adam R, Elliott A. Patterns of symptoms possibly indicative of cancer and associated help-seeking behaviour in a large sample of United Kingdom residents—The USEFUL study. *PLoS One* 2020;**15**.https://doi.org/10.1371/journal.pone.0228033

14 Whitaker KL, Smith CF, Winstanley K, *et al.* What prompts help-seeking for cancer 'alarm' symptoms? A primary care based survey. *Br J Cancer* 2016;**114**:334–9. doi:10.1038/bjc.2015.445

15 Whitaker KL, Scott SE, Winstanley K, Macleod U, Wardle, J. Attributions of Cancer 'Alarm' Symptoms in a Community Sample. *PLoS One* 2014;**9**:1–17.https://doi.org/10.1371/journal.pone.0114028No

16 Forbes LJL, Simon AE, Warburton F, *et al.* Differences in cancer awareness and beliefs between Australia, Canada, Denmark, Norway, Sweden and the UK (the International Cancer Benchmarking Partnership): do they contribute to differences in cancer survival? *Br J Cancer* 2013;**108**:292–300. doi:10.1038/bjc.2012.542

17 Birt L, Hall N, Emery J, *et al.* Responding to symptoms suggestive of lung cancer: a qualitative interview study. *BMJ Open Respir Res* 2014;**1**:e000067. doi:10.1136/bmjresp-2014-000067

18 de Joode K, Dumoulin DW, Engelen V, *et al.* Impact of the coronavirus disease 2019 pandemic on cancer treatment: the patients' perspective. *Eur J Cancer* 2020;**136**:132–9. doi:https://doi.org/10.1016/j.ejca.2020.06.019

19 Helsper CW, Campbell C, Emery J, *et al.* Cancer has not gone away: A primary care perspective to support a balanced approach for timely cancer diagnosis during COVID-19. *Eur J Cancer Care (Engl)* 2020;**29**:e13290–e13290. doi:10.1111/ecc.13290

20 Leventhal H, Brisette I, Leventhal E. *The common sense model of self regulation of health and illness In: CameronL, Leventhal E, eds. The self-regulation of health and illness behaviour.* 2003.

21 CABs protocol. https://osf.io/zxyp3 (accessed 2 Feb 2021).

22 Kelley K, Clark B, Brown V, *et al.* Good practice in the conduct and reporting of survey research. *Int J Qual Heal Care* 2003;**15**:261–6. doi:10.1093/intqhc/mzg031

23 von Elm E, Altman DG, Egger M, *et al.* The Strengthening the Reporting of Observational Studies in Epidemiology (STROBE) Statement: Guidelines for Reporting Observational Studies. *Epidemiology* 2007;**18**.https://journals.lww.com/epidem/Fulltext/2007/11000/The_Strengthening_the_Re

For peer review only - http://bmjopen.bmj.com/site/about/guidelines.xhtml

porting_of_Observational.27.aspx

24 Stubbings S, Robb K, Waller J, *et al.* Development of a measurement tool to assess public awareness of cancer. *Br J Cancer* 2009;**101**:S13–7. doi:10.1038/sj.bjc.6605385

25 CAM 2019. https://osf.io/j67dt/ (accessed 2 Feb 2021).

26 HealthWise Wales. https://www.healthwisewales.gov.wales (accessed 2 Feb 2021).

27 Berinsky AJ, Margolis MF, Sances MW. Separating the shirkers from the workers? Making sure respondents pay attention on self-administered surveys. *Am J Pol Sci* 2014;**58**:739–53. doi:10.1111/ajps.12081

28 Braun V, Clarke V. Using thematic analysis in psychology., 3:2 (2006), 77-101. *Qual Res Psychol* 2006;**3**:77–101. doi:10.1191/1478088706qp063oa

29 Salika T, Lyratzopoulos G, Whitaker KL, *et al.* Do comorbidities influence help-seeking for cancer alarm symptoms? A population-based survey in England. *J Public Health (Bangkok)* 2018;**40**:340–9. doi:10.1093/pubmed/fdx072

30 Connor, K, Hudson, B, Power E. Awareness of the Signs, Symptoms, and Risk Factors of Cancer and the Barriers to Seeking Help in the UK: Comparison of Survey Data Collected Online and Face-to-Face. *JMIR Cancer* 2020;**6**. doi:10.2196/14539

31 Niksic M, Rachet B, Warburton FG, *et al.* Cancer symptom awareness and barriers to symptomatic presentation in England—are we clear on cancer? *Br J Cancer* 2015;**113**:533–42. doi:10.1038/bjc.2015.164

32 Renzi C, Kaushal A, Emery J, *et al.* Comorbid chronic diseases and cancer diagnosis: disease-specific effects and underlying mechanisms. *Nat Rev Clin Oncol* 2019;**16**:746–61. doi:10.1038/s41571-019-0249-6

33 Cancer Research UK - COVID - Survey - Confidence to attend appointments and tests at primary and secondary care settings. https://osf.io/km4ry/ (accessed 2 Feb 2021).

34 Broom A, Kenny K, Page A, *et al.* The Paradoxical Effects of COVID-19 on Cancer Care: Current Context and Potential Lasting Impacts. *Clin Cancer Res* 2020;**26**:5809 LP – 5813. doi:10.1158/1078-0432.CCR-20-2989

For peer review only

For peer review only - http://bmjopen.bmj.com/site/about/guidelines.xhtml

FIGURE LEGEND:

Figure 1: Recruitment flow chart

For peer review only

For peer review only - http://bmjopen.bmj.com/site/about/guidelines.xhtml

Figure 1. Recruitment flow chart

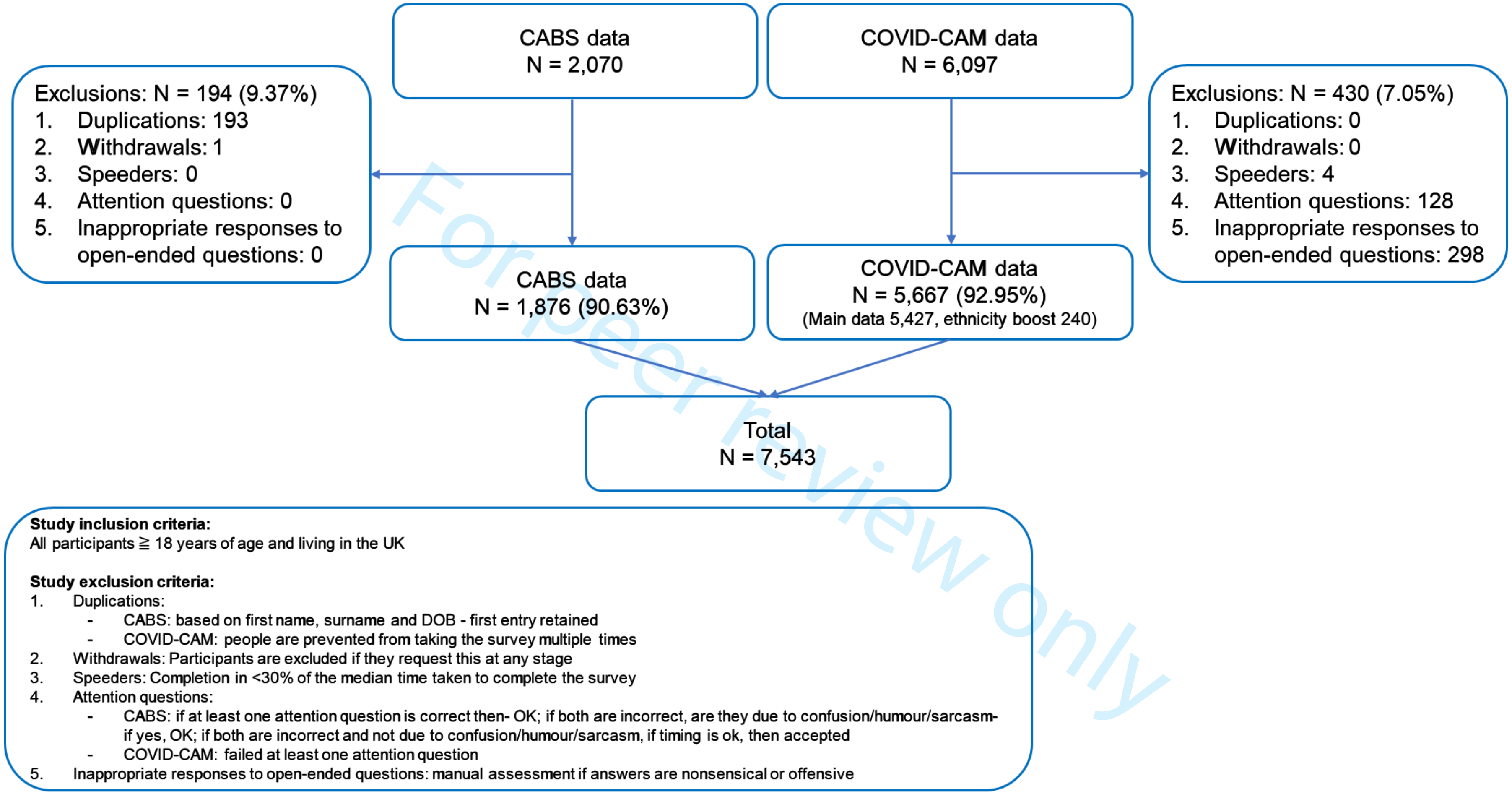

For peer review only

Supplementary File

Table S1: CABS and COVID-CAM survey measures

**Demographic variables** (All participants)	**CABS**	**COVID-CAM**
Age (years)		✓
Date of birth	✓	
UK country (devolved nation), region, ethnicity, gender, marital relationship, educational qualification, disability, experience of cancer, smoking status	✓	✓

**Experience of potential cancer symptoms** Participants were asked if they had experienced any of 15 symptoms over the past six months[a].[1,2]	**Perceived symptom cause** Participants who had experienced any of 8 symptoms were asked what they thought caused the symptom using an open-ended text box from which we coded perceived cancer attribution (cancer/non-cancer) and perceived COVID-19 attribution (COVID-19/non-COVID-19).[3]	**Cancer symptom recognition** Participants were asked "Which of the following if any, do you think could be warning signs or symptoms of cancer?" with response options: Yes, I think this could be a sign of cancer; No, I don't think this could be a sign of cancer; Don't know/unsure.[2] Items were summed to create a total symptom recognition score ranging from 0-15
A persistent change in bowel habits		✓
A persistent change in bladder habits		✓
Tired all the time	✓	✓
Persistent unexplained pain		✓
Unexplained weight loss		✓
A change in the appearance of a mole		✓
An unexplained lump or swelling	✓	✓
Unexplained bleeding	✓	✓
A persistent difficulty swallowing		✓
A sore that does not heal		✓
Coughing up blood	✓	✓
Shortness of breath	✓	✓
Persistent hoarseness	✓	✓
A persistent cough	✓	✓
A change in an existing cough	✓	✓

**Participants who experienced any of the above 15 symptoms were asked:**	**Response options**
**Symptom help-seeking** How long after you first noticed the symptom did you contact the GP about it?[3]	Did not contact the GP/not contacted the GP yet, but plan to /within 1 week of noticing the symptom /within 2 weeks of noticing the symptom /within 1 month of noticing the symptom / within 6 weeks of noticing the symptom /within 3 weeks of noticing the symptom /within 6 months of noticing the symptom/ prefer not to say

For peer review only

For peer review only - http://bmjopen.bmj.com/site/about/guidelines.xhtml

**Barriers to help-seeking – CABS/COVID-CAM**<br>Participants were asked to select as many as applied from a list of barriers experienced the last time they considered seeking medical help[b].[2,4] Items were summed to create a total barriers score ranging from 0-17	
I found it embarrassing talking about my symptoms	
I worried about wasting the healthcare professional's time	
I found it difficult to get an appointment with a particular healthcare professional	
I found it difficult to get an appointment at a convenient time	
I was worried about catching coronavirus[b]	
I was too busy to make time to seek medical attention	
I had too many other things to worry about	
I worried about what they might find wrong with me	
I worried about putting extra strain on the NHS[b]	
I didn't feel confident talking about my symptoms (s)	
I worried they wouldn't take my symptom(s) seriously	
I had symptoms that might have been related to coronavirus[b]	
I didn't want to be seen as someone who makes a fuss	
I didn't want to talk to a receptionist/administrative person about my symptom(s)	
It would have been difficult for me to discuss my health problem remotely (by phone, email or video call)[b]	
I worried about the possibility of having treatment	
I worried about the impact on my employment from taking time off	
Nothing put me off/delayed me in seeking medical attention	
Prefer not to say	
I don't remember	
**Enablers of help-seeking - COVID-CAM only**<br>Participants were asked to select as many as applied from a list of enablers that played a role in their decision to see or speak to a medical professional about their health.[2]	
I had a symptom that I thought might be a sign of cancer	
I had a symptom that was unusual for me	
I had a symptom that was painful	
I knew someone who had a similar symptom, and it turned out to be serious	
I had a symptom that didn't go away	
My friends or family encouraged me to go	
I had a symptom, but I didn't know what was causing it	
I had a symptom that was 'bothersome'	
I had a feeling that something wasn't right	
I had seen information about this symptom in the media (e.g. on tv, radio, posters or magazines)	
I could have a remote consultation (for example, by phone, email or video call)	
Other	
I have never sought medical attention	
I don't remember	
Prefer not to say	
**Attitude towards medical help-seeking during the pandemic**<br>Participants were asked to rate their agreement with items derived from a Cancer Research UK survey[5]	**Response options**
I am confident that I would be safe from coronavirus if I needed to attend an appointment at a hospital	Strongly agree/somewhat agree/somewhat disagree/strongly disagree/I don't know/prefer not to say
I am confident that I would be safe from coronavirus if I needed to attend an appointment at my GP surgery	

For peer review only - http://bmjopen.bmj.com/site/about/guidelines.xhtml

I am worried about delays to cancer tests and investigations caused by coronavirus	

[a] A six-month time frame was selected to include the beginning of the first UK lockdown on 23rd March 2020.[b] Additional COVID-19 specific barriers.

1 Hannaford PC, Thornton AJ, Murchie P, Whitaker KL, Adam R, Elliott A. Patterns of symptoms possibly indicative of cancer and associated help-seeking behaviour in a large sample of United Kingdom residents—The USEFUL study. *PLoS One* 2020; **15**. https://doi.org/10.1371/journal.pone.0228033.

2 CAM 2019. https://osf.io/j67dt/ (accessed Feb 2, 2021).

3 Whitaker KL, Scott SE, Winstanley K, Macleod U, Wardle, J. Attributions of Cancer 'Alarm' Symptoms in a Community Sample. *PLoS One* 2014; **9**: 1–17.

4 Connor, K, Hudson, B, Power E. Awareness of the Signs, Symptoms, and Risk Factors of Cancer and the Barriers to Seeking Help in the UK: Comparison of Survey Data Collected Online and Face-to-Face. *JMIR Cancer* 2020; **6**. DOI:10.2196/14539.

5 Cancer Research UK - COVID - Survey - Confidence to attend appointments and tests at primary and secondary care settings. https://osf.io/km4ry/ (accessed Feb 2, 2021).

For peer review only

For peer review only - http://bmjopen.bmj.com/site/about/guidelines.xhtml

S2 - Interview Topic Guide

Participants will have access to study Information sheets and will have already provided informed consent. Prior to interview commencement the interviewer will re-confirm verbal consent.

The interview will be recorded, anonymised, transcribed confidentially and analysed by members of the research team.

The aim of the interview is to gain further understanding of how participants perceive symptoms, help seeking and behaviour regarding potential cancer symptoms during the COVID-19 lockdown from March 23rd 2020.

It is estimated the interview will be 45 minutes in length. Following the interview participants will be sent a £20 voucher to thank them for their time.

For peer review only

Topic Guide

<u>Symptoms and Help seeking</u>

- Participants' views on symptoms they may have noticed during lockdown and any medical help seeking
(The survey asked about symptom experience with no reference to cancer, to avoid influencing responses – the same applies in the interview)

- For symptoms experienced, participants will be asked for more details, probes regarding timescale of help seeking, perceptions of access to primary care and what influenced their perceptions

- Participants' views about seeking medical help from a health care professional during lockdown, and what this experience was like compared to pre-lockdown consultations including any experience of remote consulting

<u>Screening</u>

- Participants' views on how routine cancer screening programmes were affected during lockdown (breast, bowel, cervical as applicable), their views on screening having been paused, and how has this impacted the relative importance of cancer/cancer screening in the context of pandemic concerns

- What would encourage participants to consider taking part in cancer screening when it resumes, and what may put them off

<u>Health behaviours and Prevention</u>

- Participants' views about any changes to their health-related behaviour (particularly smoking) and what may have influenced any changes (including perceptions of links between coronavirus outcomes and smoking)

- Participants' views on sources of health information and health messaging during lockdown, and their perceived usefulness and credibility

For peer review only - http://bmjopen.bmj.com/site/about/guidelines.xhtml

Table S3: Perceptions of potential cancer symptoms (symptom onset, attribution) by symptom

Data are n (%) and unweighted unless otherwise stated

Potential cancer symptom	Had symptom[1]	Onset of symptom: pre-pandemic[2]	Onset of symptom: during pandemic	Cause of symptom: Cancer	Cause of symptom: COVID-19
	n / 7,543 (%)	n /S (%)	n /S (%)	n /S (%)	n /S (%)
**Non-specific symptom**					
A persistent change in bowel habits[a]	541 (7.2)	186 (34.4)	334 (61.7)	8/405 (2.0)[5]	7/405 (1.7)[5]
A persistent change in bladder habits[a]	450 (6.0)	178 (39.6)	262 (58.2)	<5[5]	<5
Tired all the time[a]	1,603 (21.3)	826 (51.5)	757 (47.2)	16 (1.0)	139 (8.7)
Persistent unexplained pain[a]	662 (8.8)	416 (62.8)	234 (35.3)	<5[5]	6/468 (1.3)[5]
**Non-specific/Red flag symptom**					
Unexplained weight loss[a]	395 (5.2)	76 (19.2)	311 (78.7)	<5[5]	10/335 (3.0)[5]
**Red flag symptom**					
A change in the appearance of a mole[b]	391 (5.2)	88 (22.5)	296 (75.7)	8/294 (2.7)[5]	<5[5]
An unexplained lump or swelling[b]	422 (5·6)	110 (26.1)	301 (71.3)	18 (4.3)	<5
Unexplained bleeding[b]	267 (3.5)	54 (20.2)	195 (73.0)	5 (1.9)	
A persistent difficulty swallowing[b]	237 (3.1)	83 (35.0)	139 (58.6)	<5[5]	<5[5]
A sore that does not heal[b]	291 (3.9)	82 (28.2)	191 (65.6)	<5[5]	<5[5]
**Red flag/Lung-specific symptom**					
Coughing up blood[b]	114 (1.5)	15 (13.2)	78 (68.4)[4]	<5	<5
**Lung-specific symptom**					
Shortness of breath[a]	1,052 (13.9)	510 (48.5)	525 (49.9)	10 (1.0)	89 (8.5)
Persistent hoarseness[a]	200 (2.7)	59 (29.5)	129 (64.5)	<5	8 (4.0)
A persistent cough[a]	444 (5.9)	219 (49.3)	218 (49.1)	<5	43 (9.7)
A change in an existing cough[a]	196 (2.6)	25 (12.8)	159 (81.1)	<5	16 (8.2)

[a] Recommended interval <1 month, [b] Recommended interval <2 weeks based on previous studies of cancer symptom presentation behaviour. [1] Denominator includes those who did not have a symptom and those who preferred not to say (around 1% of the sample). [2] Numbers do not add to 100% - a small proportion (<7%) stated "prefer not to say". For coughing up blood, 18·4% stated prefer not to say. [3] Not at all/A little bit/Moderately concerned; [4] Quite a bit/Extremely concerned. [5] Symptom attribution asked for COVID-CAM sample only.

For peer review only

For peer review only - http://bmjopen.bmj.com/site/about/guidelines.xhtml

Table S4: Barriers to consulting with a medical professional *Data are n (%) and unweighted unless otherwise stated*

**Barriers[1]**	**Pooled sample N=7,543**	**Pooled sample Weighted[2] N=7,543**	**At least one symptom experienced N=3,025**	**No symptoms experienced[3] n=4,428**
I worried about wasting the healthcare professional's time	1,158 (15.4)	930 (12.3)	653 (21.6)	505 (11.4)
I worried about putting extra strain on the NHS	954 (12.6)	790 (10.5)	578 (19.1)	376 (8.5)
I didn't want to be seen as someone who makes a fuss	907 (12.0)	856 (11.3)	540 (17.9)	367 (8.3)
I found it difficult to get an appointment with a particular healthcare professional	774 (10.3)	627 (8.3)	448 (14.8)	326 (7.4)
I worried about catching coronavirus	721 (9.6)	632 (8.4)	415 (13.7)	306 (6.9)
I found it difficult to get an appointment at a convenient time	643 (8.5)	659 (8.7)	321 (10.6)	322 (7.3)
I worried they wouldn't take my symptom(s) seriously	601 (8.0)	574 (7.6)	380 (12.6)	221 (5.0)
I didn't want to talk to a receptionist/administrative person about my symptom(s)	518 (6.9)	458 (6.1)	304 (10.0)	214 (4.8)
I worried about what they might find wrong with me	421 (5.6)	452 (6.0)	231 (7.6)	190 (4.3)
I had too many other things to worry about	401 (5.3)	434 (5.8)	271 (9.0)	130 (2.9)
It would have been difficult for me to discuss my health problem remotely (by phone, email or video call)	361 (4.8)	319 (4.2)	231 (7.6)	130 (2.9)
I found it embarrassing talking about my symptoms	354 (4.7)	384 (5.1)	216 (7.1)	138 (3.1)
I was too busy to make time to seek medical attention	329 (4.4)	354 (4.7)	195 (6.4)	134 (30)
I worried about the possibility of having treatment	304 (4.0)	318 (4.2)	196 (6.5)	108 (2.4)
I didn't feel confident talking about my symptom(s)	272 (3.6)	309 (4.1)	160 (5.3)	112 (2.5)
I worried about the impact on my employment from taking time off	227 (3.0)	252 (3.3)	144 (4.8)	83 (1.9)
I had symptoms that might have been related to coronavirus	143 (1.9)	153 (2.0)	105 (3.5)	38 (0.9)
Nothing put me off/delayed me in seeking medical attention	3,039 (40.3)	2,845 (37.7)	859 (28.4)	2,180 (49.2)
Prefer not to say	114 (1.5)	130 (1.7)	31 (1.0)	83 (1.9)
**Number of barriers to help-seeking reported (0 to 17)**				
Median (25th to 75th centiles); Range	1.0 (1.0 to 2.0); (0 to 14)			

[1] Participants were asked: *"Thinking about the last time you considered seeing or speaking to a medical professional about your health, did any of the following put you off, or make you delay doing so? (This may have been an appointment with a medical professional (e.g. a doctor, nurse or pharmacist) in person, online or over the phone). Please select all that apply".* More than one barrier could be selected. Numbers do not amount to the denominator and percentages do not amount to 100%. [2] All data are weighted to match the adult population in the UK on age, gender, ethnicity, and region. [3] Includes those who preferred not to say.

For peer review only - http://bmjopen.bmj.com/site/about/guidelines.xhtml

Table S5. Help-seeking enablers to consulting with a medical professional *Data are n (%) and unweighted unless otherwise stated*

	COVID-CAM N=5,667	COVID-CAM Weighted[1] N=5,667
I had a symptom that I thought might be a sign of cancer	282 (5.0)	286 (5.0)
I had a symptom that was unusual for me	706 (12.5)	709 (12.5)
I had a symptom that was painful	811 (14.3)	815 (14.4)
I knew someone who had a similar symptom, and it turned out to be serious	122 (2.1)	119 (2.1)
I had a symptom that didn't go away	957 (16.9)	970 (17.1)
My friends or family encouraged me to go	461 (8.1)	454 (8.0)
I had a symptom, but I didn't know what was causing it	683 (12.1)	686 (12.1)
I had a symptom that was 'bothersome'	1,008 (17.8)	1,005 (17.7)
I had a feeling that something wasn't right	721 (12.7)	735 (13.0)
I had seen information about this symptom in the media	137 (2.4)	139 (2.5)
I could have a remote consultation (for example, by phone, email or video call)	448 (7.9)	447 (7.9)
I needed an appointment for a pre-existing problem/condition[2]	687 (12.1)	680 (12.0)
I needed help for a specific symptom or injury[2]	67 (1.2)	70 (1.2)
I needed a women’s health appointment[2]	36 (0.6)	39 (0.7)
I had a symptom that was getting worse[2]	7 (0.1)	8 (0.1)
I needed to have a lab test or get a test result[2]	8 (0.1)	9 (0.2)
Other[3]	144 (2.5)	142 (2.58)
I have never sought medical attention	307 (5.4)	291 (5.1)
I don't remember	798 (14.1)	811 (14.3)
Prefer not to say	150 (2.6)	149 (2.6)

More than one enabler could be selected. Numbers do not amount to the denominator and percentages do not amount to 100%.

[1] All data are weighted to match the adult population in the UK on age, gender, ethnicity, and region. [2] Recoded from the ‘other’ free text option. [3] Reasons that could not be categorised into cohesive themes.

For peer review only - http://bmjopen.bmj.com/site/about/guidelines.xhtml

Table S6: Help-seeking interval by symptom *Data are n (%) and unweighted unless otherwise stated*

Symptom	Had symptom[1]	Contacted the GP	Within 1 week[2]	Within 2 weeks[2]	Within 1 month[2]	Within 6 weeks[2]	Within 3 months[2]	Within 6 months[2]	Contacted within recommended interval[a,b,3]
	n / 7,543 (%)	n / S (%)	n / S (%)	n / S (%)	n / S (%)	n / S (%)	n / S (%)	n / S (%)	n / S (%)
**Non-specific symptom**									
A persistent change in bowel habits[a]	541 (7.2)	254 (47.0)	55 (10.2)	51 (9.4)	42 (7.8)	30 (5.5)	24 (4.4)	52 (9.6)	148 (27.4)
A persistent change in bladder habits[a]	450 (6.0)	227 (50.4)	65 (14.4)	43 (9.6)	32 (7.1)	23 (5.1)	29 (6.4)	35 (7.8)	140 (31.1)
Tired all the time[a]	1,603 (21.3)	540 (33.7)	92 (5.7)	79 (4.9)	95 (5.9)	58 (3.6)	81 (5.1)	135 (8.4)	266 (16.6)
Persistent unexplained pain[a]	662 (8.8)	361 (54.5)	74 (11.2)	68 (10.3)	59 (8.9)	39 (5.9)	52 (7.9)	69 (10.4)	201 (30.4)
**Non-specific/Red flag symptom**									
Unexplained weight loss[a]	395 (5.2)	179 (45.3)	48 (12.2)	45 (11.4)	43 (10.9)	19 (4.8)	10 (2.5)	14 (3.5)	136 (34.4)
**Red flag symptom**									
A change in the appearance of a mole[b]	391 (5.2)	157 (40.2)	37 (9.5)	34 (8.7)	27 (6.9)	18 (4.6)	20 (5.1)	21 (5.4)	71 (18.2)
An unexplained lump or swelling[b]	422 (5.6)	239 (56.6)	81 (19.2)	55 (13.0)	42 (10.0)	11 (2.6)	19 (4.5)	31 (7.3)	136 (32.2)
Unexplained bleeding[b]	267 (3.5)	143 (53.6)	55 (20.6)	22 (8.2)	22 (8.2)	22 (8.2)	8 (3.0)	14 (5.2)	77 (28.8)
A persistent difficulty swallowing[b]	237 (3.1)	128 (54.0)	26 (11.0)	26 (11.0)	31 (13.1)	12 (5.1)	11 (4.6)	22 (9.3)	52 (21.9)
A sore that does not heal[b]	291 (3.9)	128 (44.0)	34 (11.7)	27 (9.3)	22 (7.6)	12 (4.1)	13 (4.5)	20 (6.9)	61 (21.0)
**Red flag/Lung-specific symptom**									
Coughing up blood[b]	114 (1.5)	67 (58.8)	32 (28.1)	11 (9.6)	10 (8.8)	5 (4.4)	5 (4.4)	4 (3.5)	43 (37.7)
**Lung-specific symptom**									
Shortness of breath[a]	1,052 (13.9)	484 (46.0)	123 (11.7)	69 (6.6)	100 (9.5)	50 (4.8)	49 (4.7)	93 (8.8)	292 (27.8)
Persistent hoarseness[a]	200 (2.7)	96 (48.0)	25 (12.5)	18 (9.0)	19 (9.5)	13 (6.5)	8 (4.0)	13 (6.5)	62 (31.0)
A persistent cough[a]	444 (5.9)	230 (51.8)	52 (11.7)	40 (9.0)	46 (10.4)	21 (4.7)	29 (6.5)	42 (9.5)	138 (31.1)
A change in an existing cough[a]	196 (2.6)	100 (51.0)	31 (15.8)	24 (12.2)	19 (9.7)	17 (8.7)	5 (2.6)	4 (2.0)	74 (37.8)

n=number, n/S = number of participants representing each symptom presentation behaviour/number of participants who had this symptom.
[a] Recommended interval <1 month, [b] Recommended interval <2 weeks based on previous studies of cancer symptom presentation behaviour (e.g. Herbert et al., 2018 https://www·thelancet·com/journals/lanchi/article/PIIS2352-4642(18)30004-X/fulltext) and aligned with cancer awareness campaigns (e.g. Be Clear on Cancer https://www·cancerresearchuk·org/health-professional/awareness-and-prevention/be-clear-on-cancer). [1] Denominator includes those who did not have a symptom and those who preferred not to say (around 1% of the sample). [2] Interval contacted GP from noticing the symptom. [3] Subset of participants who 'Contacted the GP in the last 6 months'.

For peer review only - http://bmjopen.bmj.com/site/about/guidelines.xhtml

Table S7: Definitions of themes identified during qualitative interviews with participants relating to symptom experiences, fear of help-seeking and experiences of help-seeking

Theme Identified	Definition
The impact of the pandemic and/or lockdown on general health	Any discussion or reference relating to if and how the pandemic and/or lockdown has impacted the participants general health. Includes changes on beliefs, feelings, views and concerns – physical and mental health
Symptom experience and recognising changes in health or body, deciding to act or not to act on changes in health or body	Any discussion or reference to the participant recognising any changes to their health or body during the pandemic. Why and how the participant decided to act, or not act, of any changes
Actions taken due to changes in health or body	Any discussion or reference made by the participant to actions taken due to changes in their health or body. Includes healthcare and non-healthcare events and information such as who did they see or talk to about their concern
The help-seeking interval	Any discussion or reference on why the participant acted when they did regarding help-seeking or why they waited
Importance of help-seeking	Any discussion or reference made on the relevant importance of help-seeking, especially in reference to the pandemic. Whether the pandemic has affected and/or changed how they think about any aspects of help-seeking. Includes both medical and non-medical help-seeking
Concerns about help-seeking due to the pandemic	Any discussion or reference to the participant having concerns about help-seeking due to the pandemic. This includes healthcare appointments (primary and secondary care) and virtual and remote help-seeking avenues
Experience of face-to-face and virtual/remote consultations in primary and secondary care during the pandemic	Any discussion or reference to participants experience of a face-to-face or virtual/remote healthcare consultation during the pandemic. Includes both primary and secondary healthcare consultations and views on the differences in help/advice/care compared to pre-pandemic
Views and/or ideas that could support/encourage help-seeking and attending healthcare	Any discussion or reference to views/suggestions/ideas provided by participants to support and/or encourage help-seeking and attending healthcare (i.e. possible future facilitators)

For peer review only

For peer review only - http://bmjopen.bmj.com/site/about/guidelines.xhtml

STROBE Statement—Checklist of items that should be included in reports of ***cross-sectional studies***

	Item No	Recommendation	Page No
**Title and abstract**	1	(*a*) Indicate the study's design with a commonly used term in the title or the abstract	1
		(*b*) Provide in the abstract an informative and balanced summary of what was done and what was found	2
**Introduction**			
Background/rationale	2	Explain the scientific background and rationale for the investigation being reported	4-5
Objectives	3	State specific objectives, including any prespecified hypotheses	5
**Methods**			
Study design	4	Present key elements of study design early in the paper	5
Setting	5	Describe the setting, locations, and relevant dates, including periods of recruitment, exposure, follow-up, and data collection	5
Participants	6	(*a*) Give the eligibility criteria, and the sources and methods of selection of participants	5
Variables	7	Clearly define all outcomes, exposures, predictors, potential confounders, and effect modifiers. Give diagnostic criteria, if applicable	6, supp. mat.
Data sources/ measurement	8*	For each variable of interest, give sources of data and details of methods of assessment (measurement). Describe comparability of assessment methods if there is more than one group	6-7, supp. mat.
Bias	9	Describe any efforts to address potential sources of bias	5-7
Study size	10	Explain how the study size was arrived at	6
Quantitative variables	11	Explain how quantitative variables were handled in the analyses. If applicable, describe which groupings were chosen and why	6
Statistical methods	12	(*a*) Describe all statistical methods, including those used to control for confounding	6-7
		(*b*) Describe any methods used to examine subgroups and interactions	n/a
		(*c*) Explain how missing data were addressed	6
		(*d*) If applicable, describe analytical methods taking account of sampling strategy	n/a
		(*e*) Describe any sensitivity analyses	n/a
**Results**			
Participants	13*	(a) Report numbers of individuals at each stage of study—eg numbers potentially eligible, examined for eligibility, confirmed eligible, included in the study, completing follow-up, and analysed	7-17, supp. Mat.
		(b) Give reasons for non-participation at each stage	Supp. Mat.
		(c) Consider use of a flow diagram	Fig 1
Descriptive data	14*	(a) Give characteristics of study participants (eg demographic, clinical, social) and information on exposures and potential confounders	7-9
		(b) Indicate number of participants with missing data for each variable of interest	7-17
Outcome data	15*	Report numbers of outcome events or summary measures	7-17

For peer review only - http://bmjopen.bmj.com/site/about/guidelines.xhtml

Main results	16	(*a*) Give unadjusted estimates and, if applicable, confounder-adjusted estimates and their precision (eg, 95% confidence interval). Make clear which confounders were adjusted for and why they were included	13-17, Table 3
		(*b*) Report category boundaries when continuous variables were categorized	Table 3
		(*c*) If relevant, consider translating estimates of relative risk into absolute risk for a meaningful time period	n/a
Other analyses	17	Report other analyses done—eg analyses of subgroups and interactions, and sensitivity analyses	n/a
**Discussion**			
Key results	18	Summarise key results with reference to study objectives	21
Limitations	19	Discuss limitations of the study, taking into account sources of potential bias or imprecision. Discuss both direction and magnitude of any potential bias	22-23
Interpretation	20	Give a cautious overall interpretation of results considering objectives, limitations, multiplicity of analyses, results from similar studies, and other relevant evidence	21-23
Generalisability	21	Discuss the generalisability (external validity) of the study results	22-23
**Other information**			
Funding	22	Give the source of funding and the role of the funders for the present study and, if applicable, for the original study on which the present article is based	24

*Give information separately for exposed and unexposed groups.

Note: An Explanation and Elaboration article discusses each checklist item and gives methodological background and published examples of transparent reporting. The STROBE checklist is best used in conjunction with this article (freely available on the Web sites of PLoS Medicine at http://www.plosmedicine.org/, Annals of Internal Medicine at http://www.annals.org/, and Epidemiology at http://www.epidem.com/). Information on the STROBE Initiative is available at www.strobe-statement.org.

For peer review only

BMJ Open

Cancer symptom experience and help-seeking behaviour during the COVID-19 pandemic in the United Kingdom: a cross-sectional population survey

Journal:	*BMJ Open*
Manuscript ID	bmjopen-2021-053095.R1
Article Type:	Original research
Date Submitted by the Author:	14-Jul-2021
Complete List of Authors:	Quinn-Scoggins, H; Cardiff University, PRIME Centre Wales, Division of Population Medicine, School of Medicine<br>Cannings-John, Rebecca; Cardiff University Centre for Trials Research<br>Moriarty, Yvonne; Cardiff University, Centre for Trials Research<br>Whitelock, Victoria; Cancer Research UK, Cancer Intelligence<br>Whitaker, Katriina; University of Surrey, School of Health Sciences<br>Grozeva, Detelina; Cardiff University, Centre for Trials Research<br>Hughes, Jacqueline; Cardiff University, Centre for Trials Research<br>Townson, Julia; Cardiff University, Centre for Trials Research<br>Osborne, Kirstie; Cancer Research UK, Cancer Intelligence<br>Goddard, Mark; Cardiff University, Centre for Trials Research<br>McCutchan, GM; Cardiff University, Division of Population Medicine, School of Medicine<br>Waller, Jo; King's College London, School of Cancer and Pharmaceutical Sciences<br>Robling, Michael; Cardiff University, Centre for Trials Research; Cardiff University, DECIPHer (Centre for Development, Evaluation, Complexity and Implementation in Public Health Improvement), School of Social Sciences<br>Hepburn, Julie; Health and Care Research Wales Support Centre, Public Involvement Community<br>Moore, Graham; Cardiff University, DECIPHer (Centre for Development, Evaluation, Complexity and Implementation in Public Health Improvement), School of Social Sciences<br>Gjini, Ardiana; Public Health Wales; Cardiff University<br>Brain, Kate; Cardiff University, PRIME Centre Wales, Division of Population Medicine, School of Medicine
**Primary Subject Heading**:	Oncology
Secondary Subject Heading:	Public health
Keywords:	PREVENTIVE MEDICINE, PUBLIC HEALTH, STATISTICS & RESEARCH METHODS, ONCOLOGY, PRIMARY CARE, COVID-19

SCHOLARONE™
Manuscripts

I, the Submitting Author has the right to grant and does grant on behalf of all authors of the Work (as defined in the below author licence), an exclusive licence and/or a non-exclusive licence for contributions from authors who are: i) UK Crown employees; ii) where BMJ has agreed a CC-BY licence shall apply, and/or iii) in accordance with the terms applicable for US Federal Government officers or employees acting as part of their official duties; on a worldwide, perpetual, irrevocable, royalty-free basis to BMJ Publishing Group Ltd ("BMJ") its licensees and where the relevant Journal is co-owned by BMJ to the co-owners of the Journal, to publish the Work in this journal and any other BMJ products and to exploit all rights, as set out in our licence.

The Submitting Author accepts and understands that any supply made under these terms is made by BMJ to the Submitting Author unless you are acting as an employee on behalf of your employer or a postgraduate student of an affiliated institution which is paying any applicable article publishing charge ("APC") for Open Access articles. Where the Submitting Author wishes to make the Work available on an Open Access basis (and intends to pay the relevant APC), the terms of reuse of such Open Access shall be governed by a Creative Commons licence – details of these licences and which Creative Commons licence will apply to this Work are set out in our licence referred to above.

Other than as permitted in any relevant BMJ Author's Self Archiving Policies, I confirm this Work has not been accepted for publication elsewhere, is not being considered for publication elsewhere and does not duplicate material already published. I confirm all authors consent to publication of this Work and authorise the granting of this licence.

For peer review only

Cancer symptom experience and help-seeking behaviour during the COVID-19 pandemic in the United Kingdom: a cross-sectional population survey

Harriet Quinn-Scoggins*[1], Rebecca Cannings-John[*2], Yvonne Moriarty[2], Victoria Whitelock[3], Katriina L Whitaker[4], Detelina Grozeva[2], Jacqueline Hughes[2], Julia Townson[2], Kirstie Osborne[3], Mark Goddard[2], Grace McCutchan[5], Jo Waller[6], Michael Robling[2,7], Julie Hepburn[8], Graham Moore[7], Ardiana Gjini[9,10], Kate Brain[1]

*Dr Harriet Quinn-Scoggins and Dr Rebecca Cannings-John share equally first authorship of this manuscript

Corresponding author: Professor Kate Brain; Neuadd Meirionnydd, Heath Park, Cardiff, CF14 4YS, UK; BrainKE@Cardiff.ac.uk; ORCiD ID: 0000-0001-9296-9748

Author affiliations:

[1] PRIME Centre Wales, Division of Population Medicine, School of Medicine, Cardiff University, Neuadd Meirionnydd, Heath Park, Cardiff CF14 4YS, UK

[2] Centre for Trials Research, Cardiff University, Neuadd Meirionnydd, Heath Park, Cardiff CF14 4YS, UK

[3] Cancer Intelligence, Cancer Research UK, 2 Redman Place, London, E20 1JQ, UK

[4] School of Health Sciences, University of Surrey, Guildford, UK

[5] Wales Cancer Research Centre, Division of Population Medicine, School of Medicine, Cardiff University, Neuadd Meirionnydd, Heath Park, Cardiff CF14 4YS, UK

[6] Cancer Prevention Group, School of Cancer and Pharmaceutical Sciences, King's College London, Great Maze Pond, London SE1 9RT, UK

[7] DECIPHer (Centre for Development, Evaluation, Complexity and Implementation in Public Health Improvement), School of Social Sciences, Cardiff University, 1-3 Museum Place, Cardiff UK

[8] Public Involvement Community, Health and Care Research Wales Support Centre, Castlebridge 4, 15-19, Cowbridge Road East, Cardiff CF11 9AB, UK

[9] Public Health Wales, 2 Capital Quarter, Tyndall Street, Cardiff CF10 4BZ, UK

[10] Cardiff University, Neuadd Meirionnydd, Heath Park, Cardiff CF14 4YS, UK

Key words: COVID-19; Preventive medicine; Public Health; Statistics & Research Methods; Oncology; Primary Care

Word count: 5139/4000 words

For peer review only

For peer review only - http://bmjopen.bmj.com/site/about/guidelines.xhtml

ABSTRACT (275/300)

Objectives: To understand self-reported potential cancer symptom help-seeking behaviours and attitudes during the first 6 months (March – August 2020) of the UK COVID-19 pandemic.

Design: UK population-based survey conducted during August and September 2020. Correlates of help-seeking behaviour were modelled using logistic regression in participants reporting potential cancer symptoms during the previous six months. Qualitative telephone interviews with a purposeful subsample of participants, analysed thematically.

Setting: Online UK wide survey.

Participants: 7,543 adults recruited via Cancer Research UK online panel provider (Dynata) and HealthWise Wales (a national register of 'research ready' participants) supplemented with social media (Facebook and Twitter) recruitment. 30 participants were also interviewed.

Main outcome measures: Survey measures included experiences of 15 potential cancer symptoms, help-seeking behaviour, barriers and prompts to help-seeking.

Results: Of 3,025 (40.1%) participants who experienced a potential cancer symptom, 44.8% (1,355/3,025) had not contacted their GP. Odds of help-seeking were higher among participants with disability (adjusted odds ratio (aOR)=1.38, 95% CI 1.11-1.71) and who experienced more symptoms (aOR=1.68, 95% CI 1.56-1.82), and lower among those who perceived COVID-19 as the cause of symptom(s) (aOR=0.36, 95% CI 0.25-0.52). Barriers included worries about wasting the doctor's time (1,158/7,543, 15.4%), putting strain on healthcare services (945, 12.6%) and not wanting to make a fuss (907, 12.0%). Interviewees reported reluctance to contact the GP due to concerns about COVID-19 and fear of attending hospitals, and described putting their health concerns on hold.

Conclusions: Many people avoided healthcare services despite experiencing potential cancer symptoms during the COVID-19 pandemic. Alongside current help-seeking campaigns, well-timed and appropriate nationally co-ordinated campaigns should signal that services are open safely for those with unusual or persistent symptoms.

Registration: ISRCTN17782018

For peer review only - http://bmjopen.bmj.com/site/about/guidelines.xhtml

ARTICLE SUMMARY:

Strengths and Limitations of this study:

- To our knowledge this is the first UK population survey of the impact of COVID-19 on help-seeking for potential cancer symptoms.
- A large sample was recruited across two online surveys and data pooled where applicable, providing a larger dataset for analysis which was broadly representative of the UK population.
- Data collection occurred between August and September 2020 and thus on the first lockdown period in the UK.
- We assessed self-report of actual symptoms experienced during the first 6 months of the pandemic, reducing the known biases associated with retrospective recall of symptoms in patient samples or anticipated responses to hypothetical symptoms in community samples.
- Survey data were supplemented with in-depth qualitative interviews, providing rich insight and context regarding symptom help-seeking behaviour during the pandemic.

For peer review only

BACKGROUND:

Cancer is the leading cause of mortality in the United Kingdom (UK)[1] and globally.[2] In countries with a ‘gatekeeper’ healthcare system such as the UK, most cancers are diagnosed symptomatically through primary care.[3] Diagnosing symptomatic cancer earlier can enable more timely treatment with better clinical outcomes across a range of cancers.[4,5] However, this route to cancer early diagnosis has been severely disrupted during the COVID-19 pandemic. Large reductions in demand for primary care services were noted[6] and estimates suggest that there were more than 380,000 fewer urgent suspected cancer referrals in the UK between March 2020 and March 2021, a reduction of approximately 13% compared with pre-pandemic levels [CRUK Cancer Intelligence Team, Evidence of the impact of COVIS-19 across the cancer pathway: Key stats, 2021]. Modelling of cancer diagnostic delays in England estimate a substantial increase in the number of avoidable cancer deaths over the next five years due to the COVID-19 pandemic.[7] This has led to concerns that members of the public may not be coming forward to their General Practitioner (GP) with potential cancer symptoms due to factors including fear of coronavirus infection and concerns about placing additional burden on the National Health Service.[8]

During the first UK lockdown from March 2020, the UK government message to *“stay home, protect the NHS, save lives”* was intended to control the spread of COVID-19, but potentially sent a strong signal to the public that cancer can wait.[9] Consequently, the pandemic is likely to have affected key stages across the cancer diagnostic pathway[10] including the patient interval.[11] As set out in the Model of Pathways to Treatment,[12] the patient interval combines the time between a person noticing a bodily change or symptom to perceiving a reason to seek medical help (the appraisal interval), and the time between perceiving a reason to seek medical help to first contact with a medical professional (the help-seeking interval). In UK studies conducted before the COVID-19 pandemic, rates of self-reported symptom help-seeking in adults aged over 50 years ranged from 26.5% seeking help from their GP for at least one potential cancer symptom over a one month period,[13] to 60% over twelve months[13] and 67% over three months.[14] Adverse impact of the pandemic on people’s willingness to seek help for potential cancer symptoms seems likely, especially for non-specific or respiratory symptoms that are similar to COVID-19 symptoms such as a persistent or changing cough, fatigue and breathlessness. Evidence from pre-COVID studies suggests that non-specific symptoms such as those previously mentioned may be overlooked or dismissed,[15] in part due to worry about wasting the doctor’s time.[16] In adults with existing respiratory and cardiac comorbid conditions, potential cancer symptoms may be misattributed and not acted on.[17] Fear of COVID-19 infection may also deter attendance in healthcare settings, especially among high risk and shielding groups.[18] Changes to healthcare service

For peer review only - http://bmjopen.bmj.com/site/about/guidelines.xhtml

delivery during the pandemic, including remote GP consultations, may create additional barriers to accessing services.[19] We therefore anticipated that the UK adult population would be more reluctant to seek help for potential cancer symptoms than before the pandemic.

Evidence is needed regarding public perceptions of potential cancer symptoms and symptom help-seeking behaviour, and potential inequalities in help-seeking, to understand the factors driving reduced primary care service use in the UK during COVID-19. We conducted a large-scale population survey to examine self-reported symptom help-seeking attitudes and behaviour in a UK adult cohort during the pandemic. Selection of survey measures and framing of qualitative interview topics were guided by relevant health psychology theories including the Model to Pathways to Treatment[11] and Common Sense Model of Self-Regulation.[20] In addition, we compared the overall proportion of participants seeking help during the first pandemic wave with UK pre-pandemic data reported in the USEFUL study.[13]

METHODS:

study design

A prospective, mixed-methods observational cohort study in the UK population during the COVID-19 pandemic. The study protocol and analysis plans were pre-registered on Open Science Framework.[21] Findings are reported in accordance with the STROBE guidelines for surveys and observational studies.[22,23]

survey participants and procedures

Two cross-sectional online surveys were conducted in parallel, the COVID-19 Health and Help-Seeking Behaviour Study (CABS) and the Cancer Research UK (CRUK) COVID-19 Cancer Awareness Measure (COVID-CAM). COVID-CAM was based on CRUK's Cancer Awareness Measure 2019.[24,25] Key measures were aligned where possible across the two surveys, and data pooled where appropriate. Eligible participants were aged 18 years or over (due to collecting additional survey data on attitudes and behaviours relating to cancer prevention and cervical screening), resident in the UK and able to speak English. Data were collected between 6th August and 18th September 2020, after the first UK lockdown which started on 23rd March 2020. Study information was available online prior to participants providing electronic informed consent online.

Participants were recruited to the CABS survey via HealthWise Wales (HWW, a national register of 'research ready' participants)[26] and social media (Facebook and Twitter). Potentially under-

For peer review only - http://bmjopen.bmj.com/site/about/guidelines.xhtml

For peer review only

represented groups including men, smokers, black, Asian and minority ethnic groups, and people living in socioeconomically deprived areas were targeted by HWW using personalised emails and Facebook-targeted advertising. Participants were recruited to the COVID-CAM survey via Dynata, an online panel provider (www.dynata.com). Quotas were placed on age, gender, social grade and UK region to recruit a nationally representative sample and sample size for ethnic minority groups was increased (relative to UK population statistics) to increase representation.

patient and public involvement

Patient and Public Involvement (PPI) was included at all stages from conceptualisation through to data interpretation. Working alongside CRUK's Cancer Insights Panel, the Wales Centre for Primary and Emergency Care Research PPI Group (Service Users for Primary and Emergency Care Research Group) and our study PPI co-applicant (JHep), all public-facing materials including study information, consent procedures, survey and interview topic guides were reviewed and amended as appropriate. Our PPI co-applicant were also involved in results interpretation and how best to disseminate these to the wider population (video animation and infographic planned).

survey measures

Selection of measures was guided by clinical and academic expertise from the study management group, including our PPI groups and PPI co-applicant. New COVID-19-specific survey items were tested with PPI group members for acceptability prior to inclusion in the survey.

Measures were obtained from all participants across both CABS and COVID-CAM surveys unless otherwise stated. Data only collected in CABS are denoted by ~ and in COVID-CAM by *. Where relevant, a six-month time frame was selected to include the beginning of the first UK lockdown on 23rd March 2020. Two attention check questions were included in both surveys.[27]

Demographic and health-related factors. Participants were asked in which region of the UK they lived, their date of birth~/age*, gender, ethnicity, marital relationship, highest educational qualification, and whether they considered themselves to have a disability. Experience of cancer was recorded by asking participants if they, anyone in their family or any of their friends had cancer. Smoking status was captured as never, former or current smoker.

Symptom experience. Participants were asked if they had experienced any of the following 15 symptoms over the past six months: a persistent change in bowel habits, a persistent change in bladder habits, tiredness all the time, persistent unexplained pain, unexplained weight loss, a change in the appearance

For peer review only - http://bmjopen.bmj.com/site/about/guidelines.xhtml

of a mole, an unexplained lump or swelling, unexplained bleeding, a persistent difficulty swallowing, a sore that does not heal, coughing up blood, shortness of breath, persistent hoarseness, a persistent cough, a change in an existing cough. The symptoms included were based on those in Connor et al. [28] and included a range of non-specific, red flag and lung-specific symptoms. Response options were Yes, No or Prefer not to say.[15]

Symptom help-seeking. For each symptom experienced, participants were asked '*How long after you first noticed the symptom did you contact the GP about it?*' Response options included: Did not contact the GP; Not contacted the GP yet but plan to; Within 1 week of noticing the symptom; Within 2 weeks of noticing the symptom; Within 1 month of noticing the symptom; Within 6 weeks of noticing the symptom; Within 3 months of noticing the symptom; Within 6 months of noticing the symptom; Prefer not to say.[15] The method of categorising symptom help-seeking was based on the USEFUL study.[13] Outcomes were dichotomised as 'contacted GP in the last six months' versus 'no contact' for individual symptoms. For the composite outcome of GP contact across all symptoms, the outcome was 'contacted GP in the last six months for at least one symptom' versus 'no contact for any symptoms'.

Perceived symptom cause. Participants who had experienced any of the eight following symptoms were asked what they thought caused the symptom using free text[15]: tired all the time, an unexplained lump or swelling, unexplained bleeding, coughing up blood, shortness of breath, persistent hoarseness, a persistent cough, a change in an existing cough. Free text responses were independently coded by HQS, GMc and YM into attribution categories[15]: cancer suspicion, COVID-19 (physical), COVID-19 (psychological), physical (non-cancer), psychological, external/normalising, don't know, exclude. Following independent coding of the first 20% of the data, Cohen's Kappa was used to assess the degree of inter-rater reliability per symptom. Inter-rater reliability was high for all symptoms (>0.80)[29] so no adjustments were made. For the purposes of the current study, symptom attribution categories were merged to create two variables: perceived cancer attribution (cancer/not cancer) and perceived COVID-19 attribution (COVID-19/not COVID-19).[15]

Symptom recognition. For all 15 potential cancer symptoms, participants were asked '*Which of the following, if any, do you think could be warning signs or symptoms of cancer?'* Response options were: Yes, I think this could be a sign of cancer; No, I don't think this could be a sign of cancer; Don't know; Unsure.[25] Items were summed to create a total symptom recognition score ranging from 0-15.

For peer review only - http://bmjopen.bmj.com/site/about/guidelines.xhtml

Barriers and prompts to help-seeking. Participants were asked to select as many as applied from a list of 19 barriers experienced the last time they considered seeking medical help (Supplementary material Table S1).[25,28] Examples of barriers include 'I found it embarrassing talking about my symptoms', 'I worried about wasting the health professional's time' and 'I had symptoms that might have been related to coronavirus'. Response options included 'Nothing put me off/delayed me in seeking medical attention' and 'Prefer not to say'. Participants were asked to select as many as applied from a list of 20 prompts* that played a role in their decision to see or speak to a medical professional about their health (Supplementary material Table S2).[25] Examples of prompts include 'I had a symptom that I thought might be a sign of cancer', 'I had a symptom that was unusual for me' and 'I could have a remote consultation (for example, by phone, email or video call)'. Response options included 'Other', 'I have never sought medical attention', 'I don't remember' and 'Prefer not to say'.

Attitudes towards medical help-seeking during the pandemic. Participants were asked to rate their agreement with three items derived from a Cancer Research UK survey[30]: 'I am confident that I would be safe from coronavirus if I needed to attend an appointment at a hospital'; 'I am confident that I would be safe from a coronavirus if I needed to attend an appointment at my GP surgery'; 'I am worried about delays to cancer tests and investigations caused by coronavirus'. A 4-point Likert scale (where 1=strongly agree and 4=strongly disagree) was used to assess agreement with each statement, with additional options of Don't know and Prefer not to say.

qualitative interviews

Survey participants who consented to interview were purposively sampled from the CABS study cohort according to age, gender and symptom experience. Consent for interview and audio-recording was reconfirmed verbally. A semi-structured topic guide (Supplementary material S3) was used to explore participants' views on attending primary and secondary healthcare in light of the COVID-19 pandemic, contextual influences on help-seeking and strategies to encourage future help-seeking. We aimed to recruit thirty participants in order to gain an in-depth understanding of views, whilst considering purposeful sampling to provide a range of participant demographic characteristics and symptom experiences. Interview participants were reimbursed with a £20 voucher. Transcribed anonymised data were thematically analysed[31] Inductive data-driven codes and *a priori* deductive theory-driven codes were used to extrapolate themes. NVivo12 (QSR international) was used as an aide to data organisation. Data were coded by HQS, JHug and YM with 20% independently dual coded.

sample size

For peer review only - http://bmjopen.bmj.com/site/about/guidelines.xhtml

The study was powered to examine the correlates symptom help-seeking in those who experienced one or more potential cancer symptoms using a multivariable logistic regression model containing 15 candidate predictors.[32] For an outcome proportion of 0.20, the max(R^2_{cs}) value is 0.63. If we assume, conservatively, that the model will explain 15% of the variability, the anticipated R^2_{cs} value is 0.15×0.63=0.095. This indicated that at least 1,345 responders were required, corresponding to 269 events and an event per predictor parameter (EPP) of 17.93. Inflating the sample size based on an estimated 20% symptom prevalence within a three-month period,[33] the final sample size required for the primary survey analysis was 6,725.

statistical analysis

Analyses were conducted using SPSS 25.0 and Stata 16.0. Data were weighted to match the UK population profile on age, gender, ethnicity and country (i.e. devolved nation) using English 2011 Census and Office for National Statistics mid-year estimates. Cases with missing data were excluded on a per-analysis basis. Descriptive analyses were used to identify sample characteristics, prevalence of potential cancer symptoms, help-seeking prompts and barriers (including a total barriers score ranging from 0-17) and, among those who had experienced potential cancer symptoms, symptom perceptions and help-seeking behaviour. Sample characteristics and symptom prevalence are presented unweighted and weighted. Due to similar estimates, subsequent analyses are presented as unweighted.

correlates of symptom help-seeking behaviour

Descriptive summary statistics and logistic regression models were used to estimate the prevalence and odds respectively of GP help-seeking in those who had experienced at least one symptom (compared to not seeking help for any of their symptoms). The following key factors were examined: age group, gender, ethnicity, country, region, education, smoking status, marital relationship, disability, cancer status (self, family and friends), perceived symptom causes (cancer or COVID-19), barriers towards medical help-seeking, confidence in attending hospital and GP, delays in test results, and cancer symptom recognition. We additionally fitted multivariable regression models to explore the independent contribution of potential factors by including all factors as independent variables to account for potential confounding of crude associations by other variables. The study was designed to fit descriptive models, capturing the association between dependent and independent variables, rather than for prediction or causality. Multi-collinearity between factors was assessed using the Variance Inflation Factor (VIF) (VIF >4 warrants further investigation). Data are reported as crude and adjusted odds ratios (ORs) with 95% confidence intervals (CIs).

For peer review only - http://bmjopen.bmj.com/site/about/guidelines.xhtml

RESULTS:

characteristics of participants

A total of 8,167 participants responded to the survey in August and September, of whom 7,543 (92.4%) were included **(Figure 1)**. Demographic characteristics of the pooled sample (n=7,543) and by recruitment route are shown in **Table 1**. Almost half the unweighted pooled sample was age 55 years and over (n=3,574, 47.4%) and female (3,709, 49.2%). Most were of White ethnic background (6,685, 88.6%) and living in England (4,904, 65.0%). Over one third had university level education or higher (2,892, 38.3%) and around two thirds were married or cohabiting (4,864, 64.5%). Current smokers and former smokers comprised 18.8% (1,417) and 32.3% (2,435) of the sample, respectively. Under a fifth (1,284, 17.4%) reported having a disability and 8.7% (657) had experienced cancer themselves.

Table 1: Sample characteristics

Data are n (%) and unweighted unless otherwise stated

	Pooled sample N=7,543	Pooled sample weighted[1] N=7,543	CABS N=1,876	COVID-CAM N=5,667
**Age (years)**				
18-24	543 (7.2)	665 (8.8)	12 (0.6)	531 (9.4)
25-34	945 (12.5)	1,345 (17.8)	53 (2.8)	892 (15.7)
35-44	1,149 (15.2)	1,420 (18.8)	132 (7.0)	1,017 (17.9)
45-54	1,221 (16.2)	1,420 (18.8)	202 (10.8)	1,019 (18.0)
55-64	1,282 (17.0)	1,194 (15.8)	417 (22.2)	865 (15.3)
65-74	1,795 (23.8)	816 (10.8)	738 (39.3)	1,057 (18.7)
75+	497 (6.6)	590 (7.8)	271 (14.4)	226 (4.0)
Missing/other/prefer not to say	111 (1.5)	93 (1.2)	51 (2.7)	60 (1.1)
**Gender**				
Male	3,807 (50.5)	3,681 (48.8)	1,044 (55.7)	2,763 (48.8)
Female	3,709 (49.2)	3,832 (50.8)	827 (44.1)	2,882 (50.9)
Non-binary, transgender female or other	27 (0.4)	29 (0.4)	5 (0.3)	22 (0.4)
**Ethnicity**				
White	6,685 (88.6)	6,948 (92.1)	1,821 (97.1)	4,864 (85.8)
Mixed/Multiple ethnic groups	143 (1.9)	153 (2.0)	19 (1.0)	124 (2.2)
Asian/Asian British	458 (6.1)	274 (3.6)	15 (0.8)	443 (7.8)
Black/African/Caribbean/Black British	154 (2.0)	135 (1.8)	14 (0.7)	150 (2.6)
Other ethnic group	96 (1.3)	26 (0.3)		86 (1.5)
Prefer not to say	7 (0.1)	8 (0.1)	7 (0.4)	NA
**Country/Region**				
England	4,904 (65.0)	6,311 (83.7)	76 (4.1)	4,828 (85.2)
Wales	2,045 (27.1)	376 (5.0)	1,797 (95.8)	248 (4.4)
Scotland	456 (6.0)	601 (8.0)		453 (8.0)

For peer review only - http://bmjopen.bmj.com/site/about/guidelines.xhtml

Northern Ireland	105 (1.4)	225 (3.0)		105 (1.9)
England:				
North East England	265 (3.5)	376 (5.0)		265 (4.7)
North West England	621 (8.2)	826 (11.0)	19 (1.0)	618 (10.9)
Yorkshire and Humberside	479 (6.4)	526 (7.0)		476 (8.4)
East Midlands	417 (5.5)	601 (8.0)		415 (7.3)
East Anglia	503 (6.7)	676 (9.0)		500 (8.8)
West Midlands	513 (6.8)	676 (9.0)		508 (9.0)
South East England	830 (11.0)	1,052 (13.9)	24 (1.3)	806 (14.2)
South West England	473 (6.3)	601 (8.0)	9 (0.5)	464 (8.2)
London	803 (10.6)	977 (12.9)	27 (1.4)	776 (13.7)
Prefer not to say	33 (0.4)	30 (0.4)	0 (0.0)	33 (0.6)
**Highest level of education**				
Degree or higher degree	2,892 (38.3)	2,713 (36.0)	897 (47.8)	1,995 (35.2)
A levels or further education	2,447 (32.4)	2,537 (33.7)	542 (28.9)	1,905 (33.6)
O levels/GCSEs	1,565 (20.7)	1,694 (22.5)	268 (14.3)	1,297 (22.9)
No formal qualifications	412 (5.5)	390 (5.2)	105 (9.6)	307 (5.4)
Still studying	81 (1.1)	87 (1.2)	9 (0.5)	72 (1.3)
Prefer not to say	74 (1.0)	65 (0.9)	26 (1.4)	48 (0.8)
Other	72 (1.0)	55 (0.7)	29 (1.6)	43 (0.8)
**Smoking status**				
Never smoked	3,586 (47.5)	3,601 (47.7)	842 (45.9)	2,744 (48.4)
Former smoker	2,435 (32.3)	2,157 (28.6)	839 (44.7)	1,596 (28.2)
Current smoker	1,417 (18.8)	1,706 (22.6)	150 (8.0)	1,267 (22.4)
Other/prefer not to say	105 (1.4)	79 (1.0)	45 (2.3)	60 (1.1)
**Marital relationship**				
Not married or cohabiting	2,632 (34.9)	2,750 (36.5)	561 (29.9)	1,978 (36.4)
Married or cohabiting	4,864 (64.5)	4,760 (63.1)	1302 (69.4)	3,417 (63.0)
Prefer not to say	47 (0.6)	33 (0.4)	13 (0.7)	32 (0.6)
**Disability**				
No	6,079 (82.6)	6,136 (83.4)	1,445 (78·7)	4,634 (83.8)
Yes	1,284 (17.4)	1,223 (16.6)	390 (21·3)	894 (16.2)
**Experience of cancer**				
No	1,745 (23.1)	2,000 (26.5)	157 (8.3)	1,558 (28.0)
Yes, other (family and friends)[2]	5,141 (68.2)	5029 (66.7)	1,460 (77.8)	3,681 (65.0)
Yes, self	657 (8.7)	512 (6.8)	259 (14.9)	398 (7.0)

Abbrev: CABS = COVID-19 Health and Help-Seeking Behaviour Study cohort recruited via HealthWise Wales and social media; COVID-CAM = Cancer Research UK's COVID-19 Cancer Awareness Measure sample recruited via Dynata, an online panel provider; NA = Not available as an option.

[1] All data are weighted to match the UK adult population on age, gender, ethnicity and country.

[2] Participants stated that cancer was experienced in friends and family only and not in self.

symptom prevalence

During the past six months, 40.1% (3,025/7,543) of survey participants had experienced at least one potential cancer symptom **(Table 2)**. Of these, a median of two symptoms per participant was reported (range 1-15 symptoms), while 31.8% (961/3,025) experienced three or more symptoms. Nearly one third

For peer review only - http://bmjopen.bmj.com/site/about/guidelines.xhtml

of all participants had experienced at least one non-specific symptom (2,284, 30.3%), almost a fifth reported at least one red flag symptom (1,327, 17.6%), and at least one symptom possibly indicative of lung cancer (1,386, 18.4%). The prevalence of individual symptoms ranged from 21.3% (1,603) ('tired all the time') to 1.5% (114) ('coughing up blood'). Among those reporting that they were 'tired all the time', had 'a persistent cough' or 'shortness of breath', around half said the symptom pre-dated the pandemic (826/1,603, 51.5%, 219/444, 49.3% and 510/1,052, 48.5% respectively) **(Supplementary file Table S4).**

For peer review only

Table 2: Participants experiencing potential cancer symptoms and associated symptom help-seeking

Data are n (%) and unweighted unless otherwise stated

Potential cancer symptom	Had symptom[1]	Had symptom - weighted[2]	Did not contact GP in the last 6 months[3]	Did not contact GP in the last 12 months - USEFUL Study[4]	Contacted GP in the last 6 months[5]	Contacted GP in the last 12 months - USEFUL Study[4]
	n / 7,543 (%)	n / 7,543 (%)	n /S (%)	n (%)	n /S (%)	n (%)
**Non-specific symptom**						
A persistent change in bowel habits	541 (7.2)	525 (7.0)	267 (49.4)	682/1,323 (51.5)	254 (47.0)	641/1,323 (48.5)
A persistent change in bladder habits	450 (6.0)	414 (5.5)	216 (48.0)	-	227 (50.4)	-
Tired all the time	1,603 (21.3)	1,614 (21.4)	1,031 (64.3)	1,778/3,078 (57.8)	540 (33.7)	1,300/3,078 (42.2)
Persistent unexplained pain	662 (8.8)	646 (8.6)	286 (43.2)	-	361 (54.5)	-
**Non-specific/Red flag symptom**						
Unexplained weight loss	395 (5.2)	433 (5.7)	205 (51.9)	152/341 (44.6)	179 (45.3)	189/341 (55.4)
**Red flag symptom**						
A change in the appearance of a mole	391 (5.2)	402 (5.3)	229 (58.6)	-	157 (40.2)	-
An unexplained lump or swelling	422 (5.6)	418 (5.5)	173 (41.0)	-	239 (56.6)	-
Unexplained bleeding	267 (3.5)	291 (3.9)	115 (43.1)	-	143 (53.6)	-
A persistent difficulty swallowing	237 (3.1)	248 (3.3)	97 (40.9)	557/884 (63.0)	128 (54.0)	327/884 (37.0)
A sore that does not heal	291 (3.9)	297 (3.9)	146 (50.2)	-	128 (44.0)	-
**Red flag/Lung-specific symptom**						
Coughing up blood	114 (1.5)	127 (1.7)	35 (30.7)	31/91 (34.1)	67 (58.8)	60/91 (65.9)
**Lung-specific symptom**						
Shortness of breath	1,052 (13.9)	966 (12.8)	538 (51.1)	1,228/2,647 (46.4)	484 (46.0)	1,419/2,647 (53.6)
Persistent hoarseness	200 (2.7)	206 (2.7)	95 (47.5)	941/1,319 (71.3)	96 (48.0)	378/1,319 (28.7)
A persistent cough	444 (5.9)	401 (5.3)	209 (47.1)	1,088/2,189 (49.7)	230 (51.8)	1,101/2,189 (50.3)
A change in an existing cough	196 (2.6)	219 (2.9)	84 (42.9)	153/298 (51.3)	100 (51.0)	145/298 (48.7)
All potential cancer symptoms	***3,025 (40.1)[6]***	***2,909 (38.6)***	***1,355/3,025 (44.8)[7]***	***3,974/9,810 (40.5)***	***1,636/3,025 (54.1)[8]***	***5,836/9,810 (59.5)***
Non-specific symptom	***2,284 (30.3)[6]***	***2,261 (30.0)***				
Red flag symptom	***1,327 (17.6)[6]***	***1,310 (17.4)***				
Lung-specific symptom	***1,386 (18.4)[6]***	***1,289 (17.1)***				

n=number, n/S = number of respondents representing each symptom help-seeking behaviour/number of respondents who had this symptom.

For peer review only - http://bmjopen.bmj.com/site/about/guidelines.xhtml

[1] Denominator includes those who did not have a symptom and those who preferred not to say (around 1% of the sample). [2] All data are weighted to match the UK adult population on age, gender, ethnicity and country. [3] Includes participants who had not contacted the GP yet, but planned to. 'Did not contact GP' and 'Contacted GP' columns are mutually exclusive. Denominator includes participants who preferred not to say. [4] Comparator data for adults aged >50 years who did and did not contact the GP in the last 12 months (Hannaford et al., 2020). [5] A further breakdown of help-seeking intervals is in Supplementary file Table S4. [6] At least one potential cancer symptom reported. [7] Did not contact the GP for symptoms reported in the last 6 months. 'Did not contact GP' and 'Contacted GP' columns are mutually exclusive. Denominator also includes 34 (1.1%) who preferred not to say across all their symptoms. [8] Contacted the GP for at least one symptom in the last six months.

For peer review only

symptom help-seeking

Among 3,025 participants who experienced at least one potential cancer symptom, 44.8% (1,355/3,025) had not contacted the GP for any of their reported symptoms over a six month time frame, whereas 40.5% (3,974/9,810) had not contacted their GP over a 12-month time frame in the USEFUL study **(Table 2)**. A small proportion preferred not to say across all symptoms (1.1%). The proportion of participants not seeking help varied by symptom. A substantial proportion of participants had not sought help for red flag symptoms including coughing up blood (35/114, 30.7%), an unexplained lump or swelling (173/422, 41.0%) or a change in the appearance of a mole (229/391, 58.6%). Almost half of those who reported non-specific symptoms including 'a persistent change in bowel habits' (267/541, 49.4%) and 'a persistent change in bladder habits' (216/450, 48.0%) had not sought help from their GP, whilst a higher proportion (1,031/1,603, 64.3%) reporting being 'tired all the time' had not sought help. Around half of those experiencing lung-specific symptoms such as 'a persistent cough' (209/444, 47.1%) and 'shortness of breath' (538/1,052, 51.1%) had not sought help. A further breakdown of help-seeking according to recommended intervals is provided in **Supplementary file Table S4.**

As shown in **Table 2**, the proportion of participants who had not contacted their GP over a six month time frame appeared to be higher than USEFUL study data for individual symptoms over a twelve month time frame including 'tired all the time' (64.3% (1,031/1,603) in the current study versus 57.8% (1,778/3,078) in the USEFUL study), 'unexplained weight loss' (51.9% (205/395) versus 44.6% (152/341)) and to a lesser extent 'shortness of breath' (51.1% (538/1,052) versus 46.4% (1,228/2,647)). Proportions not seeking help for 'persistent change in bowel habits' (49.4% (267/541) versus 51.5% (682/1,323)) and 'persistent cough' (47.1% (209/444) versus 49.7% (1,088/2,189)) appeared comparable. The proportion of participants who had not contacted their GP in the current study appeared to be lower than USEFUL study data for 'persistent difficulty swallowing' (40.9% (97/237) versus 63.0% (557/884)), 'persistent hoarseness' (47.5% (95/200) versus 71.3% (941/1,319)), 'change in an existing cough' (42.9% (84/196) versus 51.3% (153/298)) and to a lesser extent 'coughing up blood' (30.7% (35/114) versus 34.1% (31/91)). It should be noted that relatively small numbers of participants in the current study reported experiencing the latter four symptoms.

correlates of symptom help-seeking

In unadjusted analyses, seeking help from the GP for at least one symptom was associated with former or current smoking, disability, experience of cancer (self), perceiving cancer as the cause of symptom(s) experienced, and reporting a greater number of potential cancer symptoms **(Table 3).**

For peer review only - http://bmjopen.bmj.com/site/about/guidelines.xhtml

Perceiving COVID-19 as the cause of symptom(s) was associated with lower odds of help-seeking. There were no other statistically significant unadjusted associations. After adjustment for other factors, disability, reporting more symptoms and not perceiving COVID-19 as the cause of symptom(s) experienced remained statistically significantly associated with higher odds of help-seeking.

help-seeking attitudes, barriers and prompts

Of the overall sample (n=7,543), around two thirds reported feeling safe from COVID-19 if they needed to attend an appointment at their GP practice (5,142, 68.2%) or hospital (4,613, 61.2%). Nearly three quarters (5,452, 72.3%) were worried about delays to cancer tests and investigations due to COVID-19.

The most frequently endorsed barriers to medical help-seeking in the overall sample were worry about wasting the healthcare professional's time (1,158, 15.4%), worry about putting extra strain on the NHS (954, 12.6%), not wanting to be seen as someone who makes a fuss (907, 12.0%), difficulty getting an appointment with a particular healthcare professional (774, 10.3%) and worry about catching coronavirus (721, 9.6%). Remote consulting was one of the least frequently endorsed barriers (361, 4.8%) **(Supplementary material Table S1)**. A median of one barrier (25th to 75th centile 1 to 2 barriers, range 0 to 14) was identified per participant.

For COVID-CAM survey participants (n=5,667) the main prompts to speaking to a medical professional were having a symptom that was bothersome (1,008, 17.8%), didn't go away (957, 16.9%), was painful (811, 14.3%) and unusual (706, 12.5%) and having a feeling that something wasn't right (721, 12.7%) **(Supplementary material Table S2)**.

For peer review only - http://bmjopen.bmj.com/site/about/guidelines.xhtml

For peer review only

Table 3: Unadjusted and adjusted logistic regression models for self-reported symptom help-seeking in participants who experienced at least one potential cancer symptom (n=3,025[1])

Data are n (%) and unweighted unless otherwise stated

	Did not contact GP[2] n=1,355	Contacted GP[2] n=1,636	Crude OR (95% CI)	Adjusted OR (95% CI) N=2,281
**Age (years) N=2,942**				
18-24	127 (46.4)	147 (53.6)	ref (1.0)	ref (1.0)
25-34	199 (49.7)	201 (50.3)	0.87 (0.64 to 1.19)	0.80 (0.52 to 1.24)
35-44	196 (45.7)	233 (54.3)	1.03 (0.76 to 1.39)	1.12 (0.73 to 1.71)
45-54	211 (47.1)	237 (52.9)	0.97 (0.72 to 1.31)	1.11 (0.72 to 1.70)
55-64	220 (45.2)	267 (54.8)	1.05 (0.78 to 1.41)	1.08 (0.70 to 1.67)
65-74	284 (41.8)	396 (58.2)	1.20 (0.91 to 1.60)	1.29 (0.83 to 2.00)
75+	97 (43.3)	127 (56.7)	1.13 (0.79 to 1.61)	1.20 (0.72 to 2.00)
p-value			0.261	0.321
**Gender N=2,978**				
Male	625 (43.7)	804 (56.3)	ref (1.0)	ref (1.0)
Female	727 (46.9)	822 (53.1)	0.88 (0.76 to 1.02)	0.99 (0.82 to 1.21)
p-value			0.080	0.951
**Ethnicity N=2,988**				
White	1,193 (45.0)	1,457 (55.0)	ref (1.0)	ref (1.0)
Ethnic minorities[3]	160 (47.3)	178 (52.7)	0.91 (0.73 to 1.14)	0.85 (0.61 to 1.18)
p-value			0.420	0.328
**Country N=2,971**				
England	854 (47.2)	955 (52.8)	ref (1.0)	ref (1.0)
Wales	405 (42.5)	549 (57.5)	1.21 (1.03 to 1.42)	1.23 (0.98 to 1.54)
Scotland	72 (43.1)	95 (56.9)	1.18 (0.86 to 1.62)	1.35 (0.90 to 2.02)
Northern Ireland	15 (36.6)	26 (63.4)	1.55 (0.82 to 2.95)	1.79 (0.62 to 5.20)
p-value			0.062	0.140
**Country/Region[4] N=2,971**				
Wales	405 (42.5)	549 (57.5)	ref (1.0)	
Scotland	72 (43.1)	95 (56.9)	0.97 (0.70 to 1.36)	

For peer review only - http://bmjopen.bmj.com/site/about/guidelines.xhtml

For peer review only

	Did not contact GP[2] n=1,355	Contacted GP[2] n=1,636	Crude OR (95% CI)	Adjusted OR (95% CI) N=2,281
Northern Ireland	15 (36.6)	26 (63.4)	1·28 (0.67 to 2.55)	
England:				
North East England	59 (53.6)	51 (46.4)	0.64 (0.43 to 0.95)	
North West England	109 (45.2)	132 (54.8)	0.89 (0.67 to 1.19)	
Yorkshire and Humberside	85 (47.0)	96 (53.0)	0.83 (0.61 to 1.15)	
East Midlands	72 (50.3)	71 (49.7)	0.73 (0.51 to 1.03)	
South East England	130 (45.8)	154 (54.2)	0.87 (0.67 to 1.14)	
East Anglia	72 (43.1)	95 (56.9)	0.97 (0.70 to 1.36)	
South West England	84 (48.0)	91 (52.0)	0.80 (0.58 to 1.10)	
West Midlands	96 (46.6)	110 (53.4)	0.85 (0.62 to 1.14)	
London	147 (48.7)	155 (51.3)	0.78 (0.60 to 1.01)	
p-value			0.379	
**Highest level of education N=2,934**				
Degree or higher degree	514 (47.2)	574 (52.8)	ref (1.0)	ref (1.0)
A-levels or further education	460 (46.2)	536 (53.8)	1.04 (0.88 to 1.24)	0.91 (0.73 to 1.14)
O levels/GCSEs	265 (42.3)	362 (57.7)	1.22 (1.00 to 1.49)	1.07 (0.83 to 1.39)
Still studying	16 (36.4)	28 (63.6)	1.57 (0.84 to 2.93)	1.21 (0.51 to 2.89)
No formal qualifications	73 (40.8)	106 (59.2)	1.30 (0.94 to 1.79)	0.77 (0.51 to 1.16)
p-value			0.127	0.494
**Smoking status N=2,948**				
Never smoked	595 (50.6)	580 (49.4)	ref (1.0)	ref (1.0)
Former smoker	436 (41.2)	623 (58.8)	1.47 (1.24 to 1.73)	1.16 (0.94 to 1.44)
Current smoker	302 (42.3)	412 (57.7)	1.40 (1.16 to 1.69)	1.03 (0.80 to 1.32)
p-value			<0.001	0.358
**Marital relationship N=2,976**				
Not married or cohabiting	516 (46.4)	597 (53.6)	ref (1.0)	ref (1.0)
Married or cohabiting	831 (44.6)	1,032 (55.4)	1.07 (0.92 to 1.25)	0.95 (0.78 to 1.16)
p-value			0.352	0.647
**Disability N=2,900**				
No	1,042 (50.7)	1,014 (49.3)	ref (1.0)	ref (1.0)

For peer review only - http://bmjopen.bmj.com/site/about/guidelines.xhtml

	Did not contact GP[2] n=1,355	Contacted GP[2] n=1,636	Crude OR (95% CI)	Adjusted OR (95% CI) N=2,281
Yes	281 (33.3)	563 (66.7)	2.06 (1.74 to 2.43)	1.38 (1.11 to 1.71)
p-value			<0.001	0.003
**Experience of cancer N=2,991**				
No	270 (50.3)	267 (49.7)	ref (1.0)	ref (1.0)
Yes, other (family and friends)[5]	974 (45.8)	1,154 (54.2)	1.20 (0.99 to 1.45)	1.09 (0.84 to 1.43)
Yes, self	111 (34.0)	215 (66.0)	1.96 (1.47 to 2.60)	1.12 (0.76 to 1.66)
p-value			<0.001	0.783
**Symptom attributed to cancer[6] N=2,990**				
Not cancer	1,342 (45.6)	1,601 (54.4)	ref (1.0)	ref (1.0)
Cancer	12 (25.5)	35 (74.5)	2.44 (1.26 to 4.73)	1.30 (0.56 to 3.04)
p-value			0.008	0.547
**Symptom attributed to COVID[6] N=2,990**				
Not COVID	1,214 (44.0)	1,547 (56.0)	ref (1.0)	ref (1.0)
COVID	140 (61.1)	89 (38.9)	0.50 (0.38 to 0.66)	0.36 (0.25 to 0.52)
p-value			<0.001	<0.001
**Number of barriers to help-seeking (0 to 17) N=2,991** Median (25th to 75th centiles)	1 (1 to 3)	1 (1 to 3)	1.04 (1.00 to 1.08)	0.97 (0.92 to 1.03)
p-value			0.057	0.315
**Confident that I would be safe from coronavirus if I needed to attend an appointment at a hospital N=2,645**				
Strongly agree	251 (44.7)	311 (55.3)	ref (1.0)	ref (1.0)
Somewhat agree	518 (44.3)	650 (55.7)	1.01 (0.83 to 1.24)	0.86 (0.64 to 1.15)
Somewhat disagree	268 (44.0)	341 (56.0)	1.03 (0.82 to 1.29)	0.74 (0.51 to 1.06)
Strongly disagree	149 (48.7)	157 (51.3)	0.85 (0.64 to 1.12)	0.58 (0.36 to 0.94)
p-value			0.547	0.150
**Confident that I would be safe from coronavirus if I needed to attend an appointment at my GP surgery N=2,692**				
Strongly agree	337 (47.3)	375 (52.7)	ref (1.0)	ref (1.0)

For peer review only - http://bmjopen.bmj.com/site/about/guidelines.xhtml

	Did not contact GP[2] n=1,355	Contacted GP[2] n=1,636	Crude OR (95% CI)	Adjusted OR (95% CI) N=2,281
Somewhat agree	545 (44.1)	690 (55.9)	1.14 (0.95 to 1.37)	1.21 (0.92 to 1.58)
Somewhat disagree	217 (41.1)	311 (58.9)	**1.29 (1.03 to 1.62)**	1.47 (1.02 to 2.12)
Strongly disagree	102 (47.0)	115 (53.0)	1.01 (0.75 to 1.37)	0.93 (0.55 to 1.56)
p-value			0.146	0.082
**Worried about delays to cancer tests and investigations caused by coronavirus N=2,720**				
Strongly agree	479 (43.8)	614 (56.2)	ref (1.0)	ref (1.0)
Somewhat agree	534 (46.2)	621 (53.8)	0.91 (0.77 to 1.07)	1.03 (0.83 to 1.25)
Somewhat disagree	126 (41.0)	181 (59.0)	1.12 (0.87 to 1.45)	1.18 (0.86 to 1.62)
Strongly disagree	77 (46.7)	88 (53.3)	0.89 (0.64 to 1.24)	0.97 (0.65 to 1.45)
p-value			0.340	0.762
**Cancer symptom recognition score (score 0 to 15) N=2,991** Median (25th to 75th centiles)	11 (8 to 14)	11 (8 to 14)	1.00 (0.99 to 1.02)	1.01 (0.99 to 1.04)
p-value			0.789	0.263
**Number of symptoms (maximum 15) N=2,991** Median (25th to 75th centiles)	1 (1 to 2)	2 (1 to 4)	1.62 (1.53 to 1.72)	1.68 (1.56 to 1.82)
p-value			<0.001	<0.001

OR = odds ratio; CI = confidence interval; Note: an odds ratio >1 indicates increased odds of help-seeking. [1] n=34 participants indicated that they prefer not to say across all symptoms and were excluded from the analysis. [2] Did not contact the GP for symptoms reported in the last 6 months / Contacted the GP for at least one symptom in the last six months. [3] Ethnicity groups combined for analysis due to small numbers: 'Mixed/multiple ethnic groups', 'Asian/Asian British', 'Black/African/Caribbean/Black British', 'Other ethnic group', 'Prefer not say'. [4] Not included in multivariable model due to collinearity with country. [5] Participants stated that cancer was experienced in friends and family only and not in self.

For peer review only - http://bmjopen.bmj.com/site/about/guidelines.xhtml

qualitative results

Thirty participants were interviewed post survey completion (September-November 2020). Just over half were male (n=17), had received a higher education qualification or degree (n=19), lived in Wales (n=25) and were from a White ethnic background (n=23). The average age was 55 years (range 26-76 years). Exemplary quotes are provided in **Table 4**. Codes and code definitions identified relating to the key themes presented on symptom experiences, fear of help-seeking and experiences of help-seeking are provided in Supplementary material **Table S5**.

symptom experiences

Many participants reported noticing a change to their health or wellbeing during the six months from the start of the first UK lockdown. This was commonly attributed to changes in existing health conditions such as asthma or diabetes or side-effects of medication. This was more notable for non-specific symptoms such as tiredness all the time. As a result, participants delayed their help-seeking, or did not seek help at all, to avoid bothering the doctor when they assumed that they already knew the cause. Even when participants reported red flag symptoms, there was discussion of delaying due to concerns about the NHS being over-stretched. Several participants described accessing other services as a way of easing pressures on their GP practice, for example by phoning 111 or contacting their pharmacist. When making decisions about help-seeking, participants weighed the risks of their clinical need against the risks of catching or exposing others to COVID-19 and burdening the NHS. Some participants conveyed the sentiment that the least they could do to help was to stay away from the NHS.

fear of help-seeking

All participants expressed fear or nervousness about presenting to primary or secondary care. For some, levels of fear were very high. This was commonly associated with 'the unknown' and potentially encountering other members of the public who may not adhere to social distancing guidance. These acted as barriers to timely medical help-seeking. Changes to GP practice procedures invoked worry and hesitancy due to not knowing or understanding the new measures. Examples included the use of new online and telephone triage systems and one-way systems in medical buildings. Participants understood the need for these adaptations, though felt that more support could be provided on how to navigate these changes. Participants expressed particular concern for patients with low digital literacy and those with English as a second language or additional mobility needs.

Fear of attending secondary care was acute for many. Some participants reported being too scared to attend secondary care appointments, treatments or procedures. They made this decision knowing that it

For peer review only - http://bmjopen.bmj.com/site/about/guidelines.xhtml

could be detrimental to their health and wellbeing. However, those who did attend face-to-face in primary and/or secondary care described feeling ‘safe’ and ‘secure’ when attending. Participants expressed surprise that attending was at odds with their expectations of what it was going to be like. Participants described viewing ‘scaremongering’ media reports of hospitals being over-run with coronavirus cases exacerbating their fears. Several participants were saddened that they had been manipulated by the media into feeling scared and avoiding healthcare, with consequences for their health.

experiences of help-seeking

When participants had contacted their GP, overall they were pleased with the quality of care received and the use of remote consultations. Some were hesitant about disclosing details of their health and medical history before a decision was made about whether they could speak to or see a doctor, feeling that this impacted on their privacy. The use of telephone consultations was praised by most who had received them. Many of these participants reported that it was easier and faster to get a GP appointment than before the pandemic, and that they would like to keep the change to remote consulting on the understanding that face-to-face appointments would be available based on clinical need.

Table 4: Exemplary participant quotes by major theme for symptom experiences, fear of help-seeking and experiences of help-seeking

**Major Theme**	**Exemplary Participant Quotes** (participant ID, gender, age (years), nation of residency) [Quotes provided in intelligent verbatim; P = Participant, I = Interviewer]
**Symptom experiences**	“P: No, apart from the return of the backache … but I think I know why that is, so I haven't done anything about it. Because I know what's going to help it, so as soon as I can go back to the gym, or decide to go back to the gym and start those classes, it will be fine.” *(64021806, Female, 64, Wales)*
	“P: I noticed I was getting increasingly tired… I had a couple of other symptoms as well, which made me think my Levothyroxine dose was now insufficient” *(63984720, Male, 62, Wales)*
	“I: Okay and has the pandemic affected or changed how you think about doctors’ visits and appointments at all?<br>P: I would certainly said I’ve been more reluctant, I would have stayed away and just dealt with it, rather than perhaps going to see a doctor at an early stage.” *(64948240, Female, 46, Wales)*
	“P: ... over the weekend I had a, second time in my life, a bad migraine, and thankfully I'm feeling better but I had thought to myself at what point am I going to go to the GP about not feeling better. And will I ... you know am I less likely to go because they're under strain? And I probably am a bit less likely to go, delay it a little bit longer” *(64078317, Female, 46, England)*

For peer review only - http://bmjopen.bmj.com/site/about/guidelines.xhtml

	"P: … it's certainly changed my mind because like I say I'm of the mindset that says if it's not sort of life threatening critical then, you know, it can wait. So yes, you know I had a certainly different mentality and part of that I think is because of the strain that was put on the health service and all those within it initially that you perhaps didn't want to disturb them" *(65205685, Female, 63, Wales)*
**Fear of help-seeking**	"P: … I haven't been there, the last time I went there, I think it was in the January when I had my annual COPD and CHD review… So, I hadn't been there since, and then I was reading all these horror stories, you know, the stuff we were seeing on the telly. You know the people were going into places, and they didn't even know they had the virus, they wasn't showing symptoms… And passing it on and I was thinking, this could happen to me in the doctor's surgery, but when I actually went to the surgery the whole layout had changed, it had all new furniture put in there, so it could be wiped down." *(65205685, Female, 63, Wales)*
	"P: Well if you're asking about hospital, I was supposed to go to hospital in lockdown see, but the thing is, I was too frightened because of Covid, I thought I'm not going to hospital. And I needed stitches in my knee, because I fell and I landed on both knees in the living room, I fell over the mat. I sliced my knee open, and I needed stitches bad, but I didn't go. My husband used butterfly stitches and done it that way. But I wouldn't go because of Covid see, because I was too frightened, because I didn't want to get Covid." *(64018114, Female, 44, Wales)*
	"P: … I mean my view to hospitals, prior to being in one myself, was that, you know there were people dying all over the place in every ward, every corridor with coronavirus. So yes, I would have been, as I say, certainly very cautious to have, to have wanted to put myself in that situation…. you know I was so impressed with how the hospital were operating when I was in there and, as I say if I'd had vision or understood what it was looking like, how it was working I probably wouldn't have had any concerns at all. I think the hospitals were the safest, safest place to be, is my view after the event, seeing how fantastically well the staff were, you know at following procedure etc… So yes if, you know, if you get that message across that, that a hospital, as I say, is probably the safest place than bloody Tesco's or the local pub or whatever. You know, you're very safe there." *(65205685, Female, 63, Wales)*
**Experiences of help-seeking**	"P: … the surgery did a triage thing, the doctor called me and asked me to go and see them and that worked okay, you know, under the restrictions of the local GP, surgery, you know… They have, they've got, quite stringent processes… Yeah, I was content there, no serious misgivings, you accept their protocols and the new way of doing things and that was fine actually, no problem." *(64026131, Male, 62, Wales)*
	"P: Like I said that assumption a lot of people make as well… They assume that because you're okay, you're seeing them in real life, you're okay talking to them over the video, like I said I, I really don't feel comfortable using those video things. I can't sort of speak normally over them. I feel very disconnected from the person I just, I find it really hard to do." *(64027453, Male, 38, Wales)*
	"P: It has changed the whole system, you can't just make an appointment to go and see somebody, you have to go online, type in briefly what your problem is and then decide whether they call you back or whether they tell you what to do or whether they say I think we should meet face to face. Usually a telephone conversation first and then decide okay perhaps you'd better come down and see me. Which I did once… I think the system works very well actually.<br>I: Do you, so how does it compare then before the pandemic? Could you just make an appointment in those?

For peer review only - http://bmjopen.bmj.com/site/about/guidelines.xhtml

	P: You could but it was always sort of three or four weeks ahead… With the new system, you seem to get some response within the next twenty-four hours which is a big improvement.” *(63986310, Male, 76, Wales)*

DISCUSSION:

We conducted the first population study of cancer symptom experience and help-seeking behaviour during the COVID-19 in the United Kingdom. Among adults surveyed who experienced one or more potential cancer symptom during the first six months of the pandemic, nearly half had not sought help for any symptoms from the GP during this time, even for red flag symptoms. Reporting a disability and experiencing more symptoms were associated with higher odds of symptom help-seeking, whereas attributing a symptom(s) to COVID-19 was associated with lower odds. Qualitative data revealed reluctance to contact primary care services due to concerns about catching or transmitting coronavirus and overburdening the NHS. Interviewees described delaying medical help-seeking due to fears that were driven by and exacerbated by media reports of COVID-19 in hospitals.

The prevalence of symptoms experienced over the six month period in the current study was in line with previous studies.[13,34] Symptom help-seeking behaviour during the first six months of the pandemic appeared to be lower than help-seeking reported in the USEFUL study over a twelve month time frame, overall and for individual symptoms such as persistent tiredness and unexplained weight loss, although direct comparison was restricted by methodological differences such as variation in symptom reporting time frames. Similarly to previous research, key help-seeking barriers in the current study included worry about wasting healthcare professionals’ time, over-stretching limited healthcare resources and accessing healthcare services (personal communication).[28,35] In a Spanish population sample, Petrova et al.[36] also reported barriers to anticipated symptom help-seeking during the COVID-19 pandemic including worry about wasting the doctor’s time and worry about what the doctor might find. International pre-pandemic research on barriers to help-seeking has found that UK adults are more likely to report worry about ‘bothering the doctor’ compared to those in other high-income countries.[16] Participants in our study described putting their health concerns on hold or self-managing conditions and concerns to avoid burdening the NHS, suggesting a compounding of the ‘British stiff upper lip’ phenomenon observed in pre-pandemic research.[16] Novel COVID-specific barriers and attitudes reflecting concerns about COVID-19 infection in healthcare settings and delayed cancer testing were prevalent in both the current survey and qualitative interviews, but they did not contribute significantly to modelling help-seeking behaviour. Difficulty with remote healthcare consulting was not frequently endorsed; indeed, qualitative findings suggested that when participants had contacted their GP or visited hospital, they reported positive

For peer review only - http://bmjopen.bmj.com/site/about/guidelines.xhtml

experiences that contrasted with their expectations. Retaining remote consultations alongside face-to-face consultations in future routine healthcare services was favoured.

The correlates of help-seeking behaviour in this study in part reinforce what has been observed in previous studies. The influence of disability and reporting more symptoms on help-seeking behaviour aligns with previous studies including Hannaford et al.[13] in which people who were unable to work due to illness or disability were more likely to act on their symptoms. Mechanisms which serve to both increase and decrease timely presentation for symptoms have been previously identified and may vary by nature of comorbidity.[17,37] This relationship was observed in our qualitative interviews whereby participants who experienced a new or changing symptom attributed such changes to pre-existing conditions or medications, although results show this did not deter help-seeking in statistical analyses. In contrast, attributing symptoms to COVID-19 was associated with not contacting the GP and may have been influenced by government messaging to stay at home if experiencing any COVID-like symptoms. The decision not to act on symptoms experienced during the first UK pandemic wave may have been motivated by a desire to protect others in the community from COVID-19 infection, and to prevent healthcare services from being overwhelmed. A qualitative study of GPs' perceptions of changes in symptom help-seeking behaviour described patients as more vigilant about their health but also more reluctant to seek help as a result of the pandemic.[38] Our finding that current and former smokers were more likely to seek help was similar to findings reported in Hannaford et al.'s USEFUL study.[13] Although the association did not remain after adjustment in the present study, the consistency of this emerging finding with Hannaford and colleagues warrants investigation in future research. It is possible, for example, that people who currently smoke or have previously smoked perceive an elevated personal risk status which may prompt symptom presentation. The total number of help-seeking barriers endorsed was not associated with help-seeking behaviour, and more fine-grained analysis of differentiated emotional, practical or service-related barriers is needed.

A key strength of our study was the focus on actual symptoms experienced during the last six months. This reduced the known biases associated with retrospective recall of actual symptoms in patient samples or anticipated responses to hypothetical symptoms in community samples. Pooling data across two surveys provided a large sample that was broadly representative of the British population. However, we acknowledge that willingness and ability to complete an online survey was a prerequisite of study participation that may limit the generalisability of findings. Further methodological limitations are recognised, including the likelihood of reduced sample variation. Despite good representation of ethnic minority groups and people with lower education due to

For peer review only

targeted recruitment, we did not observe differences in help-seeking previously identified among these groups.[25,30] This may reflect reduced statistical power to detect such effects because we restricted the analysis to actual symptom-helping among those who had experienced at least one potential cancer symptom. Further research is warranted to examine patterns of help-seeking for individual symptoms or subsets of symptoms (e.g. respiratory) and receptiveness to remote GP consulting among participants with varying degrees of digital literacy and health motivation. We acknowledge the constraints on our ability to compare rates of symptom help-seeking during the pandemic with those reported pre-pandemic, due to methodological differences including the longer symptom reporting time frame (twelve months) and older age inclusion criteria (>50 years) in the USEFUL study comparator. However, our qualitative findings indicate that people were not coming forward to their GP with symptoms during the first six months of the pandemic. The statistical modelling also showed that attributing symptoms to COVID-19 was associated with lower odds of help-seeking. This pattern may have contributed to the decline in GP referrals for suspected cancer that was observed during 2020.[6]

Evidence from this study highlights the need for continued investment in evidence-led, nationally funded and coordinated cancer awareness campaigns to legitimise seeking help for unusual or persistent symptoms. Clear, consistent information from a trusted source should encourage confidence in contacting the GP promptly, explain the changes to GP practice procedures and what to expect, and alleviate worries about health service capacity and infection control in hospital settings. Credible patient stories with an emphasis on positive outcomes could be important in counteracting possible hyperbolic COVID-19 news reporting and to appropriately recontextualise accounts and support engagement with hospital outpatient appointments, treatments or investigations. Campaigns and other supporting activity could increase uptake and access to remote consulting as it becomes embedded in primary and secondary cancer care.[39] Evaluation of campaign activity and other interventions is essential to ensure that they reach diverse audiences and do not exacerbate inequalities. As the COVID-19 pandemic continues, research must continue to monitor the influences on help-seeking for potential cancer symptoms.

For peer review only - http://bmjopen.bmj.com/site/about/guidelines.xhtml

ACKNOWLEDGEMENTS:

This study was facilitated by HealthWise Wales, the Health and Care Research Wales initiative, which is led by Cardiff University in collaboration with SAIL, Swansea University. This research comes under the auspices of the Health and Care Research Wales funded Primary and Emergency Care Research Centre (PRIME) [517195] and Wales Cancer Research Centre [517190]. DECIPHer and The Centre for Trials Research receive funding from Health and Care Research Wales and Health and Care Research Wales and Cancer Research UK. JW is funded by a Cancer Research UK Career Development Fellowship (C7492/A17219). We are grateful to Cancer Research UK's Cancer Insights Patient Panel, Clinical Advisory Panel and GP Panel and PRIME Centre Wales SUPER Group for their helpful feedback. Cancer Research UK staff members and other researchers have contributed to the initiation and development of CAM surveys, both historically and for this project. COVID-CAM data was provided by Cancer Research UK who collected the data via Dynata's online survey panels. External Scientific Advisory Group members who advised the statistical analysis plan include: Professor Jamie Brown (University College London), Professor Yoryos Lyratzopoulos (University College London), Dr Katie Robb (University of Glasgow), Dr Christian von Wagner (University College London) and Professor Fiona Walter (University of Cambridge).

COMPETING INTERESTS:

The authors declare no conflict of interest.

FUNDING:

This work was supported by Economic and Social Research Council as part of UK Research and Innovation's Rapid Response to COVID-19 grant number ES/V00591X/1. The funders had no role in the design, conduct or analyses of this study.

AUTHOR CONTRIBUTIONS:

KB (responsible for funding acquisition), RC-J, JT, KLW, JW, HQ-S, KO, GM, MR, JHep, AG contributed to the study design, protocol development, study management and planning. JHep provided lay input to the study design, study implementation and interpretation of the results. All MG built the online survey tool and managed the data for the COVID-19 Cancer Help-Seeking and Behaviour Study. VW managed the CRUK COVID-CAM. YM is the CABS study manager. DG and RC-J verified the data and carried out the statistical analysis. YM, HQ-S, JHug and GMc collected and analysed the qualitative data. authors reviewed the data analyses, contributed to data interpretation and writing of the manuscript, and approved the final version of the submitted manuscript.

For peer review only

ETHICAL APPROVAL AND CONSENT TO PARTICIPATE:

Ethical approval was granted by the School of Medicine Research Ethics Committee, Cardiff University (ref 20.68). Informed consent was provided from all participants at recruitment. This study was conducted in accordance with Good Clinical Practice and the Declaration of Helsinki.

CONSENT FOR PUBLICATION:

Not applicable

DATA SHARING:

De-identified participant data will be made available to the scientific community with as few restrictions as feasible, whilst retaining exclusive use until the publication of major outputs. Data will be available on a public archive.

For peer review only

For peer review only - http://bmjopen.bmj.com/site/about/guidelines.xhtml

REFERENCES:

1 Cancer Research UK. Cancer mortality for all cancers combined. CancerStats. 2019.http://www.cancerresearchuk.org/cancer-info/cancerstats/mortality/all-cancers-combined/ (accessed 21 Jan 2021).

2 Ferlay J, Colombet M, Soerjomataram I, *et al.* Estimating the global cancer incidence and mortality in 2018: GLOBOCAN sources and methods. *Int J Cancer* 2019;**144**:1941–53. doi:https://doi.org/10.1002/ijc.31937

3 Swann R, McPhail S, Witt J, *et al.* Diagnosing cancer in primary care: results from the National Cancer Diagnosis Audit. *Br J Gen Pract* 2018;**68**:e63 LP-e72. doi:10.3399/bjgp17X694169

4 Neal RD, Tharmanathan P, France B, *et al.* Is increased time to diagnosis and treatment in symptomatic cancer associated with poorer outcomes? Systematic review. *Br J Cancer* 2015;**112**:S92–107. doi:10.1038/bjc.2015.48

5 Koo MM, Swann R, McPhail S, *et al.* Presenting symptoms of cancer and stage at diagnosis: evidence from a cross-sectional, population-based study. *Lancet Oncol* 2020;**21**:73–9. doi:10.1016/S1470-2045(19)30595-9

6 Appointments in General Practice September 2019 - NHS Digital. https://digital.nhs.uk/data-and-information/publications/statistical/appointments-in-general-practice/september-2019 (accessed 12 May 2021).

7 Maringe C, Spicer J, Morris M, *et al.* The impact of the COVID-19 pandemic on cancer deaths due to delays in diagnosis in England, UK: a national, population-based, modelling study. *Lancet Oncol* 2020;**21**:1023–34. doi:10.1016/S1470-2045(20)30388-0

8 Hiom S. How coronavirus is impacting cancer services in the UK. Cancer Res. UK. 2020.https://scienceblog.cancerresearchuk.org/2020/04/21/how-coronavirus-is-impacting-cancer-services-in-the-uk/

9 Jones D, Neal RD, Duffy SRG, *et al.* Impact of the COVID-19 pandemic on the symptomatic diagnosis of cancer: the view from primary care. *Lancet Oncol* 2020;**21**:748–50. doi:10.1016/S1470-2045(20)30242-4

10 Weller D, Vedsted P, Rubin G, *et al.* The Aarhus statement: improving design and reporting of studies on early cancer diagnosis. *Br J Cancer* 2012;**106**:1262–7. doi:10.1038/bjc.2012.68

11 Walter F, Webster A, Scott S, *et al.* The Andersen Model of Total Patient Delay: a systematic review of its application in cancer diagnosis. *J Health Serv Res Policy* 2012;**17**:110–8. doi:10.1258/jhsrp.2011.010113

12 Scott SE, Walter FM, Webster A, *et al.* The Model of Pathways to Treatment: Conceptualization and integration with existing theory. *Br J Health Psychol* 2013;**18**:45–65.

For peer review only - http://bmjopen.bmj.com/site/about/guidelines.xhtml

doi:https://doi.org/10.1111/j.2044-8287.2012.02077.x

13 Hannaford PC, Thornton AJ, Murchie P et al. Patterns of symptoms possibly indicative of cancer and associated help-seeking behaviour in a large sample of United Kingdom residents—The USEFUL study. *PLoS One* 2020;**15**.https://doi.org/10.1371/journal.pone.0228033

14 Whitaker KL, Smith CF, Winstanley K, *et al.* What prompts help-seeking for cancer 'alarm' symptoms? A primary care based survey. *Br J Cancer* 2016;**114**:334–9. doi:10.1038/bjc.2015.445

15 Whitaker KL, Scott SE, Winstanley K et al. Attributions of Cancer 'Alarm' Symptoms in a Community Sample. *PLoS One* 2014;**9**:1–17.https://doi.org/10.1371/journal.pone.0114028No

16 Forbes LJL, Simon AE, Warburton F, *et al.* Differences in cancer awareness and beliefs between Australia, Canada, Denmark, Norway, Sweden and the UK (the International Cancer Benchmarking Partnership): do they contribute to differences in cancer survival? *Br J Cancer* 2013;**108**:292–300. doi:10.1038/bjc.2012.542

17 Birt L, Hall N, Emery J, *et al.* Responding to symptoms suggestive of lung cancer: a qualitative interview study. *BMJ Open Respir Res* 2014;**1**:e000067. doi:10.1136/bmjresp-2014-000067

18 de Joode K, Dumoulin DW, Engelen V, *et al.* Impact of the coronavirus disease 2019 pandemic on cancer treatment: the patients' perspective. *Eur J Cancer* 2020;**136**:132–9. doi:https://doi.org/10.1016/j.ejca.2020.06.019

19 Helsper CW, Campbell C, Emery J, *et al.* Cancer has not gone away: A primary care perspective to support a balanced approach for timely cancer diagnosis during COVID-19. *Eur J Cancer Care (Engl)* 2020;**29**:e13290–e13290. doi:10.1111/ecc.13290

20 Leventhal H, Brisette I, Leventhal E. *The common sense model of self regulation of health and illness In: CameronL, Leventhal E, eds. The self-regulation of health and illness behaviour.* 2003.

21 CABs protocol. https://osf.io/zxyp3 (accessed 2 Feb 2021).

22 Kelley K, Clark B, Brown V, *et al.* Good practice in the conduct and reporting of survey research. *Int J Qual Heal Care* 2003;**15**:261–6. doi:10.1093/intqhc/mzg031

23 von Elm E, Altman DG, Egger M, *et al.* The Strengthening the Reporting of Observational Studies in Epidemiology (STROBE) Statement: Guidelines for Reporting Observational Studies. *Epidemiology* 2007;**18**.https://journals.lww.com/epidem/Fulltext/2007/11000/The_Strengthening_the_Reporting_of_Observational.27.aspx

24 Stubbings S, Robb K, Waller J, *et al.* Development of a measurement tool to assess public

For peer review only - http://bmjopen.bmj.com/site/about/guidelines.xhtml

awareness of cancer. *Br J Cancer* 2009;**101**:S13–7. doi:10.1038/sj.bjc.6605385

25 CAM 2019. https://osf.io/j67dt/ (accessed 2 Feb 2021).

26 HealthWise Wales. https://www.healthwisewales.gov.wales (accessed 2 Feb 2021).

27 Berinsky AJ, Margolis MF, Sances MW. Separating the shirkers from the workers? Making sure respondents pay attention on self-administered surveys. *Am J Pol Sci* 2014;**58**:739–53. doi:10.1111/ajps.12081

28 Connor, K, Hudson, B, Power E. Awareness of the Signs, Symptoms, and Risk Factors of Cancer and the Barriers to Seeking Help in the UK: Comparison of Survey Data Collected Online and Face-to-Face. *JMIR Cancer* 2020;**6**. doi:10.2196/14539

29 Carletta J. Assessing agreement on classification tasks: the kappa statistic. *CoRR* 1996;**cmp-lg/960**.http://arxiv.org/abs/cmp-lg/9602004

30 Cancer Research UK - COVID - Survey - Confidence to attend appointments and tests at primary and secondary care settings. https://osf.io/km4ry/ (accessed 2 Feb 2021).

31 Braun V, Clarke V. Using thematic analysis in psychology., 3:2 (2006), 77-101. *Qual Res Psychol* 2006;**3**:77–101. doi:10.1191/1478088706qp063oa

32 Riley RD, Ensor J, Snell KIE H et al. Calculating the sample size required for developing a clinical prediction model. *BMJ* 2020;**368**. doi:10.1136/bmj.m441

33 Svendsen RP, Støvring H, Hansen BL, *et al.* Prevalence of cancer alarm symptoms: A population-based cross-sectional study. *Scand J Prim Health Care* Published Online First: 2010. doi:10.3109/02813432.2010.505412

34 Salika T, Lyratzopoulos G, Whitaker KL, *et al.* Do comorbidities influence help-seeking for cancer alarm symptoms? A population-based survey in England. *J Public Health (Bangkok)* 2018;**40**:340–9. doi:10.1093/pubmed/fdx072

35 Niksic M, Rachet B, Warburton FG, *et al.* Cancer symptom awareness and barriers to symptomatic presentation in England—are we clear on cancer? *Br J Cancer* 2015;**113**:533–42. doi:10.1038/bjc.2015.164

36 Petrova D, Pollán M, Rodriguez-Barranco M, *et al.* Anticipated help-seeking for cancer symptoms before and after the coronavirus pandemic: results from the Onco-barometer population survey in Spain. *Br J Cancer* 2021;**124**:2017–25. doi:10.1038/s41416-021-01382-1

37 Renzi C, Kaushal A, Emery J, *et al.* Comorbid chronic diseases and cancer diagnosis: disease-specific effects and underlying mechanisms. *Nat Rev Clin Oncol* 2019;**16**:746–61. doi:10.1038/s41571-019-0249-6

38 Archer S, Calanzani N, Honey S, *et al.* Impact of the COVID-19 pandemic on cancer assessment in primary care: a qualitative study of GP views. *BJGP Open*

For peer review only

For peer review only - http://bmjopen.bmj.com/site/about/guidelines.xhtml

2021;:BJGPO.2021.0056. doi:10.3399/bjgpo.2021.0056

39 Broom A, Kenny K, Page A, *et al.* The Paradoxical Effects of COVID-19 on Cancer Care: Current Context and Potential Lasting Impacts. *Clin Cancer Res* 2020;**26**:5809 LP – 5813. doi:10.1158/1078-0432.CCR-20-2989

For peer review only

FIGURE LEGEND:

Figure 1: Recruitment flow chart

For peer review only

For peer review only - http://bmjopen.bmj.com/site/about/guidelines.xhtml

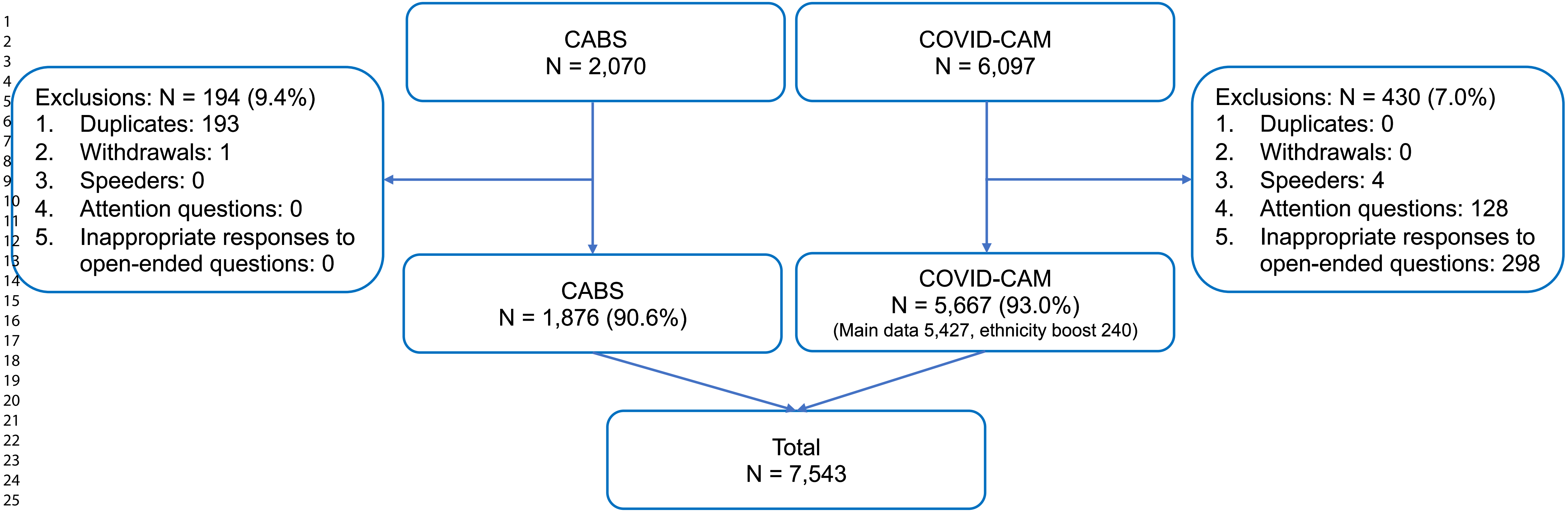

inclusion criteria:
All participants ≥18 years of age and living in the United Kingdom

Exclusion criteria:
1. Duplicates:
   - CABS: based on first name, surname and DOB - first entry retained
   - COVID-CAM: not applicable - participants are prevented from taking the survey multiple times
2. Withdrawals: Participants are excluded if they request withdrawal at any stage
3. Speeders: Completion of the survey <30% of the median time taken
4. Attention questions:
   - CABS: Failed both attention questions and not due to confusion/humour/sarcasm
   - COVID-CAM: failed at least one attention question
5. Inappropriate responses to open-ended questions: manual assessment if free-text were nonsensical/offensive

For peer review only - http://bmjopen.bmj.com/site/about/guidelines.xhtml

Supplementary File

Table S1: Barriers to consulting with a medical professional

Data are n (%) and unweighted unless otherwise stated

Barriers[1]	Pooled sample N=7,543	Pooled sample Weighted[2] N=7,543	At least one symptom experienced N=3,025	No symptoms experienced[3] n=4,428
I worried about wasting the healthcare professional's time	1,158 (15.4)	930 (12.3)	653 (21.6)	505 (11.4)
I worried about putting extra strain on the NHS	954 (12.6)	790 (10.5)	578 (19.1)	376 (8.5)
I didn't want to be seen as someone who makes a fuss	907 (12.0)	856 (11.3)	540 (17.9)	367 (8.3)
I found it difficult to get an appointment with a particular healthcare professional	774 (10.3)	627 (8.3)	448 (14.8)	326 (7.4)
I worried about catching coronavirus	721 (9.6)	632 (8.4)	415 (13.7)	306 (6.9)
I found it difficult to get an appointment at a convenient time	643 (8.5)	659 (8.7)	321 (10.6)	322 (7.3)
I worried they wouldn't take my symptom(s) seriously	601 (8.0)	574 (7.6)	380 (12.6)	221 (5.0)
I didn't want to talk to a receptionist/administrative person about my symptom(s)	518 (6.9)	458 (6.1)	304 (10.0)	214 (4.8)
I worried about what they might find wrong with me	421 (5.6)	452 (6.0)	231 (7.6)	190 (4.3)
I had too many other things to worry about	401 (5.3)	434 (5.8)	271 (9.0)	130 (2.9)
It would have been difficult for me to discuss my health problem remotely (by phone, email or video call)	361 (4.8)	319 (4.2)	231 (7.6)	130 (2.9)
I found it embarrassing talking about my symptoms	354 (4.7)	384 (5.1)	216 (7.1)	138 (3.1)
I was too busy to make time to seek medical attention	329 (4.4)	354 (4.7)	195 (6.4)	134 (30)
I worried about the possibility of having treatment	304 (4.0)	318 (4.2)	196 (6.5)	108 (2.4)
I didn't feel confident talking about my symptom(s)	272 (3.6)	309 (4.1)	160 (5.3)	112 (2.5)
I worried about the impact on my employment from taking time off	227 (3.0)	252 (3.3)	144 (4.8)	83 (1.9)
I had symptoms that might have been related to coronavirus	143 (1.9)	153 (2.0)	105 (3.5)	38 (0.9)
Nothing put me off/delayed me in seeking medical attention	3,039 (40.3)	2,845 (37.7)	859 (28.4)	2,180 (49.2)
Prefer not to say	114 (1.5)	130 (1.7)	31 (1.0)	83 (1.9)
**Number of barriers to help-seeking reported (0 to 17)**				
Median (25th to 75th centiles); Range	1.0 (1.0 to 2.0); (0 to 14)			

For peer review only - http://bmjopen.bmj.com/site/about/guidelines.xhtml

[1] Participants were asked: *"Thinking about the last time you considered seeing or speaking to a medical professional about your health, did any of the following put you off, or make you delay doing so? (This may have been an appointment with a medical professional (e.g. a doctor, nurse or pharmacist) in person, online or over the phone). Please select all that apply".* More than one barrier could be selected. Numbers do not amount to the denominator and percentages do not amount to 100%. [2] All data are weighted to match the adult population in the UK on age, gender, ethnicity, and region. [3] Includes those who preferred not to say.

For peer review only

Table S2. Help-seeking prompts to consulting with a medical professional *Data are n (%) and unweighted unless otherwise stated*

	COVID-CAM N=5,667	COVID-CAM Weighted[1] N=5,667
I had a symptom that I thought might be a sign of cancer	282 (5.0)	286 (5.0)
I had a symptom that was unusual for me	706 (12.5)	709 (12.5)
I had a symptom that was painful	811 (14.3)	815 (14.4)
I knew someone who had a similar symptom, and it turned out to be serious	122 (2.1)	119 (2.1)
I had a symptom that didn't go away	957 (16.9)	970 (17.1)
My friends or family encouraged me to go	461 (8.1)	454 (8.0)
I had a symptom, but I didn't know what was causing it	683 (12.1)	686 (12.1)
I had a symptom that was 'bothersome'	1,008 (17.8)	1,005 (17.7)
I had a feeling that something wasn't right	721 (12.7)	735 (13.0)
I had seen information about this symptom in the media	137 (2.4)	139 (2.5)
I could have a remote consultation (for example, by phone, email or video call)	448 (7.9)	447 (7.9)
I needed an appointment for a pre-existing problem/condition[2]	687 (12.1)	680 (12.0)
I needed help for a specific symptom or injury[2]	67 (1.2)	70 (1.2)
I needed a women's health appointment[2]	36 (0.6)	39 (0.7)
I had a symptom that was getting worse[2]	7 (0.1)	8 (0.1)
I needed to have a lab test or get a test result[2]	8 (0.1)	9 (0.2)
Other[3]	144 (2.5)	142 (2.58)
I have never sought medical attention	307 (5.4)	291 (5.1)
I don't remember	798 (14.1)	811 (14.3)
Prefer not to say	150 (2.6)	149 (2.6)

More than one **prompt** could be selected. Numbers do not amount to the denominator and percentages do not amount to 100%.

[1] All data are weighted to match the adult population in the UK on age, gender, ethnicity, and region. [2] Recoded from the 'other' free text option. [3] Reasons that could not be categorised into cohesive themes.

For peer review only - http://bmjopen.bmj.com/site/about/guidelines.xhtml

S3 - Interview Topic Guide

Participants will have access to study Information sheets and will have already provided informed consent. Prior to interview commencement the interviewer will re-confirm verbal consent.

The interview will be recorded, anonymised, transcribed confidentially and analysed by members of the research team.

The aim of the interview is to gain further understanding of how participants perceive symptoms, help seeking and behaviour regarding potential cancer symptoms during the COVID-19 lockdown from March 23rd 2020.

It is estimated the interview will be 45 minutes in length. Following the interview participants will be sent a £20 voucher to thank them for their time.

The following is a semi-structured topic guide. This is not a script - please feel free to amend wording/order and probe further on interesting points/topic where appropriate. Please also skip redundant questions based on previous answers where appropriate.

Topic Guide

General Health/Introduction

- How are you feeling today and generally?
- Has the pandemic and the lockdown affected your beliefs or feelings or concerns about your own health?
- Have you changed anything in your usual habits (eating, exercising, smoking) due to the coronavirus pandemic and lockdown?

Symptoms and Help seeking

- Since the start of the pandemic and UK lockdown in March this year, have you noticed any changes in your health/body?
  - Discuss decision to see/not see GP if appropriate
  - Discuss what their changes in their health/body were and what they did if appropriate
  - Discuss whether they were concerned about the changes if appropriate
  - Discuss whether they spoke to anyone about the changes
- How important do you think it is to talk to a doctor or healthcare professional if you (were to) notice a change in your health at the moment?
  - Discuss whether the pandemic has affected/changed this at all
- Did you have any concerns about attending a GP appointment in person during the pandemic?
- What do you think would encourage people to contact their GP to check on change to their health or body?
- If you did speak to your GP or a healthcare professioanl during the pandemic, how did you talk to them?
  - Discuss how people felt about face-to-face appointments or virtual/remote consultations at the moment, whether it worked for them and whether they felt satisfied
- What are your thoughts about phone or video (remote) consulting?
- How did getting help or advice from your GP or a healthcare professional compare to what you experienced before?

For peer review only - http://bmjopen.bmj.com/site/about/guidelines.xhtml

- If your GP or healthcare professional referred you to the hospital, how would you feel about attending during the pandemic?
    - Discuss what risks they would consider when thinking about attending and what would give them more confidence to visit if appropriate
- Do you have any concerns about possible delays in getting treatment for other illness due to pandemic and lockdown?

Screening

- Do you take part in any regular screening programmes? Which ones?
- Should you have been invited for any screening since the start of lockdown in March? Was your screening put on hold or delayed at all?
    - Discuss what happened with their screening, how they feel about postponing/delays and whether this makes them think any differently about screening at all if appropriate
- What do you think would have been the best way to go about screening during the pandemic and lockdown?
    - Discuss whether they believe it should have gone ahead as usual, whether they would have attended as usual/for the first time, whether they would have had any concerns/felt safe and would they expect a delay in their results if appropriate
- Has the pandemic and lockdown changed the way you think about screening in any way?
- What do you think would encourage people to consider taking part in screening again?
- If we invite people who have been waiting or had their screening delayed during the pandemic and lockdown how do you think it should be prioritised?

Health behaviours and Prevention

[Confirm smoking status]

- Has your smoking changed at all during the pandemic and lockdown?
    - Discuss what changed, how, why and when if appropriate
- What do you think about the links which have been shown between heavier body weight and the coronavirus infection?
- What do you think about the links between heavier body weight and other serious illnesses?

Health messaging

- During the pandemic and lockdown where did you get most of your health information from and why?
- How useful and believable did you find these information sources?
    - Discuss which information sources gave them the most confidence in knowing what to do during the pandemic and lockdown regarding health/safety if appropriate
- How useful was social media to you? Did you use them to find or access information at all?
- How did your views of health information change (if at all) during the pandemic and lockdown?

For peer review only

    - Discuss whether they felt that viewed anything unreliable about health/healthcare or COVID-19 and whether they were suspicious of 'fake news' at anytime and how they acted upon these if appropriate
- Did you discuss the health messages you have seen with anyone in person or online?

Closing

- What was the most important thing to consider for your own health (and your family/friends if applicable) throughout the pandemic and lockdown? Did anything influence your thoughts about this?

  [Thank the participant for their time and check if there is anything they would like to expand on or anything they would like to mention that we have not discussed]

For peer review only

For peer review only - http://bmjopen.bmj.com/site/about/guidelines.xhtml

Table S4: Help-seeking interval by symptom

Data are n (%) and unweighted unless otherwise stated

Symptom	Had symptom[1]	Contacted the GP	Within 1 week[2]	Within 2 weeks[2]	Within 1 month[2]	Within 6 weeks[2]	Within 3 months[2]	Within 6 months[2]	Contacted within recommended interval[a,b,3]
	n / 7,543 (%)	n / S (%)	n / S (%)	n / S (%)	n / S (%)	n / S (%)	n / S (%)	n / S (%)	n / S (%)
**Non-specific symptom**									
A persistent change in bowel habits[a]	541 (7.2)	254 (47.0)	55 (10.2)	51 (9.4)	42 (7.8)	30 (5.5)	24 (4.4)	52 (9.6)	148 (27.4)
A persistent change in bladder habits[a]	450 (6.0)	227 (50.4)	65 (14.4)	43 (9.6)	32 (7.1)	23 (5.1)	29 (6.4)	35 (7.8)	140 (31.1)
Tired all the time[a]	1,603 (21.3)	540 (33.7)	92 (5.7)	79 (4.9)	95 (5.9)	58 (3.6)	81 (5.1)	135 (8.4)	266 (16.6)
Persistent unexplained pain[a]	662 (8.8)	361 (54.5)	74 (11.2)	68 (10.3)	59 (8.9)	39 (5.9)	52 (7.9)	69 (10.4)	201 (30.4)
**Non-specific/Red flag symptom**									
Unexplained weight loss[a]	395 (5.2)	179 (45.3)	48 (12.2)	45 (11.4)	43 (10.9)	19 (4.8)	10 (2.5)	14 (3.5)	136 (34.4)
**Red flag symptom**									
A change in the appearance of a mole[b]	391 (5.2)	157 (40.2)	37 (9.5)	34 (8.7)	27 (6.9)	18 (4.6)	20 (5.1)	21 (5.4)	71 (18.2)
An unexplained lump or swelling[b]	422 (5.6)	239 (56.6)	81 (19.2)	55 (13.0)	42 (10.0)	11 (2.6)	19 (4.5)	31 (7.3)	136 (32.2)
Unexplained bleeding[b]	267 (3.5)	143 (53.6)	55 (20.6)	22 (8.2)	22 (8.2)	22 (8.2)	8 (3.0)	14 (5.2)	77 (28.8)
A persistent difficulty swallowing[b]	237 (3.1)	128 (54.0)	26 (11.0)	26 (11.0)	31 (13.1)	12 (5.1)	11 (4.6)	22 (9.3)	52 (21.9)
A sore that does not heal[b]	291 (3.9)	128 (44.0)	34 (11.7)	27 (9.3)	22 (7.6)	12 (4.1)	13 (4.5)	20 (6.9)	61 (21.0)

For peer review only - http://bmjopen.bmj.com/site/about/guidelines.xhtml

**Red flag/Lung-specific symptom**									
Coughing up blood[b]	114 (1.5)	67 (58.8)	32 (28.1)	11 (9.6)	10 (8.8)	5 (4.4)	5 (4.4)	4 (3.5)	43 (37.7)
**Lung-specific symptom**									
Shortness of breath[a]	1,052 (13.9)	484 (46.0)	123 (11.7)	69 (6.6)	100 (9.5)	50 (4.8)	49 (4.7)	93 (8.8)	292 (27.8)
Persistent hoarseness[a]	200 (2.7)	96 (48.0)	25 (12.5)	18 (9.0)	19 (9.5)	13 (6.5)	8 (4.0)	13 (6.5)	62 (31.0)
A persistent cough[a]	444 (5.9)	230 (51.8)	52 (11.7)	40 (9.0)	46 (10.4)	21 (4.7)	29 (6.5)	42 (9.5)	138 (31.1)
A change in an existing cough[a]	196 (2.6)	100 (51.0)	31 (15.8)	24 (12.2)	19 (9.7)	17 (8.7)	5 (2.6)	4 (2.0)	74 (37.8)

n=number, n/S = number of participants representing each symptom presentation behaviour/number of participants who had this symptom.

[a] Recommended interval <1 month, [b] Recommended interval <2 weeks based on previous studies of cancer symptom presentation behaviour (e.g. Herbert et al., 2018 https://www·thelancet·com/journals/lanchi/article/PIIS2352-4642(18)30004-X/fulltext) and aligned with cancer awareness campaigns (e.g. Be Clear on Cancer https://www·cancerresearchuk·org/health-professional/awareness-and-prevention/be-clear-on-cancer). [1] Denominator includes those who did not have a symptom and those who preferred not to say (around 1% of the sample). [2] Interval contacted GP from noticing the symptom. [3] Subset of participants who ‘Contacted the GP in the last 6 months’.

For peer review only - http://bmjopen.bmj.com/site/about/guidelines.xhtml

Table S5: Definitions of codes identified during qualitative interviews with participants and mapping of these themes of symptom experiences, fear of help-seeking and experiences of help-seeking

Themes	Codes	Code definitions
Symptom Experiences	The impact of the pandemic and/or lockdown on general health	Any discussion or reference relating to if and how the pandemic and/or lockdown has impacted the participants general health. Includes changes on beliefs, feelings, views and concerns – relating to both physical and mental health
Symptom Experiences	Symptom experience and recognising changes in health or body	Any discussion or reference to the participant recognising changes to their health or body during the pandemic.
Experiences of help-seeking	Actions taken, or not taken, due to changes in health or body	Any discussion or reference made by the participant to actions taken due to changes in their health or body. Includes healthcare and non-healthcare events and information such as who did they see or talk to about their concern. Why, how and when the participant decided to act, or not act, on any changes
Symptom Experiences & Experiences of help-seeking	Importance of help-seeking	Any discussion or reference made on the relevant importance of help-seeking, especially in reference to the pandemic. Whether the pandemic has affected and/or changed how they think about the importance of help-seeking or rationalise it. Includes both medical and non-medical help-seeking
Fear of help-seeking & Experiences of help-seeking	Concerns about help-seeking	Any discussion or reference to the participant having concerns about help-seeking due to the pandemic. This includes healthcare appointments (primary and secondary care) and virtual and remote help-seeking avenues
Experiences of help-seeking	Experience of face-to-face and virtual/remote consultations	Any discussion or reference to participants experience of a face-to-face or virtual/remote healthcare consultation during the pandemic. Includes both primary and secondary healthcare consultations and views on the differences in help/advice/care compared to pre-pandemic
Experiences of help-seeking	Support and/or encouraging help-seeking and attending healthcare	Any discussion or reference to views/suggestions/ideas provided by participants to support and/or encourage help-seeking and attending healthcare (i.e. possible future facilitators)

For peer review only - http://bmjopen.bmj.com/site/about/guidelines.xhtml

STROBE Statement—Checklist of items that should be included in reports of ***cross-sectional studies***

	Item No	Recommendation	Page No
**Title and abstract**	1	(*a*) Indicate the study's design with a commonly used term in the title or the abstract	1
		(*b*) Provide in the abstract an informative and balanced summary of what was done and what was found	2
**Introduction**			
Background/rationale	2	Explain the scientific background and rationale for the investigation being reported	4-5
Objectives	3	State specific objectives, including any prespecified hypotheses	5
**Methods**			
Study design	4	Present key elements of study design early in the paper	5
Setting	5	Describe the setting, locations, and relevant dates, including periods of recruitment, exposure, follow-up, and data collection	5-6
Participants	6	(*a*) Give the eligibility criteria, and the sources and methods of selection of participants	5
Variables	7	Clearly define all outcomes, exposures, predictors, potential confounders, and effect modifiers. Give diagnostic criteria, if applicable	6-8
Data sources/ measurement	8*	For each variable of interest, give sources of data and details of methods of assessment (measurement). Describe comparability of assessment methods if there is more than one group	6-8
Bias	9	Describe any efforts to address potential sources of bias	5-8
Study size	10	Explain how the study size was arrived at	8
Quantitative variables	11	Explain how quantitative variables were handled in the analyses. If applicable, describe which groupings were chosen and why	6-8
Statistical methods	12	(*a*) Describe all statistical methods, including those used to control for confounding	6-8
		(*b*) Describe any methods used to examine subgroups and interactions	n/a
		(*c*) Explain how missing data were addressed	9
		(*d*) If applicable, describe analytical methods taking account of sampling strategy	n/a
		(*e*) Describe any sensitivity analyses	n/a
**Results**			
Participants	13*	(a) Report numbers of individuals at each stage of study—eg numbers potentially eligible, examined for eligibility, confirmed eligible, included in the study, completing follow-up, and analysed	9-19, supp. Mat.
		(b) Give reasons for non-participation at each stage	Supp. Mat.
		(c) Consider use of a flow diagram	Fig 1
Descriptive data	14*	(a) Give characteristics of study participants (eg demographic, clinical, social) and information on exposures and potential confounders	9-11
		(b) Indicate number of participants with missing data for each variable of interest	9-19
Outcome data	15*	Report numbers of outcome events or summary measures	9-19

For peer review only

Main results	16	(*a*) Give unadjusted estimates and, if applicable, confounder-adjusted estimates and their precision (eg, 95% confidence interval). Make clear which confounders were adjusted for and why they were included	14-19, Table 3
		(*b*) Report category boundaries when continuous variables were categorized	Table 3
		(*c*) If relevant, consider translating estimates of relative risk into absolute risk for a meaningful time period	n/a
Other analyses	17	Report other analyses done—eg analyses of subgroups and interactions, and sensitivity analyses	n/a
**Discussion**			
Key results	18	Summarise key results with reference to study objectives	23
Limitations	19	Discuss limitations of the study, taking into account sources of potential bias or imprecision. Discuss both direction and magnitude of any potential bias	24-25
Interpretation	20	Give a cautious overall interpretation of results considering objectives, limitations, multiplicity of analyses, results from similar studies, and other relevant evidence	23-25
Generalisability	21	Discuss the generalisability (external validity) of the study results	24-25
**Other information**			
Funding	22	Give the source of funding and the role of the funders for the present study and, if applicable, for the original study on which the present article is based	26

*Give information separately for exposed and unexposed groups.

Note: An Explanation and Elaboration article discusses each checklist item and gives methodological background and published examples of transparent reporting. The STROBE checklist is best used in conjunction with this article (freely available on the Web sites of PLoS Medicine at http://www.plosmedicine.org/, Annals of Internal Medicine at http://www.annals.org/, and Epidemiology at http://www.epidem.com/). Information on the STROBE Initiative is available at www.strobe-statement.org.

For peer review only

For peer review only - http://bmjopen.bmj.com/site/about/guidelines.xhtml
